# Deep embedded clustering generalisability and adaptation for integrating mixed datatypes: two critical care cohorts

**DOI:** 10.1038/s41598-024-51699-z

**Published:** 2024-01-10

**Authors:** Jip W. T. M. de Kok, Frank van Rosmalen, Jacqueline Koeze, Frederik Keus, Sander M. J. van Kuijk, José Castela Forte, Ronny M. Schnabel, Rob G. H. Driessen, Thijs T. W. van Herpt, Jan-Willem E. M. Sels, Dennis C. J. J. Bergmans, Chris P. H. Lexis, William P. T. M. van Doorn, Steven J. R. Meex, Minnan Xu, Xavier Borrat, Rachel Cavill, Iwan C. C. van der Horst, Bas C. T. van Bussel

**Affiliations:** 1 Department of Intensive Care Medicine, Maastricht University Medical Centre+, P. Debyelaan, 25, 6229 HX Maastricht, The Netherlands; 2 Cardiovascular Research Institute Maastricht (CARIM), Maastricht University, Maastricht, The Netherlands; 3 Department of Critical Care, University Medical Centre Groningen, University of Groningen, Groningen, The Netherlands; 4 Department of Clinical Epidemiology and Medical Technical Assessment, Maastricht University Medical Centre+, Maastricht, The Netherlands; 5 Department of Clinical Pharmacy and Pharmacology, University Medical Center Groningen, University of Groningen, Groningen, The Netherlands; 6 Bernoulli Institute for Mathematics, Computer Science and Artificial Intelligence, University of Groningen, Groningen, The Netherlands; 7 Department of Cardiology, Maastricht University Medical Centre+, Maastricht, The Netherlands; 8 School of Nutrition and Translational Research in Metabolism (NUTRIM), Maastricht University, Maastricht, The Netherlands; 9 Department of Clinical Chemistry, Central Diagnostic Laboratory, Maastricht University Medical Center, Maastricht, The Netherlands; 10 Takeda Pharmaceuticals, Deerfield, IL USA; 11 Department of Biostatistics Harvard T.H, Chan School of Public Health, Boston, MA USA; 12 Anaesthesiology and Critical Care Department, Hospital Clinic de Barcelona, Barcelona, Spain; 13 Medical Informatics Department, Hospital Clinic de Barcelona, Barcelona, Spain; 14 Department of Advanced Computing Sciences, Maastricht University, Maastricht, The Netherlands; 15 Care and Public Health Research Institute (CAPHRI), Maastricht University, Maastricht, The Netherlands

**Keywords:** Data integration, Machine learning, Medical research

## Abstract

We validated a Deep Embedded Clustering (DEC) model and its adaptation for integrating mixed datatypes (in this study, numerical and categorical variables). Deep Embedded Clustering (DEC) is a promising technique capable of managing extensive sets of variables and non-linear relationships. Nevertheless, DEC cannot adequately handle mixed datatypes. Therefore, we adapted DEC by replacing the autoencoder with an X-shaped variational autoencoder (XVAE) and optimising hyperparameters for cluster stability. We call this model “X-DEC”. We compared DEC and X-DEC by reproducing a previous study that used DEC to identify clusters in a population of intensive care patients. We assessed internal validity based on cluster stability on the development dataset. Since generalisability of clustering models has insufficiently been validated on external populations, we assessed external validity by investigating cluster generalisability onto an external validation dataset. We concluded that both DEC and X-DEC resulted in clinically recognisable and generalisable clusters, but X-DEC produced much more stable clusters.

## Introduction

The Intensive Care Unit (ICU) accommodates the most heterogenous patient population with the highest mortality rate within the hospital^1^. These patients are placed within the same area of the hospital because they require extensive and specialised care, which is effective, but also expensive^2^. However, these patients should ideally be divided into more homogeneous subgroups with specific needs and care pathways. This could improve efficient usage of scarce resources and specialised care, as well as reduce alarm fatigue by separating patients that require more attention. Clustering of ICU patients is increasingly recognised for its potential to improve critical care^3–8^ as clustering analyses can identify more homogenous groups within a heterogeneous population^9^. Furthermore, such homogenous subgroups can also be useful for clinical research, as cluster-specific effects might be diluted by patient heterogeneity^10^.

The many variables that can be collected using continuous multimodal monitoring, which is standard at the ICU, include patient characteristics, clinical variables, co-morbidities, medical history, and laboratory measurements. ICU personnel cannot possibly assess this torrent of information continuously for every patient. Therefore, clinical decision support systems, including machine learning models to aid daily practice, are getting increasingly common^11^. Clustering is one form of such machine learning tools that can stratify patients into more homogenous groups that can be targeted more precisely. Many different clustering techniques exist, but this study focuses on Deep Embedded Clustering (DEC), as employed by Castela Forte et al.^3^ to cluster ICU patients. DEC is a clustering technique that can cluster ICU patients based on large sets of variables. One benefit of DEC is that it can identify non-linear relationships between variables, potentially revealing clusters with clinically recognisable phenotypes that other linear approaches cannot identify^3^.

DEC uses an autoencoder, which is a neural network that can summarise large numbers of variables into a reduced set of latent features. DEC uses those latent features to identify clusters. It uses K-means to identify initial cluster centroids, then optimises the encoder of the autoencoder and updates the cluster centroids to form the clusters. DEC was created by Xie et al.^12^ to cluster images and text. Consequently, DEC was not designed to handle mixed datatypes appropriately, meaning variables on different scales can disproportionately impact on the model. Therefore, we adapted DEC to integrate numerical and categorical variables, both abundant in the ICU, by incorporating an X-shaped variational autoencoder (XVAE) into its architecture. We call this adapted model “X-DEC”. XVAE has two primary differences compared to the original autoencoder. First, it uses a variational autoencoder, a generative model, which, rather than only learning how to compress the input data, also learns the parameters of the input data distribution through variational Bayesian inference. As the encoding distribution is regularised, it can generate new samples, and the chance of overfitting is reduced^13–16^. Second, XVAE uses an X-shaped neural network, which integrates two separate sets of variables^13^. Furthermore, we optimised the X-DEC hyperparameter values for cluster stability through a grid search.

A prerequisite for implementing cluster-driven care and research is proper investigation of cluster validity^17^. Cluster generalisability is often ignored as a crucial aspect of cluster validity, which assesses whether clustering models can identify similar clusters in other comparable populations^3,10,18^. The objective of this study is to evaluate the validity of ICU patient clusters identified by (X-)DEC through the evaluation of its cluster stability, clinical recognisability, and generalisability on an external dataset. Therefore, we chose to replicate the DEC model of Castela Forte et al.^3^ and created the adapted X-DEC model. Both models were trained on data from Castela Forte et al.^3^ and externally validated on data from another hospital. We hypothesise that X-DEC can identify more stable clusters compared to DEC in a well-defined prospective cohort of ICU patients and that these clusters are generalisable to real-world ICU data of another hospital. This study is intended for clinicians as well as data scientists, as we believe it is important for clinicians to be able to follow scientific advances in their field. To ensure readability for a wider audience, this study assumes no prior knowledge of the used methods and explains concepts plainly and visually with minimal jargon. More detailed descriptions of the methods can be found in the supplementary information.

### Related work

This study expands on previous evidence that did not address the generalisability of clustering models. Cluster validation on a separate validation set, and the assessment of cluster generalisability in particular, is often lacking in clustering research^3,10,18^. However, some have recognised its importance^4–6,28–31^, although often focussing on reproducibility rather than generalisability^6,29^, or without using external data^7,30^. Furthermore, we built upon the growing body of research on deep clustering^3,12,23,32–34^ by creating X-DEC.

## Methodology

To investigate cluster generalisability, we recreated the DEC model published by Castela Forte et al.^3^ by training it on data from the original cohort and investigated the generalisability of this model by applying it directly to a retrospective ICU dataset from another hospital. We evaluated whether the DEC model could be improved, first, by replacing its multilayer perceptron (MLP) autoencoder with an X-shaped variational autoencoder (XVAE) that can handle a mixture of numerical and categorical variables more appropriately and second, by optimising its hyperparameters for cluster stability.

### The data

The first cohort was used to create the development dataset, and the second for the external validation dataset. The development dataset originates from the Simple Intensive Care Studies (SICS) cohort, a prospective ICU cohort from the University Medical Centre Groningen (UMCG), Groningen, the Netherlands, described extensively elsewhere^3,19–21^. The external validation dataset is a retrospective ICU dataset from the Maastricht University Medical Centre+ (MUMC+), Maastricht, the Netherlands. We extracted the MUMC+ dataset from a real-world clinical ICU database to reproduce the SICS cohort. Therefore, all unplanned patients admitted to the ICU between 9/7/2012 and 14/3/2020 (to exclude the COVID-19 pandemic patients) were selected (N = 6328). Patients without a registered heart rate (i.e., empty files or patients who died before admission), without known admission type, who underwent elective or planned surgery, aged below 18 years, and patients with a length of stay (LOS) shorter than 24 h (N = 1864) were excluded. Also, medium care patients (N = 523) were excluded (Sipplementary Fig. S1). Each patient sample in this study is a unique admission, meaning that one patient could occur more than once if admitted to the ICU multiple times. Patients in the SICS cohort gave informed consent, which was waived for the MUMC+ cohort by the institutional review board (Medisch-ethische Toetsingscommissie) of the MUMC+. Investigation of the SICS and MUMC+ cohort was approved by the institutional review board of the UMCG (M15.168207) and of the MUMC+ (2021-279), respectively. All methods were performed in accordance with the relevant guidelines and regulations.

All 26 routinely collected laboratory measurements in the SICS cohort were considered for inclusion in the MUMC+ dataset. However, arterial point-of-care measurements were not available in the MUMC+ dataset and were therefore removed from the SICS dataset. Central Venous Pressure (CVP) measurements are invasive and only measured in a few patients. Therefore, CVP was converted into a categorical CVP identifier variable with CVP measurement present (one) or absent (zero). Since positive end-expiratory pressure (PEEP), by definition, is present in mechanically ventilated patients only, this was set to 5 cmH_2_O for non-ventilated patients to mimic low PEEP. The date and time of admission and discharge were used to calculate the LOS. To reduce the risk of data removal due to inaccurate admission times, information gathered within 12 h prior to ICU admission was included. Data after ICU discharge were excluded. Next, variables with more than 40% missing values in the MUMC+ dataset were excluded, removing amylase, mean corpuscular volume (MCV), and troponin T from both datasets (supplementary Fig. S2). Patients with more than 40% missing values for all their variables combined (N = 47) were also excluded^22^. Finally, of the 26 considered laboratory variables, 23 were included (supplementary Table S1).

For all laboratory measurements, the mean and standard deviation of all available measurements during the full ICU stay were computed for each patient sample separately, resulting in 46 variables. Next, missing data were imputed based on Multiple Imputation by Chained Equations using the “miceForest” Python package (see supplementary Imputation section and Table S2). Finally, standard scaling to a z-distribution was applied to all numerical (continuous, discrete, and ordinal) variables from the SICS dataset by subtracting the mean and dividing it by the standard deviation per variable. The mean and standard deviation per variable of the SICS dataset were used to scale the MUMC+ dataset. In addition to 46 laboratory variables (23 means and 23 standard deviations), both datasets included 34 additional variables, resulting in 80 input variables for the cluster analyses (supplementary Table S1) compared to 120 in the original paper^3^. All categorical variables were binary. Furthermore, admission diagnoses, vasoactive medication requirement (binary), LOS, and ICU mortality were outcome variables. The SICS dataset was used to train the clustering models and to assess cluster stability. The MUMC+ dataset was only used to investigate cluster generalisability by applying the clustering models (trained on SICS) to the MUMC+ dataset using all the available input and outcome data (see supplementary descriptive statistics).

The MUMC+ dataset contained 3,894 patient samples (from 3,577 unique patients), compared to 787 patient samples in SICS. Both age and sex did not differ significantly between the two datasets (p = 0.08 and p = 0.77 respectively), with an average age of 63 years and 64% males in MUMC + compared to 62 years and 63% males in SICS. The percentage of patient samples that required vasoactive medication and LOS were significantly higher in MUMC+ (76% and 7.7 days) compared to SICS (49% and 5.9 days) (p < 0.001 and p = 0.006). ICU mortality was higher in the MUMC+ population (23%) compared to SICS (19%) (p = 0.01) (See supplementary descriptive statistics Table S3).

### The recreated deep embedded clustering model

**Figure 1:**
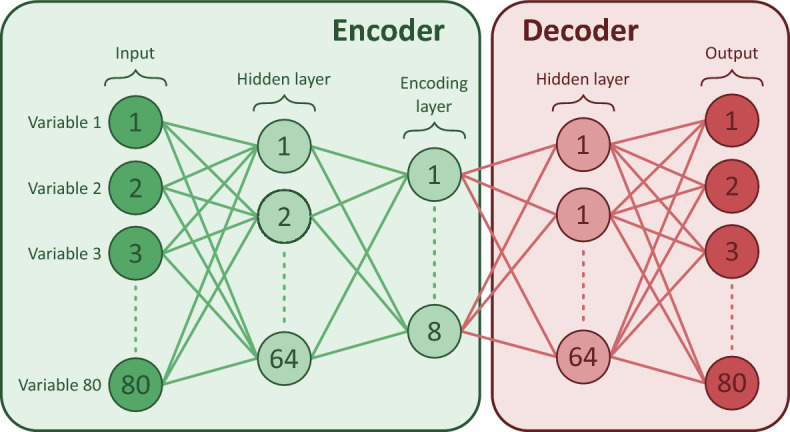
Diagram of the autoencoder used in the original DEC model. Each column of circles is a layer, and the circles are neurons, which essentially are some mathematical combinations of the neurons from the previous layers, except for the input layer, where they represent the original input variables. The solid lines indicate that all neurons can be used to compute all neurons in the subsequent layer. The dotted vertical lines indicate that some neurons are not displayed to simplify the illustration. The numbers represent the number of each neuron. The autoencoder consists of two parts, the encoder (green box), which maps the input data onto a smaller latent feature space used for clustering, and the decoder (red box), which reconstructs the input variables from the latent features. The encoder contains one hidden layer of 64 neurons, which are different combinations of the 80 input variables. The encoder also contains an encoding layer, combining information from the previous 64 neurons in eight neurons (i.e., the latent features). The decoder contains one hidden layer and an output layer that attempts to reconstruct the input variables. Initially, all the connections between the neurons are random. Therefore, the output variables will not resemble the input variables very well. However, the autoencoder is trained by adjusting the weights of the connections between all neurons (i.e., neuron weights) such that the output variables will be as similar as possible to the input variables, as quantified by the mean squared error. It should be noted that the number of layers and the number of neurons in each layer can result in different mappings and thus influence the clustering results.

Because this study aimed to replicate and externally validate the work of Castela Forte et al.^3^, their implementation of the DEC model was followed as precisely as possible unless stated otherwise. First, we recreated the DEC model with six clusters, as Castela Forte et al.^3^ had concluded that this resulted in the most stable clusters, but now using the 80 input variables from the SICS datasets (i.e., “the recreated DEC model”). DEC uses an MLP autoencoder, which is an unsupervised artificial neural network that maps the original input variables into a reduced set of latent features (i.e., synthetic variables). The autoencoder consists of two parts, the encoder, which encodes the input variables into a reduced set of latent features, and the decoder, which reconstructs the input variables from the latent features, ensuring minimal information loss in the encoding (Fig. 1). The autoencoder is trained over 500 epochs. In each epoch, the autoencoder is trained iteratively on batches of 64 patient samples. Ultimately, the encoder of the autoencoder generates latent features that capture as much information from the input variables as possible. Then, DEC uses those latent features to identify clusters, meaning the decoder is only used to train the initial autoencoder.

**Figure 2:**
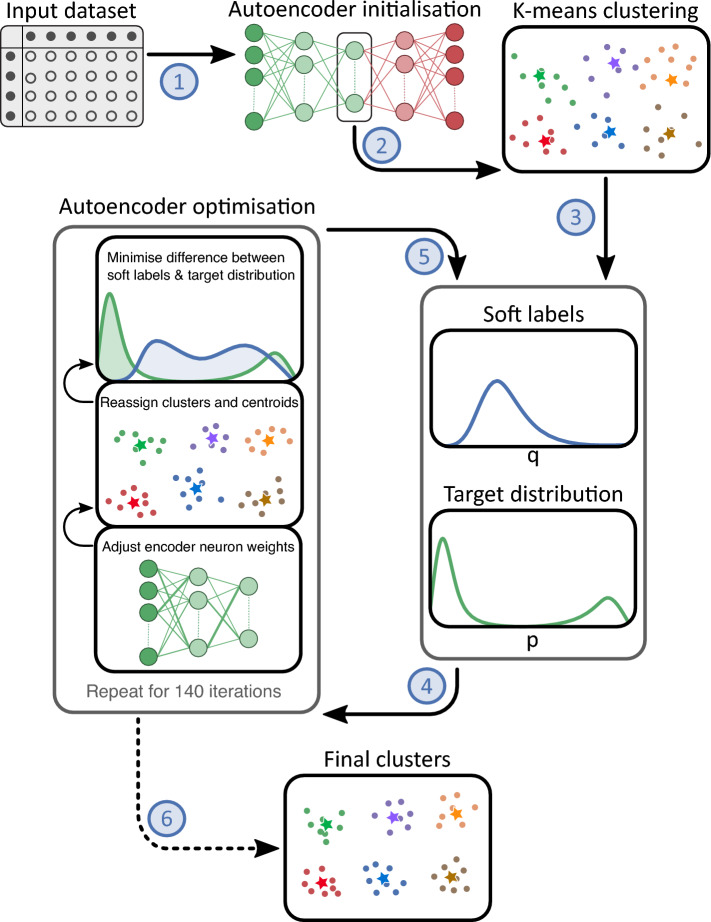
Architecture of the deep embedded clustering algorithm. Based on the input dataset, the autoencoder is initialised and maps the original variables into the latent features (step 1). K-means clustering is performed on the latent features (step 2). Then, six soft labels are computed for each patient sample, and the target distribution is calculated, maximising the separation of high and low soft labels (step 3). Subsequently, the encoder of the autoencoder is optimised to minimise the Kullback–Leibler divergence loss between the soft labels and target distribution over 140 iterations (step 4). If at least 1% of all patient samples change cluster membership, the soft labels and target distribution are recomputed, and the optimisation of the encoder of the autoencoder continues (step 5). Otherwise, clustering is finalised (step 6).

The complete DEC model architecture can be summarised in six steps (Fig. 2). First, to reduce the number of variables, an autoencoder is trained to generate a non-linear mapping of the input variables into latent features (step 1). Second, K-means clustering is performed on the latent features, identifying the initial centroids (the centre points of the clusters) of the six clusters (step 2). Next, the autoencoder is optimised to improve cluster purity, essentially minimising the distance between patient samples within a cluster and maximising the distance between patient samples from different clusters. To this end, soft labels and a target distribution are calculated (step 3). Soft labels are probabilities of how likely a sample belongs to a certain cluster. Each sample has six soft labels, one for each cluster. A high soft label value indicates that a patient is likely to belong to a specific cluster, whereas a low value indicates that the sample is unlikely to belong to that cluster (supplementary Eq. 1). The soft labels are mapped to a target distribution by raising them to the second power, such that the difference between high and low soft labels gets larger, increasing the probability that samples with high soft labels belong to their closest cluster^12^. To prevent larger clusters from distorting the latent features, the target distribution is first divided by the sum per cluster, and then divided by the sum per patient sample, to ensure the target soft labels always add up to one (supplementary Eq. 2). The resulting target distribution portrays a preferable scenario in which the model more confidently assigns cluster labels to patient samples. As this is a hypothetical projection only, the encoder of the autoencoder and the cluster centroids are optimised to produce soft labels more similar to the target distribution (step 4). This is achieved by optimising the encoder to minimise the Kullback–Leibler (KL) divergence loss, which measures the difference between the soft label distribution and target distribution (supplementary Eq. 3). Thereby, forcing highly likely patient samples to move closer to their cluster centroid, while causing less likely patient samples to shift along with them. This optimisation process involves iteratively adjusting the encoder from the autoencoder initialised in step 1. In each iteration, the neuron weights (i.e., how much a neuron, which is a mathematical combination of the input variables, contributes to another neuron in the subsequent layer) are adjusted, resulting in slightly different latent features. Therefore, in every iteration, samples can change cluster membership, and the cluster centroids are recomputed. Then, the KL divergence loss is computed between the target distribution and the new soft labels resulting from the adjusted latent variables and clusters. If the KL divergence loss is getting smaller, the weights will be adjusted in a similar direction in the subsequent iteration; otherwise, different weight adjustments are tested. After each 140^th^ iteration, if at least 1% of all samples changed cluster membership, the cycle continues by recalculating the soft labels and target distribution (step 5). Alternatively, if less than 1% of all samples changed cluster membership, the cycle stops, and the final clusters are determined (step 6). We copied the DEC architecture directly from the original SICS paper^3^, which used previously published code (https://github.com/piiswrong/dec^12^). All clusters from the recreated DEC model were clinically interpreted and described by six ICU consultant physicians of the MUMC+. See supplementary recreated DEC model section for more details on the model.

### The adaptation for mixed data types—the X-DEC model

**Figure 3:**
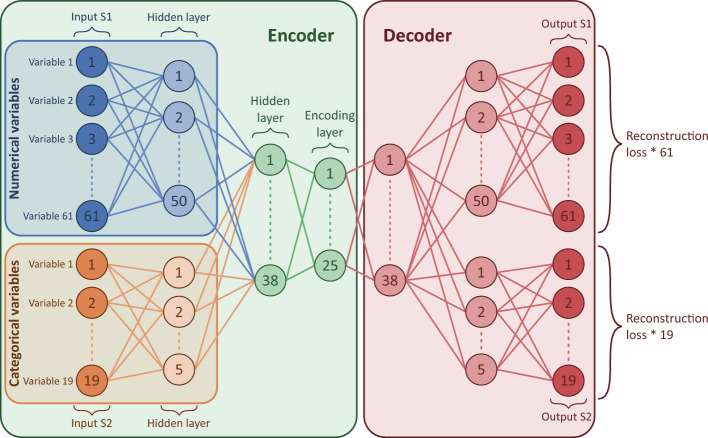
The X-shaped variational autoencoder architecture. Each column of nodes is a neural layer, and the circles it contains are neurons, which essentially are some non-linear mathematical combinations of the neurons from the previous layers. The solid lines show that all neurons can be used to compute all neurons in the next layer. The dotted vertical lines indicate that some neurons are not displayed to simplify the illustration. The numbers represent the number of each neuron. The X-shaped variational autoencoder (XVAE) consists of two main parts. First, the encoder (green box), which maps the input data into the smaller latent feature space, and the decoder (red box), which reconstructs the original variables from the latent features. The input data consists of two separate input sets, input S1 containing all numerical variables (blue box) and input S2 containing all categorical variables (orange box). Each input set is first fed into its own hidden layer. The resulting two hidden layers are then combined into another hidden layer that is fed into the encoding layer (green), which also generates the latent features on which the clustering is performed. The encoding layer uses stochastic inference to approximate the latent features as probability distributions, which in this case are Gaussian. Therefore, the encoding layer is separated into the mean and standard deviation of those distributions (not visualised). Next, the decoder starts where the encoding layer feeds into a hidden layer, which then splits into two separate hidden layers, each feeding into its own output layer to reconstruct the original variables. Finally, the reconstruction loss is determined by computing the mean squared error of the numerical variables and the cross-entropy of the categorical variables, which are both scaled by the number of variables of the input data.

We created the X-DEC algorithm by replacing DEC’s autoencoder with an XVAE to allow the use of both numerical and categorical variables^13^ (Fig. 3). Here, we separate two data types: the numerical variables (containing continuous, discrete, and ordinal variables) and the binary categorical variables. Categorical variables with more than two categories (which were not present in this study) can also be included by converting them to multiple binary dummy variables. XVAE is regularised against overfitting as it enforces a Gaussian prior distribution on the latent features, generally resulting in a better organised latent feature space and potentially more meaningful clusters when used in a DEC framework compared to MLP autoencoders^23^. The code is based on previously published work^13^ (https://github.com/CancerAI-CL/IntegrativeVAEs)*.* Additionally, to circumvent DEC’s arbitrarily selected number of neurons (Fig. 1), hyperparameter tuning was performed to optimise X-DEC for cluster stability. To this end, the X-DEC model was trained using different numbers of neurons in the hidden and encoding layers. The setup with the highest cluster-wise stability was selected (please see the next section on Cluster stability). A full description of the X-DEC model is visualised in supplementary Fig. S3. X-DEC was trained on the SICS dataset, and all resulting clusters were clinically interpreted and described by six ICU consultant physicians of the MUMC+. See supplementary X-DEC model section for more information.

### Cluster stability

For computing cluster stability (i.e., “How often do patient samples end up in the same cluster if we train the clustering model again?”), we created 1000 subsets of the SICS dataset, each containing a random 90% of the patient samples. The clustering model, meaning the entire (X-)DEC pipeline (from step 1 until step 6), excluding hyperparameter optimisation, was trained separately on each of the 1000 subsets. To ensure that the cluster labels of all subsets refer to the same cluster, we ran the model on all patient samples once. We call this the complete model. Then, the cluster labels from each subset were mapped to cluster labels from the complete model based on the highest Jaccard similarity coefficient, a measure of the degree of overlap between two sets of patient samples. The Jaccard similarity coefficient ranges between zero and one, with a higher coefficient representing more similar clustering. The mapping was done iteratively and exclusively, meaning that the cluster with the highest maximum Jaccard similarity coefficient was mapped first and that two clusters from one subset cannot map to the same cluster of the complete set. Finally, each patient sample was assigned a reference cluster label by picking the cluster label assigned most often over all 1000 subsets.

From the clustering results on the 1000 subsets, we computed two types of cluster stability measures. First, the cluster-wise stability was determined using the Jaccard similarity coefficient^24^. The overall Jaccard similarity coefficient was computed by taking the mean of the cluster-wise Jaccard similarity coefficients that were calculated per reference cluster per subset. Secondly, we quantified the sample-wise stability as the percentage of how often a sample ended up in its reference cluster across all 1000 subsets. Stability analyses were performed individually for the DEC and X-DEC models.

### Cluster generalisability

The recreated DEC and X-DEC models, each trained on the SICS dataset, were directly applied to the MUMC+ dataset. The models were not retrained in any way on the MUMC+ dataset, and the weights learned on the SICS dataset were directly applied to the MUMC+ dataset. In other words, if the MUMC+ dataset would contain a patient sample with identical values to a patient sample from the SICS dataset, they would be clustered identically. Then, we assessed the similarity between the MUMC+ and SICS clusters based on the input variables, outcome variables (min–max scaled LOS, average ICU mortality rate, and percentage of admission diagnoses per cluster, while excluding vasoactive medication requirement because it was systematically higher in the MUMC+ dataset), and latent features.

The Gower distance was used to calculate the inter-cluster difference between clusters from the SICS and MUMC+ datasets based on the original input variables. The Euclidean distance was used to calculate the inter-cluster difference based on the outcome variables and latent features. Finally, all MUMC+ clusters were mapped to their most similar SICS cluster based separately on the three distance calculations (input variables, output variables, and latent features), resulting in three mappings per cluster. The mapping was done non-exclusively, meaning multiple clusters from the MUMC+ dataset could map to the same cluster from the SICS dataset. If the clustering model generalises well, the MUMC+ clusters should map to their respective SICS cluster; for example, MUMC+ cluster 1 should map to SICS cluster 1 as these are formed by the same criteria.

## Results

A detailed comparison of the SICS and MUMC+ dataset can be found in supplementary Table S3.

### The recreated DEC model

#### Clusters in the SICS dataset

**Figure 4:**
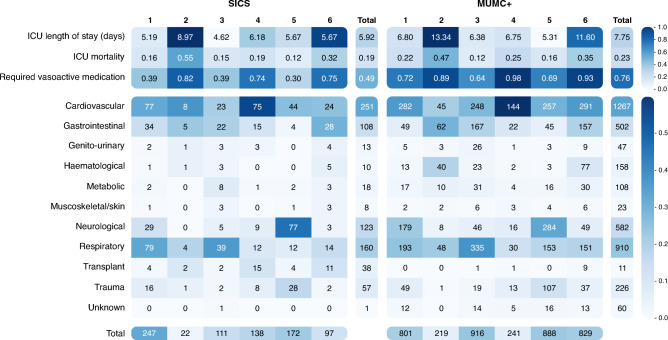
Heatmap of the outcomes and admission diagnoses per cluster from the recreated DEC model. The colour scale, as depicted on the right-hand side of the figure, indicates what fraction of a given cluster belongs to the class specified on the y-axis. The in-cell numbers for ICU length of stay correspond to the mean per cluster in days. The values for ICU mortality and required vasoactive medication depict fraction of non-survivors. Values for diagnoses indicate total counts per cluster. The bar at the bottom shows the total number of patient samples per cluster. The bar at the right indicates the mean (for outcomes) and total number of patient samples (for admission diagnoses) per class across all clusters.

Clusters from the recreated DEC model on the SICS dataset showed differences regarding admission diagnoses and clinical outcomes (Fig. 4). Cluster 1 was the largest and mainly showed cardiovascular and respiratory admission diagnoses. Cluster 2 was the smallest, with only 22 patients, and showed the highest mean LOS and mortality (55%). Cluster 3 had the shortest LOS, a relatively low mortality (15%), and mainly contained respiratory patients. Cluster 4 showed medium mortality (19%) and mainly cardiovascular patients. Cluster 5 had the lowest mortality (12%), containing mainly neurological, cardiovascular, and some trauma patients. Cluster 6 has the second highest LOS and mortality (32%), mainly containing cardiovascular, gastrointestinal, and respiratory patients. See supplementary descriptive statistics Table S4 for more details.

#### Cluster stability

**Figure 5:**
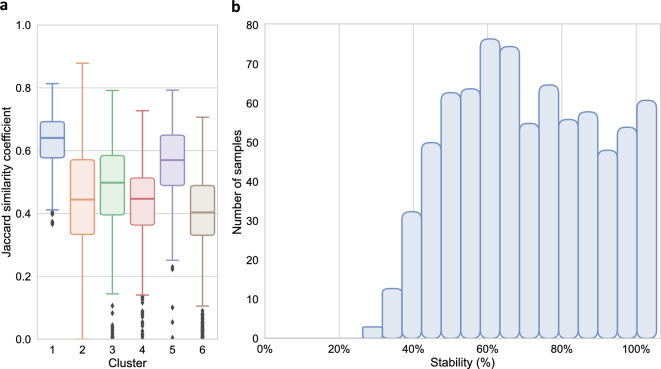
Stability plots of the recreated DEC model on the SICS data set. (**A**) A box-and-whisker plot of the Jaccard similarity coefficients per cluster. (**B**) A bar plot of the sample-wise stability, the y-axis indicates the number of samples in each bar, and the x-axis indicates the stability in terms of how often the samples were clustered into their reference cluster.

The overall Jaccard similarity coefficient of the recreated DEC model was 0.487, with cluster-wise Jaccard similarity coefficients between 0.387 and 0.633 (Fig. 5A). Overall sample-wise stability was 67.2%, and most patient samples had a stability between 40 and 100% (Fig. 5B).

#### Clusters in the MUMC+ dataset

The recreated DEC model applied to the MUMC+ dataset showed differences regarding admission diagnoses and clinical outcomes (Fig. 4). Compared to the SICS dataset, the required vasoactive medication was higher in all clusters, and LOS was longer in all clusters besides cluster 5. Cluster 1 had a medium mortality (22%) and mainly contained cardiovascular, neurological, and respiratory patients. Cluster 2 was the smallest cluster, with only 219 patients, and had the highest mean LOS and mortality (47%). Cluster 3 had the shortest LOS and lowest mortality (12%) and mainly contained respiratory and cardiovascular patients. Cluster 4 had a medium mortality (25%) and contained mainly cardiovascular patients. Cluster 5 had the second-lowest mortality (16%), containing mainly neurological and cardiovascular patients. Cluster 6 had the second-highest mean LOS and mortality (35%), mainly containing cardiovascular, gastrointestinal, and respiratory patients. See supplementary descriptive statistics Table S5 for more details.

#### Cluster generalisability

**Figure 6:**
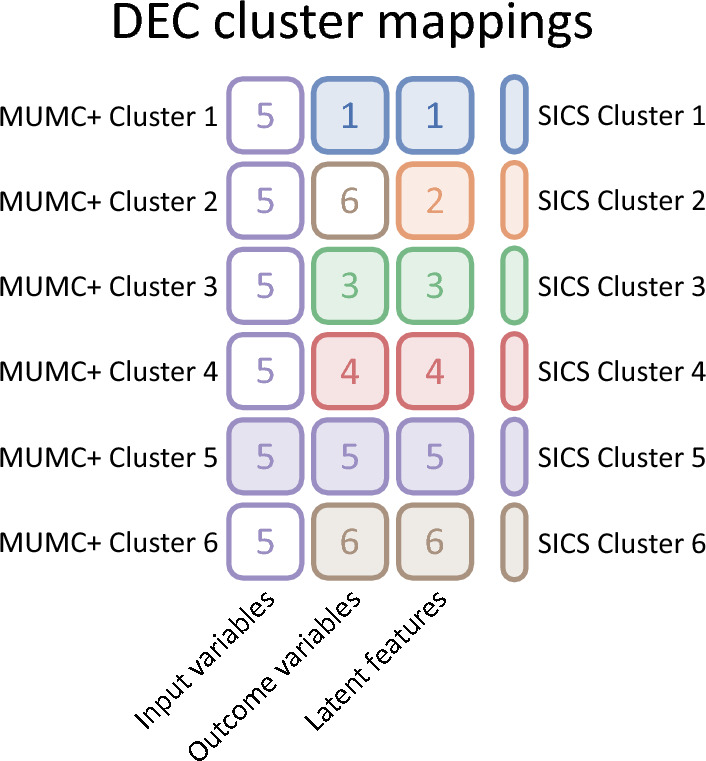
Colour map of generalisability cluster mappings between MUMC+ and SICS clusters from the recreated DEC model based on the input variables, outcomes variables, and the latent feature space. Each row corresponds to an MUMC+ cluster, each column to the variables used for mapping, and each cell colour and number to the SICS cluster it was mapped to, as indicated by the legend on the right.

Based on the input variables, all MUMC+ clusters were most similar to SICS cluster 5, and the mapping on the outcome variables showed that clusters 1, 3, 4, 5 and 6 were mapped correctly. All clusters were mapped correctly based on the latent features (Fig. 6).

### The X-DEC model

#### Clusters in the SICS dataset

**Figure 7:**
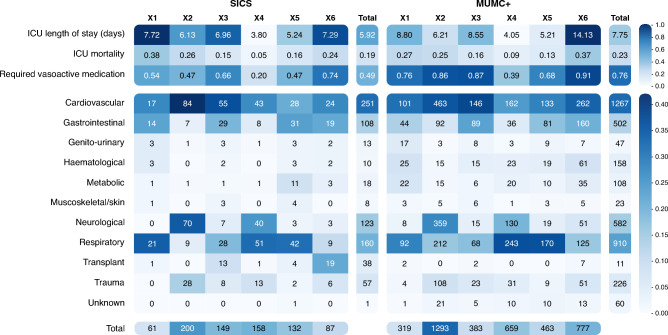
Heatmap of the outcomes and admission diagnoses per cluster from the adjusted X-DEC model. The colour scale, as depicted on the right-hand side of the figure, indicates what fraction of a given cluster belongs to the class specified on the y-axis. The in-cell numbers for ICU length of stay correspond to the mean per cluster in days. The values for ICU mortality and required vasoactive medication depict fraction of non-survivors. Values for diagnoses indicate total counts per cluster. The bar at the bottom shows the total number of patient samples per cluster. The bar at the right indicates the mean (for outcomes) and the total number of patient samples (for admission diagnoses) per class across all clusters.

Clusters from the X-DEC model on the SICS dataset showed differences regarding admission diagnoses and clinical outcomes (Fig. 7). Cluster 1 was the smallest cluster with 61 patients, had the highest mean LOS and mortality (38%), and mainly contained respiratory, cardiovascular, and gastrointestinal patients. Cluster 2 was the largest cluster, with 200 patients and had the second-highest mortality 26%), mainly containing cardiovascular, neurological, and trauma patients. Cluster 3 mainly contained cardiovascular, gastrointestinal, and respiratory patients. Cluster 4 had the lowest mean LOS and mortality (5%), mainly containing respiratory, cardiovascular, and neurological patients. Cluster 5 mainly contained respiratory, gastrointestinal, and cardiovascular patients. Cluster 6 had the second-highest LOS, mainly containing cardiovascular, gastrointestinal, and respiratory patients. See supplementary descriptive statistics Table S6 for more details.

#### Stability

**Figure 8:**
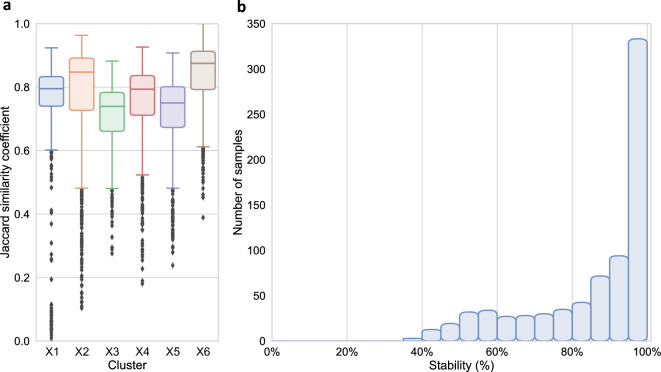
Stability plots of the adjusted X-DEC model on the SICS data set. (**A**) A box-and-whisker plot of the Jaccard similarity coefficients per cluster. (**B**) A bar plot of the sample-wise stability, the y-axis indicates the number of samples in each bar, and the x-axis indicates the stability in terms of how often the samples were clustered into their reference cluster.

The overall Jaccard similarity coefficient of the X-DEC model was 0.762, with cluster-wise Jaccard similarity coefficients between 0.715 and 0.841 (Fig. 8A). Overall sample-wise stability was 85.0%, and most patient samples had a stability above 95% (Fig. 8B).

#### Clusters in the MUMC+ dataset

Clusters from the X-DEC model on the MUMC+ dataset showed differences regarding admission diagnoses and clinical outcomes (Fig. 7). Cluster 1 was the smallest cluster with 319 patients, had the second-highest mean LOS and mortality (27%), and mainly contained cardiovascular, respiratory, and gastrointestinal patients. Cluster 2 was the largest cluster, with 1,293 patients, mainly containing cardiovascular, neurological, and respiratory patients. Cluster 3 mainly contained cardiovascular, gastrointestinal, and respiratory patients. Cluster 4 had the lowest mean LOS and mortality (9%), mainly containing respiratory, cardiovascular, and neurological patients. Cluster 5 had the second-lowest mean LOS and mortality (13%) and mainly contained respiratory, cardiovascular, and gastrointestinal patients. Cluster 6 had the highest mean LOS and mortality (37%), mainly containing cardiovascular, gastrointestinal, and respiratory patients. See supplementary descriptive statistics Table S7 for more details.

#### Cluster generalisability

**Figure 9:**
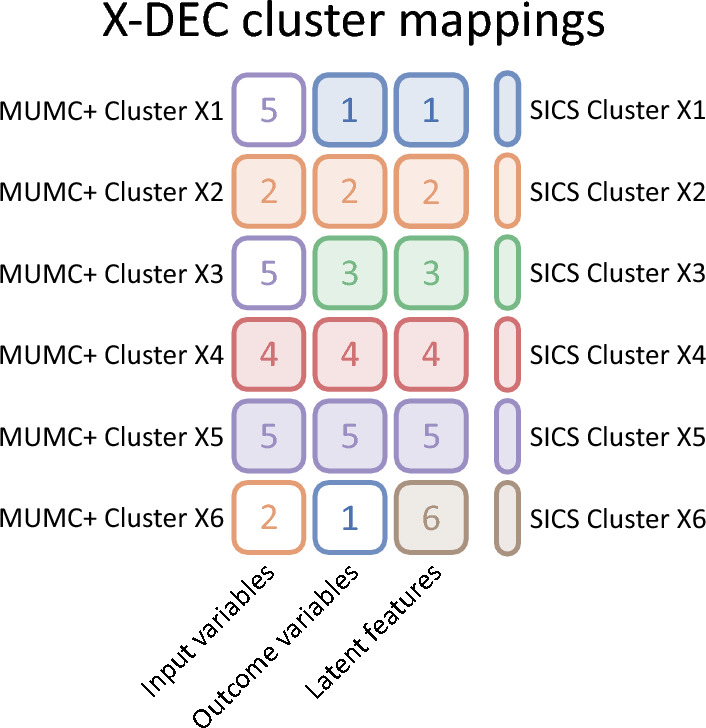
Colour map of cluster mappings between MUMC+ and SICS clusters from the X-DEC model based on the input variables, outcomes variables, and the latent feature space. Each row corresponds to a MUMC+ cluster, each column to the variables used for mapping, and each cell colour and number to the SICS cluster it was mapped to, as indicated by the legend on the right.

Based on the input variables, cluster 2, 4 and 5 were mapped correctly, and the mapping on the outcome variables showed that clusters 2, 3, 4, 5 and 6 were mapped correctly. All clusters were mapped correctly based on the latent features (Fig. 9).

## Discussion

This study introduces X-DEC, a novel adaptation of DEC that can integrate multiple datatypes. This is particularly useful when dealing with clinical data which often contains numeric and categorical variables. We investigated the generalisability and stability of clusters derived from the DEC and X-DEC models, presenting valuable insights into their applicability and advancements in data-driven healthcare, thereby, introducing novel digital health innovations with the potential to improve medical practice and patient care. More specifically, the clusters from both DEC and X-DEC are generalisable as they showed similar clinical phenotypes within clusters when compared between the SICS and MUMC+ datasets, as assessed clinically by physicians as well as data-driven based on the outcome variables. Furthermore, we found that the DEC model was unstable. However, the hyperparameter optimisation and replacement of the MLP autoencoder with an XVAE, which can handle a mixture of data types (numerical and categorical variables in this case) more appropriately, improved cluster stability in the X-DEC model. The generalisability of clustering models on a general ICU patient population shows that clusters could potentially be used for patient stratification in clinical research and, eventually, guide clinicians in designing patient-specific care pathways.

### Clinical interpretation of the DEC clusters

The following DEC cluster phenotypes were identified in both the SICS and MUMC+ dataset. Cluster 1 primarily describes patients with chronic cardiovascular and respiratory conditions. The observed elevation in haematocrit at admission suggests that these patients may have adapted to lower oxygen levels^35^. Furthermore, the high blood pressure in combination with older age suggests a less compliant vascular system^36–38^. High albumin concentrations suggest no acute inflammation and possibly a state of contraction of blood volume affected by diuretics. Cluster 2 appears to include patients with gastrointestinal disease and multiple organ failure, given the high vasopressor support and high concentrations of bilirubin and alkaline phosphatase with varying creatinine and urea concentrations^39^. Cluster 3 may represent patients with bleeding, as those patients had low haemoglobin and haematocrit concentrations and higher urea^40^, although not reflected by higher fibrinogen. However, higher fibrinogen and CRP suggest inflammation, possibly labelling this cluster with sepsis^41^. The latter could also explain why haemoglobin is low due to dilution by major fluid administration in sepsis^42^ and inflammatory bone marrow suppression with subsequent reduction in erythrocyte production. Cluster 4 mostly contains cardiovascular patients with acute hemodynamic impairment, with lower mean and systolic arterial blood pressure, presence of central venous measurements, high vasopressor support and varying magnesium concentrations. This might be related to dysrhythmias and how they are treated^43^. Varying haemoglobin and haematocrit concentrations may reflect optimising cardiac oxygen delivery (e.g., by transfusion). The absence of inflammation suggests no sepsis. Patients in cluster 5 appeared as mainly trauma, neurotrauma and neurological patients. Their admission EMV (Eye opening, best Motor response, best Verbal response) score was low. Many patients were on invasive respiratory support at admission and had low CRP, normal creatinine, and high albumin, suggesting an adequate nutrition status before admission and against intercurrent inflammation. Low potassium could be caused by transmembrane cellular shifts due to targeted mechanical ventilation of neurotrauma patients. The traits of cluster 6 suggest severe multi-organ failure, as those patients stayed relatively long in the ICU with high mortality, having high vasopressor support and low blood pressure, high bilirubin, low thrombocytes, and low albumin. The latter variables suggest multi-organ involvement, possibly caused by a variety of critical diseases^44^.

### Clinical interpretation of the X-DEC clusters

The following X-DEC cluster phenotypes were identified in both the SICS and MUMC+ dataset. Cluster X1 patients had higher urea and higher creatinine concentrations, suggesting kidney injury, paired with higher phosphate and fluctuating calcium concentrations, both suggesting chronic kidney injury. Cluster X2 mainly consists of trauma, neurotrauma and neurological patients with cardiovascular disease. The patients are characterized by low EMV score, high need for mechanical ventilation at admission, and high albumin concentration, suggesting adequate nutrition status before admission and no pre-existing inflammation. Low potassium could be caused by transmembrane cellular shifts due to targeted mechanical ventilation or increased sympathetic drive in neurotrauma patients. Cluster X3 primarily contains patients who appear in shock due to cardiovascular conditions and bleeding as this cluster is mainly characterized by hemodynamic variables, including low systolic blood pressure, presence of central venous measurements, high vasopressor support and varying magnesium concentrations, possibly reflecting dysrhythmias and their treatments, varying haemoglobin and haematocrit concentrations optimising cardiac oxygen delivery (e.g., by transfusion). In addition, the varying fibrinogen, varying haemoglobin, low calcium, and low total protein possibly relate to coagulopathies and bleeding. The high EMV scores indicate that these patients are conscious^45^. Patients in cluster X4 appear to include patients with chronic cardiovascular and respiratory conditions. The increased haematocrit at admission suggests prolonged exposure to lower oxygen levels^35^. Furthermore, the high mean arterial pressure and high diastolic pressure suggest hypertension and an affected cardiovascular system^37^. The high albumin and total protein concentrations indicate no acute inflammation and possibly a state of contraction of blood volume, which is often seen in patients treated with diuretics for heart failure or other cardiovascular conditions. Cluster X5 includes patients who appear to have an infection. They have higher leucocytes and higher CRP concentrations, which fluctuate, suggesting an ongoing inflammatory response^46^. The low diastolic blood pressure and vasopressor support suggest sepsis, whereas high urine output in combination with low potassium, possibly due to urinary loss, may also suggest infection without sepsis. Hence, the relatively low ICU mortality. Cluster X6 includes relatively young patients with severe pre-admission disease, such as transplantation in SICS and haematological and metabolic diseases in MUMC+. Overall, this cluster is characterized by patients who stayed relatively long in the ICU with high vasopressor support and high mortality. They showed varying potassium, low and varying calcium and haematocrit concentrations, and low fibrinogen, total protein, and haemoglobin concentrations.

### Strengths and limitations

The DEC model was unstable, as patients did not consistently end up in the same cluster when retraining DEC multiple times on random subsets of the SICS dataset (only 67.2% on average). However, stability markedly improved in the X-DEC model. Since this study did not show whether X-DEC was more stable due to implementation of XVAE in general, the hyperparameter optimisation, or a combination of both, we ran an additional analysis, where we optimised the hyperparameters of DEC to see if this would improve stability (see supplementary table S9). Although this improved DEC's stability with a Jaccard similarity coefficient of 0.547 and sample-wise stability of 71.4% (supplementary Fig. S4), X-DEC still outperformed DEC, suggesting that the incorporation of XVAE also contributes favourably to its stability. Furthermore, many other hyperparameters of X-DEC could also be optimised, such as the number of epochs, batch size, number of optimisation iterations, and the number of starting points for K-means, among others. However, that is beyond the scope of the present study. Therefore, future research should more elaborately investigate how X-DEC compares to other clustering techniques.

We specifically chose to investigate generalisability by applying a trained clustering model directly to an external dataset rather than testing reproducibility by retraining the model on an external dataset. We expected reproducibility to be poor since the heterogenous population, described by many variables, can be clustered in many ways, where one set of clusters is not more valid than another per se. Furthermore, since the population of the external dataset contains a different collection of patient types (supplementary descriptive statistics Table S3), this suggests that other clusters are likely to be identified. However, when clustering on a more specific dataset, for example, to identify disease subtypes, we also recommend assessing cluster reproducibility on external datasets. For this study, we argue that generalisability is an essential validation aspect that assesses whether cluster phenotypes can be confirmed in another patient population. If clusters are generalisable, this means that clusters, and analyses performed using them, can be directly compared between populations, for example, to compare patient cluster distributions across hospitals.

Clusters from both DEC and X-DEC generalised well onto the MUMC+ dataset, as five out of six MUMC+ clusters mapped to corresponding SICS clusters based on the outcome variables. This suggests that the clusters have comparable phenotypes in both datasets. Only cluster 2 mapped incorrectly to clusters 6, and X6 to X1, possibly because those clusters contain transplant patients, which were underrepresented in the MUMC+ dataset. The requirement of vasoactive medication was excluded from the outcome variables for this mapping because vasoactive medication use was systematically higher in MUMC+ compared to SICS across all clusters. This would lead to most MUMC+ clusters mapping to one SICS cluster with a high requirement of vasoactive medication. Mapping based on the input variables was poor, suggesting that simpler linear clustering techniques like K-means would not find similar clusters. However, the latent features resulted in a perfect mapping, as was hypothesised, since the clusters are based on the latent feature space. Also, the clinical interpretation of the clusters matched well between the two datasets for both models, indicating good generalisability. This validates the clinical recognisability of the clusters for both the DEC and X-DEC model.

This study also has some limitations. Only 80 out of the original 120 variables were available. Therefore, clusters cannot be directly compared to those identified in the paper of Castela Forte et al.^3^. Also, we used relatively general admission diagnoses to represent cluster phenotypes; ideally, more specific discharge diagnoses should be used if available. This could also explain why all clusters contain cardiovascular patients, as more specific diagnoses are unknown. Furthermore, MUMC+ included patient samples in retrospect, whereas SICS was a prospective cohort. This could have caused differences between the populations and, therefore, differences between clusters in the two populations. Nevertheless, clusters were comparable between the two populations. Furthermore, since DEC is optimised to map the variables into a latent feature space, specifically to produce clusters, distance-based internal cluster validity metrics such as the Silhouette score lose their relevance^12^. We have confirmed this with the poor mapping between SICS and MUMC+ clusters based on the input variables and perfect mapping on the latent features. We believe that stability, in combination with careful examination of the clinical cluster properties, is the most valid and suitable approach for evaluating clustering validity here. Additionally, optimising the XVAE hyperparameters for stability introduces a potential pitfall of oversimplification of the clustering model because the simplest clusters (e.g., separated only by binary variables) can also be the most stable clusters. However, those might not be the most clinically meaningful. The clusters identified by the X-DEC model presented in this paper did not exhibit any simple separation, nor did smaller XVAE architectures systematically result in more stable results (see supplementary table S8). This emphasizes the importance of context-specific cluster evaluation and the necessity of an interdisciplinarity approach, combining data science and medicine^17^. Furthermore, there are still some arbitrary aspects in the model with unknown impact on the results. For example, instead of raising the soft labels to the second power to arrive at the target distribution, a different power could be used, altering how much the soft labels are transformed in each iteration. However, this is out of scope for the present study. Additionally, XVAE was the only architecture we evaluated for integrating mixed data types. This approach was chosen because XVAE has been shown to be one of the best-performing and most time-efficient architectures^13^. Other architectures and the inclusion of more data types^13,32,47,48^ could be explored in the future. Since we only evaluated X-DEC and its performance in a specific context, we cannot make any statement on the general performance of X-DEC in other datasets. Future research should further optimise the X-DEC architecture and evaluate its performance in different contexts. It could also be tested if retraining the decoder in combination with the encoder in step 4 (Fig. 2) could improve results and reduce potential overfitting. We also included patient readmissions and, thus, dependent data. This may lead to different but similar samples, where the first admission always survives. However, the inclusion of a variable describing readmissions in the MUMC+ dataset resulted in clusters that were not significantly different when readmissions were excluded (results not shown).

### Implications

The implications of finding overlapping stable clusters in two independent cohorts within an unselected ICU population are significant and might reach far. While the initial discovery of these clusters is promising, it is crucial to validate their existence and characteristics across various healthcare settings and patient populations. Still, identifying and understanding these clusters can provide valuable insights to critical care clinicians. By recognising patterns and subgroups within the ICU population, clinicians can gain a deeper understanding of the underlying pathophysiology and prognosis of their patients. This knowledge can inform their decision-making processes, enabling them to make more accurate diagnoses, choose appropriate treatment strategies, and anticipate potential complications. Finally, the discovery of distinct clusters based on pathophysiological characteristics creates opportunities for more personalised approaches to clinical trials and patient care.

## Conclusion

We validated DEC and the X-DEC adaptation for integrating multiple datatypes on clustering ICU patients. We evaluated the internal- and external validity of a DEC and X-DEC model on ICU patient data. We assessed internal validity based on cluster stability on the development dataset and concluded that DEC was unstable, while X-DEC produced much more stable clusters. External validity was investigated through clinical interpretation and generalisability. The clinical interpretation was based on the development and validation datasets by inspecting input and outcome variables per cluster. We were able to clinically interpret all clusters. To assess cluster generalisability, we made a comparison of clinical cluster interpretations, as well as data-driven mapping of clusters between the two datasets based on the outcome variables. We conclude that cluster phenotypes matched well between the two datasets, suggesting at least some generalisability. Together with the fact that experienced clinicians independently identified overlap in clinical characteristics of the clusters across SICS and MUMC+, this indicates good model generalisability. We presented the data so clinicians who read the paper can redo the cluster phenotype matching between the two datasets by interpreting the results, including supplementary tables S4–S7. Although both the DEC and X-DEC frameworks could identify recognisable and generalisable clusters of ICU patients, we have demonstrated that the shortcomings of the DEC framework can be overcome by X-DEC, resulting in stable, meaningful, and generalisable clusters. This demonstrates the potential for future research into cluster-driven healthcare.

### Software

We used Python version 3.9.7^25^ with the Spyder integrated development environment. The random seed was set to 5192 such that random operations are reproducible. All our code is openly available on GitHub (https://github.com/DAM-IC/Deep-Embedded-Clustering-generalisability-and-adaptation-for-mixed-data-types).

### Compliance with recommendations for machine-learning-related research

We followed the Recommendations for Reporting Machine Learning Analyses in Clinical Research^26^ to ensure complete and transparent reporting of our methodology, results, and interpretation. Since we were unaware of any reporting guideline checklists for clustering analyses, we followed the transparent reporting of a multivariable prediction model for individual prognosis or diagnosis (TRIPOD)^27^ checklist where applicable.

### Supplementary Information

Supplementary Information 1.

Supplementary Table S3.

Supplementary Table S4.

Supplementary Table S5.

Supplementary Table S6.

Supplementary Table S7.

Supplementary Table S8.

Supplementary Table S9.

## Data Availability

The datasets analysed during the current study are not publicly available because they contain sensitive patient information, but access can be provided by the corresponding author on reasonable request and with permission of Maastricht University Medical Centre+ and University Medical Centre Groningen.

## Code availability

The underlying code, documentation, trained models, and dummy data for this research are openly available via a GitHub repository (https://github.com/DAM-IC/Deep-Embedded-Clustering-generalisability-and-adaptation-for-mixed-data-types).

## References

[1] Castela-Forte J, Perner A, van der Horst ICC (2019). The use of clustering algorithms in critical care research to unravel patient heterogeneity. Intens. Care Med..

[2] Costa DK, Kahn JM (2016). Organizing critical care for the 21st century. JAMA.

[3] Castela Forte J (2021). Identifying and characterizing high-risk clusters in a heterogeneous ICU population with deep embedded clustering. Sci. Rep..

[4] Mousai O (2022). Clustering analysis of geriatric and acute characteristics in a cohort of very old patients on admission to ICU. Intens. Care Med..

[5] Sweeney TE (2018). Unsupervised analysis of transcriptomics in bacterial sepsis across multiple datasets reveals three robust clusters. Crit. Care Med..

[6] Papin G (2021). Clinical and biological clusters of sepsis patients using hierarchical clustering. PLoS ONE.

[7] Vranas KC (2017). Identifying distinct subgroups of ICU patients: A machine learning approach*. Crit. Care Med..

[8] Maslove DM (2022). Redefining critical illness. Nat. Med..

[9] Guha S, Rastogi R, Shim K (1998). CURE: An efficient clustering algorithm for large databases. ACM SIGMOD Rec..

[10] Reddy K (2020). Subphenotypes in critical care: Translation into clinical practice. Lancet Respir. Med..

[11] van de Sande D, van Genderen ME, Huiskens J, Gommers D, van Bommel J (2021). Moving from bytes to bedside: A systematic review on the use of artificial intelligence in the intensive care unit. Intens. Care Med..

[12] Xie, J., Girshick, R. & Farhadi, A. Unsupervised Deep Embedding for Clustering Analysis. *arXiv* **10**. 10.48550/arXiv.1511.06335 (2016).

[13] Simidjievski N (2019). Variational autoencoders for cancer data integration: Design principles and computational practice. Front. Genet..

[14] Cemgil, T., Ghaisas, S., Dvijotham, K., Gowal, S. & Kohli, P. The autoencoding variational autoencoder. In *Advances in Neural Information Processing Systems, vol. 33* 15077–15087 (Curran Associates, Inc., 2020).

[15] Kingma DP, Welling M (2019). An introduction to variational autoencoders. Found. Trends Mach. Learn..

[16] Alemi, A. A., Fischer, I., Dillon, J. V. & Murphy, K. *Deep Variational Information Bottleneck*. http://arxiv.org/abs/1612.00410 (2019).

[17] von Luxburg, U., Williamson, R. C. & Guyon, I. Clustering: Science or Art? In *Proceedings of ICML Workshop on Unsupervised and Transfer Learning.* Vol. 27 (eds Guyon, I. *et al.*) 65–79 (PMLR, 2012).

[18] Siepel S (2023). Evolution of clinical phenotypes of COVID-19 patients during intensive care treatment: An unsupervised machine learning analysis. J. Intens. Care Med..

[19] Hiemstra B (2017). Clinical examination, critical care ultrasonography and outcomes in the critically ill: Cohort profile of the Simple Intensive Care Studies-I. BMJ Open.

[20] Hiemstra B (2019). The diagnostic accuracy of clinical examination for estimating cardiac index in critically ill patients: The simple intensive care studies-I. Intens. Care Med..

[21] Hiemstra B (2019). Clinical examination for the prediction of mortality in the critically ill: The simple intensive care studies-I. Crit. Care Med..

[22] Jakobsen JC, Gluud C, Wetterslev J, Winkel P (2017). When and how should multiple imputation be used for handling missing data in randomised clinical trials—a practical guide with flowcharts. BMC Med. Res. Methodol..

[23] Jiang, Z., Zheng, Y., Tan, H., Tang, B. & Zhou, H. *Variational Deep Embedding: An Unsupervised and Generative Approach to Clustering*. http://arxiv.org/abs/1611.05148 (2017).

[24] Hennig C (2007). Cluster-wise assessment of cluster stability. Comput. Stat. Data Anal..

[25] Van Rossum G, Drake FL (2009). Python 3 Reference Manual.

[26] Recommendations for Reporting Machine Learning Analyses in Clinical Research. (2022). 10.1161/CIRCOUTCOMES.120.006556. 10.1161/CIRCOUTCOMES.120.006556 PMC8320533 33079589

[27] Collins GS, Reitsma JB, Altman DG, Moons KGM (2015). Transparent reporting of a multivariable prediction model for individual prognosis or diagnosis (TRIPOD): The TRIPOD statement. Ann. Intern. Med..

[28] Verdonschot JAJ (2023). Clustering of cardiac transcriptome profiles reveals unique. JACC Basic Transl. Sci..

[29] Calfee CS (2014). Subphenotypes in acute respiratory distress syndrome: Latent class analysis of data from two randomised controlled trials. Lancet Respir. Med..

[30] Bhavani SV (2022). Development and validation of novel sepsis subphenotypes using trajectories of vital signs. Intens. Care Med..

[31] Meijs C (2023). Identifying distinct clinical clusters in heart failure with mildly reduced ejection fraction. Int. J. Cardiol..

[32] Yang, L., Cheung, N.-M., Li, J. & Fang, J. Deep clustering by gaussian mixture variational autoencoders with graph embedding. In *2019 IEEE/CVF International Conference on Computer Vision (ICCV)* 6439–6448 (IEEE, 2019). 10.1109/ICCV.2019.00654.

[33] Lutscher, D., Hassouni, A. el, Stol, M. & Hoogendoorn, M. *Mixing Consistent Deep Clustering*. 10.48550/arXiv.2011.01977 (2020).

[34] Min E (2018). A survey of clustering with deep learning: From the perspective of network architecture. IEEE Access.

[35] McGuire M, Bradford A (1999). Chronic intermittent hypoxia increases haematocrit and causes right ventricular hypertrophy in the rat. Respir. Physiol..

[36] Glynn RJ, Chae CU, Guralnik JM, Taylor JO, Hennekens CH (2000). Pulse pressure and mortality in older people. Arch. Intern. Med..

[37] Cohn JN, Finkelstein SM (1992). Abnormalities of vascular compliance in hypertension, aging and heart failure. J. Hypertens. Suppl. Off. J. Int. Soc. Hypertens..

[38] Vaitkevicius PV (1993). Effects of age and aerobic capacity on arterial stiffness in healthy adults. Circulation.

[39] Gill RQ, Sterling RK (2001). Acute liver failure. J. Clin. Gastroenterol..

[40] Ernst AA, Haynes ML, Nick TG, Weiss SJ (1999). Usefulness of the blood urea nitrogen/creatinine ratio in gastrointestinal bleeding. Am. J. Emerg. Med..

[41] Göbel K (2018). The coagulation factors fibrinogen, thrombin, and factor XII in inflammatory disorders—a systematic review. Front. Immunol..

[42] Perel A (2018). The relationship between the decrease in haemoglobin concentration and the volume of fluids administered during resuscitation from septic shock may not be so ‘weak’. Crit. Care Lond. Engl..

[43] Millane TA, Ward DE, Camm AJ (1992). Is hypomagnesemia arrhythmogenic?. Clin. Cardiol..

[44] Vincent JL (1998). Use of the SOFA score to assess the incidence of organ dysfunction/failure in intensive care units: Results of a multicenter, prospective study. Working group on ‘sepsis-related problems’ of the European Society of Intensive Care Medicine. Crit. Care Med..

[45] Teasdale G, Jennett B (1974). Assessment of coma and impaired consciousness: A practical scale. The Lancet.

[46] Johnson HL, Chiou CC, Cho CT (1999). Applications of acute phase reactants in infectious diseases. J. Microbiol. Immunol. Infect. Wei Mian Yu Gan Ran Za Zhi.

[47] Suh, S. & Choi, S. *Gaussian Copula Variational Autoencoders for Mixed Data*. http://arxiv.org/abs/1604.04960 (2016).

[48] Ma, C., Tschiatschek, S., Turner, R., Hernández-Lobato, J. M. & Zhang, C. VAEM: A deep generative model for heterogeneous mixed type data. In *Advances in Neural Information Processing Systems. vol. 33 *11237–11247 (Curran Associates, Inc., 2020).

