# Supplementary material for: Deep embedded clustering generalisability and adaptation for integrating mixed datatypes: two critical care cohorts
Source: Sci Rep. 2024 Jan 10;14:1045. doi: 10.1038/s41598-024-51699-z (PMC10781731; doi:10.1038/s41598-024-51699-z)
Supplement: Supplementary file 1 — Supplementary Information 1. [file 41598_2024_51699_MOESM1_ESM.pdf]

Supplementary information - Deep embedded clustering generalisability and adaptation for integrating mixed datatypes: two critical care cohorts

Jip W.T.M. de Kok[1,2], Frank van Rosmalen[1,2], Jacqueline Koeze[3], Frederik Keus[3], Sander M.J. van Kuijk[4], José Castela Forte[5,6], Ronny M. Schnabel[1], Rob G.H. Driessen[1,2,7], Thijs T.W. van Herpt[1,2], Jan-Willem E.M. Sels[1,2,7], Dennis C.J.J. Bergmans[1,8], Chris P.H. Lexis[1], William P.T.M. van Doorn[2,9], Steven J.R. Meex[2,9], Minnan Xu[10], Xavier Borrat[11,12,13], Rachel Cavill[14], Iwan C.C van der Horst[1,2], Bas C.T. van Bussel[1,2,15]

Affiliations:

1. Department of Intensive Care Medicine, Maastricht University Medical Centre +, Maastricht, the Netherlands
2. Cardiovascular Research Institute Maastricht (CARIM), Maastricht University, Maastricht, the Netherlands
3. Department of Critical Care, University Medical Centre Groningen, University of Groningen, Groningen, The Netherlands
4. Department of Clinical Epidemiology and Medical Technical Assessment, Maastricht University Medical Centre+, Maastricht, The Netherlands
5. Department of Clinical Pharmacy and Pharmacology, University Medical Center Groningen, University of Groningen, the Netherlands
6. Bernoulli Institute for Mathematics, Computer Science and Artificial Intelligence, University of Groningen, the Netherlands
7. Department of Cardiology, Maastricht University Medical Centre +, Maastricht, the Netherlands
8. School of Nutrition and Translational Research in Metabolism (NUTRIM), Maastricht University, Maastricht, the Netherlands.
9. Department of Clinical Chemistry, Central Diagnostic Laboratory, Maastricht University Medical Center, Maastricht, The Netherlands
10. Takeda Pharmaceuticals, Deerfield, Illinois, USA
11. Department of Biostatistics Harvard T.H, Chan School of Public Health, Boston, Massachusetts, United States of America
12. Anaesthesiology and Critical Care Department, Hospital Clinic de Barcelona, Barcelona, Spain
13. Medical Informatics Department, Hospital Clinic de Barcelona, Barcelona, Spain
14. Department of Advanced Computing Sciences, Maastricht University, Maastricht, the Netherlands
15. Care and Public Health Research Institute (CAPHRI), Maastricht University, Maastricht, the Netherlands

Corresponding author:

Jip de Kok, Maastricht University Medical Centre +, Department of Intensive Care Medicine, P. Debyelaan, 25, 6229 HX Maastricht, the Netherlands, jip.de.kok@mumc.nl

Methodology

All analyses were performed in Python (except for the creation of the descriptive statistics tables, which were generated with R version 4.2.1) on a desktop with an AMD Ryzen 9 5950x 32-threaded CPU, Nvidia RTX 3090 GPU, and 64GB of RAM.

The data

The number of samples removed in each data preparation step is summarised in Fig. S1. Table S1 shows which variables were included in this study, compared to the variables that were used in the original study of Castela Forte et al.

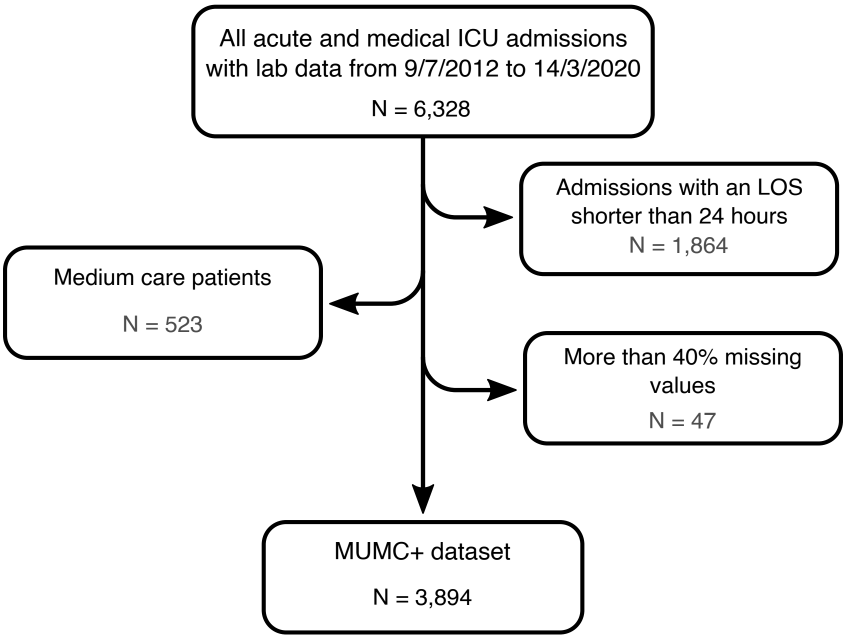

Figure S1. Flowchart illustrating the patient sample selection procedure with the patient sample count per step indicated by the number of patients (N) for the MUMC+ external validation dataset.

Table S1. Clustering variables present in the MUMC+ and SICS datasets in this study compared to the variables of the original SICS cohort. Variables from the original SICS cohort that were excluded in this study are marked in red.

	Clustering variables present in both the SICS and MUMC+ dataset	Original SICS cohort variables
Patient characteristics	age, sex, APACHE IV Score, SAPS II Score, BMI, surgical admission, previous admission to ICU	age, sex, APACHE IV Score, SAPS II Score, BMI, surgical admission, previous admission to ICU
Clinical examination	Hemodynamic parameters<br>atrial fibrillation, heart rate at admission, urine output in previous 6h, central venous pressure (CVP), diastolic blood pressure (DBP), systolic blood pressure (SBP), mean arterial pressure (MAP)<br>Respiratory parameters<br>worsened respiratory condition after 24 h assessed by physician, tidal volume (Vt), Respiratory rate of ventilator, positive end-expiratory pressure (PEEP) of ventilator, mechanical ventilation after 24 h (categorical), mechanical ventilation at admission (categorical), respiratory rate, lowest FiO_2 (%) during ICU stay<br>Other<br>EMV score	Hemodynamic parameters<br>cardiac index, mottling, atrial fibrillation, heart rate at admission, urine output in previous 6h, prolonged capillary refill time, central venous pressure (CVP), diastolic blood pressure (DBP), systolic blood pressure (SBP), mean arterial pressure (MAP)<br>Respiratory parameters<br>worsened respiratory condition after 24 h assessed by physician, tidal volume (Vt), Respiratory rate of ventilator, positive end-expiratory pressure (PEEP) of ventilator, mechanical ventilation after 24 h (categorical), mechanical ventilation at admission (categorical), respiratory rate, lowest FiO_2 (%) during ICU stay<br>Other<br>EMV score
Co-morbidities and medical history (categorical)	history of cardiovascular disease (CVD), history of chronic kidney disease (CKD), history of cirrhosis, history of chronic obstructive pulmonary disease (COPD), history of diabetes, history of haematological malignancy, history of metastatic disease, history of myocardial infarction, history of respiratory insufficiency, history of immune insufficiency, previous dialysis	history of cardiovascular disease (CVD), history of chronic kidney disease (CKD), history of cirrhosis, history of chronic obstructive pulmonary disease (COPD), history of diabetes, history of haematological malignancy, history of metastatic disease, history of myocardial infarction, history of respiratory insufficiency, history of acquired immunodeficiency syndrome (AIDS), history of immune insufficiency, previous dialysis
Laboratory variables (for which the mean and standard deviation were taken over the first 48 since the first measurement)	Routinely collected<br>ALAT, ASAT, albumin, ALP, bilirubin (total), gamma-GT, CK, CRP, calcium, chloride, magnesium, sodium, phosphate, potassium, fibrinogen, haemoglobin, haematocrit, creatinine, LDH, leukocytes, thrombocytes, total protein, urea	Routinely collected<br>ALAT, ASAT, albumin, amylase, ALP, bilirubin (total), gamma-GT, CK, CRP, calcium, chloride, magnesium, MCV, sodium, phosphate, potassium, fibrinogen, haemoglobin, haematocrit, creatinine, LDH, leukocytes, thrombocytes, troponin T, total protein, urea<br>Arterial point of care<br>ionized calcium, glucose, haemoglobin, potassium, lactate, sodium, arterial HCO_3, arterial pCO_2, arterial pH, arterial pO_2, arterial saturation, methylated haemoglobin, HbCO

Imputation

Missing data were imputed based on Multiple Imputation by Chained Equations using the Python package "miceForest." Categorical and discrete variables were imputed with mean matching, each using ten mean matching candidates following the kdtree classification function. All input variables (Table 1) were available for the imputation of each variable. When imputing the mean of a laboratory value, the standard deviation of that laboratory value was excluded in the imputation process and vice versa. Also, for the imputation of a given variable, any other variable that was always missing in conjunction was excluded for the imputation of that given variable. As a result, some variables were excluded for imputing other variables, as indicated in Table S2. The lightbm hyperparameters were optimised using the built-in function of the miceForest

package with ten optimisation steps. Finally, imputation was performed with ten iterations, generating 100 imputed sets. All analyses described in this paper were performed on the first imputed set. The other imputed sets were only used to gain insight into the imputation process. The degree of missingness of each variable for the two datasets prior to imputation is visualised in Fig. S2.

Table S2. Variables excluded for imputing other variables.

Imputed variables	Variables excluded for the imputation
Haemoglobin	Haematocrit
Haematocrit	Haemoglobin
Ureum	Albumin, calcium, chloride, and total protein
FiO2 low	Tidal volume and mechanical respiratory ventilation
Gender	Total protein
MAP	SBP, DBP, Urine output, and magnesium
DBP	SBP, MAP, urine output, and magnesium
SBP	DBP, MAP, urine output, and magnesium

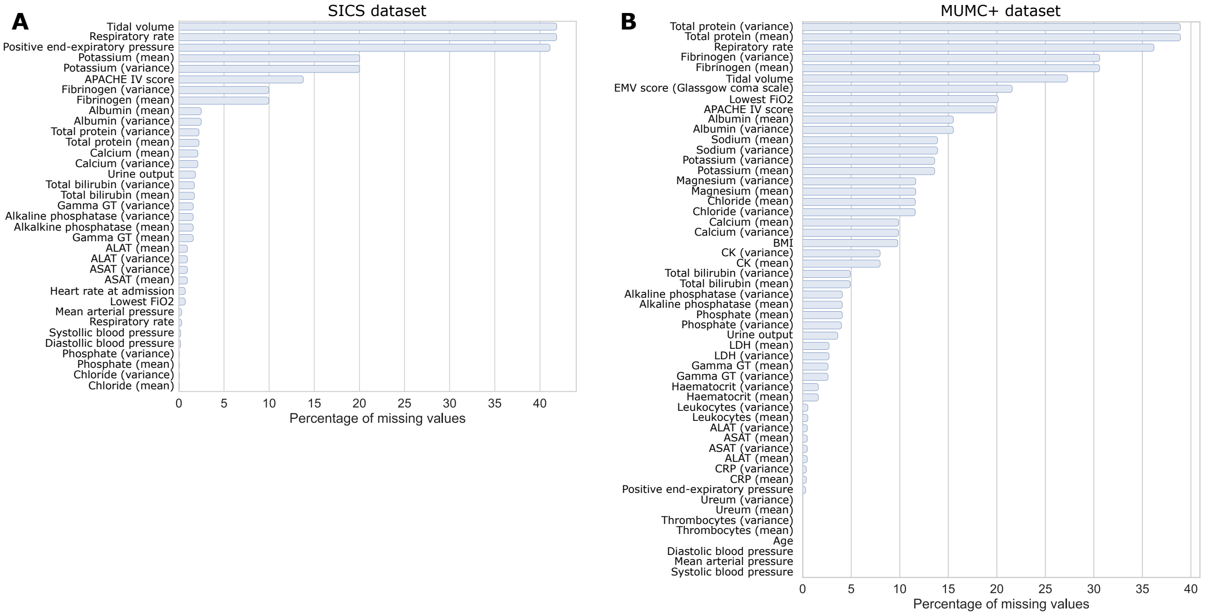

Figure S2. Bar chart of the degree of missingness per variable per dataset. a, The degree of missingness for the variables in the SICS dataset. b, The degree of missingness for the variables in the MUMC+ dataset. The x-axis shows the percentage of how many were missing for a given variable. The y-axis shows the variables for which data was missing. variables without any missing data are not shown.

The recreated DEC model

The recreated DEC model was based on an existing GitHub repository (https://github.com/piiswrong/dec[1]). The Keras and TensorFlow Python packages were used for building the autoencoder. The encoder was set up to contain two dense layers with ReLU activation functions, the first had an l1 regularisation of 10e-5 and 64 neurons, and the second contained 8 neurons. The decoder layer contained one dense layer with ReLU activation with 64 neurons and the final output dense layer with sigmoid activation function. The autoencoder was optimised using the Adam optimiser, minimising the mean squared error as loss score. When fitting the autoencoder, 500 epochs were performed, and the batch size was set to 64. After initialising the autoencoder, K-means was performed on the latent features with 20 initialisations. Subsequently, the soft labels and target distribution were computed. A soft label q_{ij} can be interpreted as the probability of sample i belonging to cluster j. The Student's t-distribution with one degree of freedom was used to compute the similarity between a patient sample z_i and cluster centroid μ_j:

$$q_{ij} = \frac{(1 + \|z_i - \mu_j\|^2)^{-1}}{\sum_{j'}(1 + \|z_i - \mu_{j'}\|^2)^{-1}} \tag{1}$$

The target distribution P was computed by calculating p_i by raising q_i to the second power and normalising by frequency per cluster as follows:

$$p_{ij} = \frac{q_{ij}^2 \,/\, f_j}{\sum_{j'} q_{ij'}^2 \,/\, f_{j'}} \tag{2}$$

Stochastic gradient descent was performed for 140 iterations with a learning rate of 0.01 and momentum of 0.9 on batches of 256 samples, minimising the KL divergence loss between the soft label assignments q_i and the target distribution p_i as follows:

$$L = KL(P\|Q) = \sum_i \sum_j p_{ij} \log \frac{p_{ij}}{q_{ij}} \tag{3}$$

After each 140th iteration, soft labels and target distribution were recomputed, if at least 1% of the patient samples changed cluster membership (up to a maximum of 8,000 iterations), another 140 iterations were performed. More detailed information about the DEC model can be found in the original publication[1].

All DEC results of this study are based on the recreated DEC model, which was trained with the unoptimised hyperparameter values to replicate the model of Castela Forte et al., unless explicitly stated otherwise. As additional analysis we optimised the hyperparameters of the recreated DEC model with a grid search to investigate if this would improve stability. We recomputed the stability of the DEC model with optimised hyperparameter values in the same way we calculated stability for the recreated DEC and X-DEC models, by retraining it on 1,000 subsets containing random 90% of the patient samples. A complete list of the evaluated hyperparameter combinations and their resulting stability scores can be found in the supplementary Table S9. For each combination of hyperparameter values, 10-fold repeated cross-validation was performed with 3 repeats, and the entire DEC pipeline (from step 1 until 6) was performed on each fold. The DEC hyperparameter optimisation was performed to test whether DEC with optimised hyperparameters would match the stability of X-DEC.

The X-DEC model

The X-DEC model contains an X-shaped Variational Autoencoder (XVAE) (Fig. 4), based on previous work[2] (https://github.com/CancerAI-CL/IntegrativeVAEs). The numerical variables (input S1) and categorical variables (input S2) are supplied separately to the model. Both sets have an individual hidden layer, which are subsequently combined into a single hidden layer that feeds into the encoding layer, which produces the latent variables used by the clustering model. The decoder mirrors the encoder in the attempt to recreate the two original input sets. All layers use ELU activation functions except for the output layers, which use a linear activation function for the numerical variables and a sigmoid for the categorical variables. Reconstruction loss is computed as mean squared error for the numerical variables and as binary cross entropy for categorical variables. The reconstruction losses are computed per set and scaled by the number of variables present in each set to mitigate the potential over-representation of variables in the smaller set. The XVAE was trained using the Adam optimiser. The final reconstruction loss is the average of the scaled losses of the two sets. The hyperparameters of XVAE in the X-DEC model were optimised through a grid search. A complete list of the evaluated hyperparameter combinations and their resulting stability scores can be found in the supplementary Table S8, stored in an additional file. For each combination of hyperparameter values, 10-fold repeated cross-validation was performed with 3 repeats, the entire X-DEC pipeline was performed on each fold. Finally, the hyperparameter values that resulted in the highest mean Jaccard stability coefficient were picked, and the final X-DEC model was trained on the full SICS dataset. Hyperparameter optimisation took ~85 hours. For more information on X-DEC, see the publicly available code (https://github.com/DAM-IC/Deep-Embedded-Clustering-generalisability-and-adaptation-for-mixed-data-types).

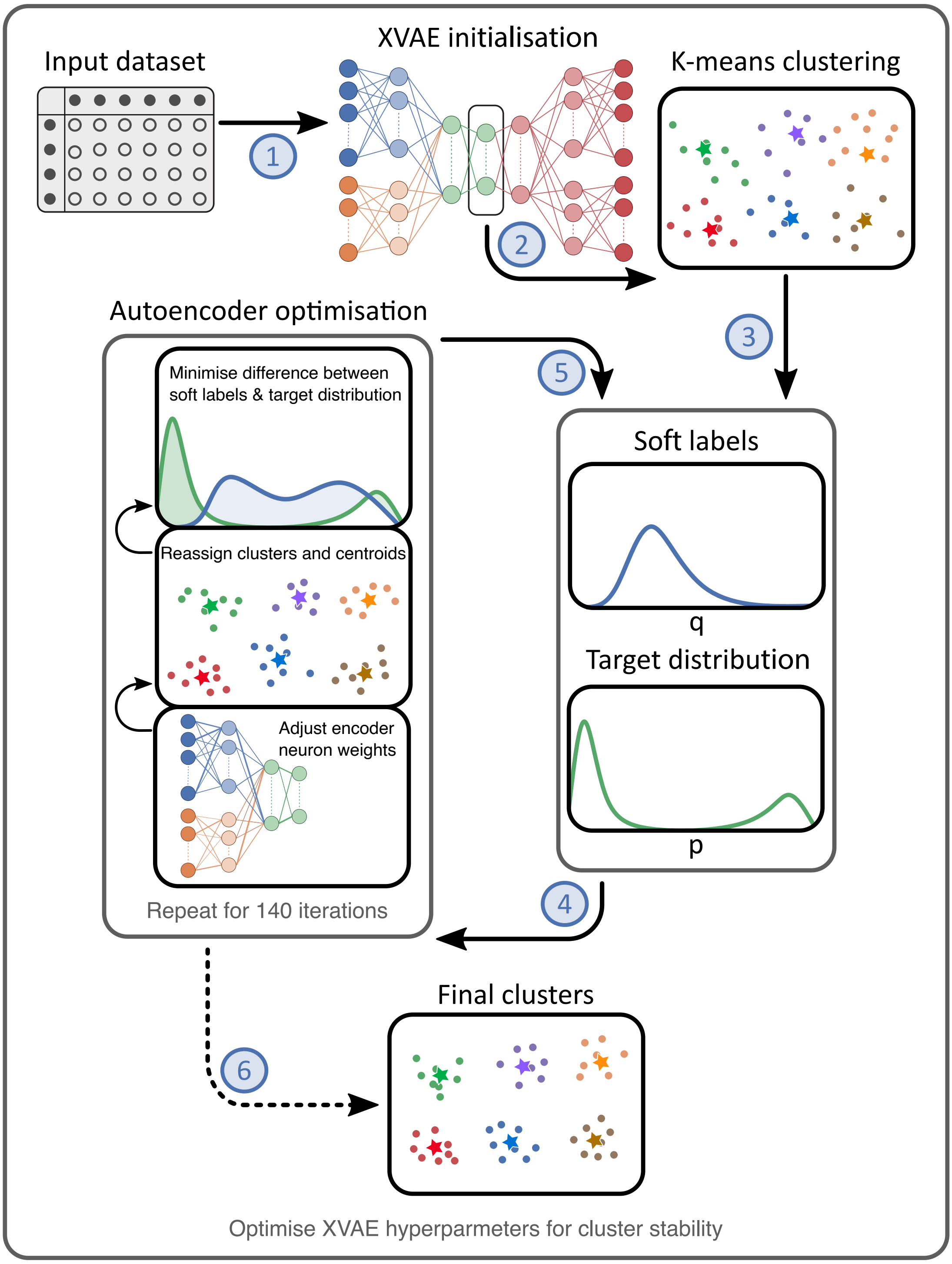

Figure S3. Architecture of the X-DEC algorithm. Based on the input dataset, the X-shaped variational autoencoder (XVAE) is initialised and maps the original variables into the latent features (step 1). K-means clustering is performed on the latent features (step 2). Then, six soft labels are computed for each patient sample, and the target distribution is calculated, maximising the separation of high and low soft labels (step 3). Subsequently, the encoder of the XVAE is optimised to minimise the Kullback-Leibler divergence loss between the soft labels and target distribution over 140 iterations (step 4). If at least 1% of all patient samples change cluster membership, the soft labels and target distribution are recomputed, and the optimisation of the encoder of the XVAE continues (step 5). Otherwise, clustering is finalised (step 6). This procedure is repeated many times for different architectures of the X-shape variational autoencoder on random subsets of the data to determine which settings result in the most stable clustering.

Results

Descriptive statistics

The descriptive statistics are large tables (Table S3-S7) and are thus stored in separate files.

The Recreated DEC model with optimised hyperparameter values

The hyperparameter optimisation of the recreated DEC model showed that the most stable results were achieved with 64 neurons in the hidden layer and 12 neurons in the encoding layer. Recomputing the cluster stability over 1,000 subsets of 90% random patient samples showed that the optimal recreated DEC model achieved an average Jaccard similarity coefficient of 0.547 and sample-wise stability of 71.4%. The cluster- and sample-wise stability metrics are visualised in Fig. S6. Mean cluster-wise stability ranged between 0.385 and 0.646, and most patients had a sample-wise stability ranging between 50%-100%.

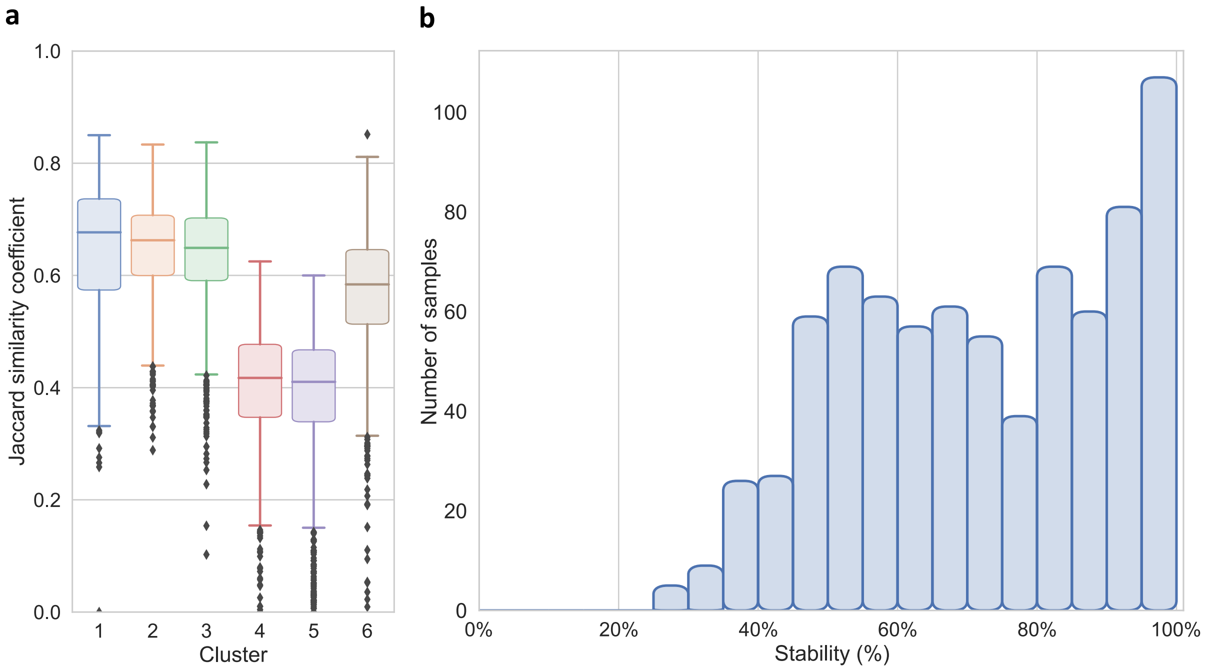

Figure S4. Stability plots of the optimised DEC model on the SICS data set. (A) A box-and-whisker plot of the Jaccard similarity coefficients per cluster. (B) A bar plot of the sample-wise stability, the y-axis indicates the number of samples in each bar, and the x-axis indicates the stability in terms of how often the samples were clustered into their reference cluster.

References

1. Xie, J., Girshick, R. & Farhadi, A. Unsupervised Deep Embedding for Clustering Analysis. 10 (2016).

2. Simidjievski, N. *et al.* Variational Autoencoders for Cancer Data Integration: Design Principles and Computational Practice. *Front. Genet.* **10**, (2019).
