# Supplementary material for: Deep embedded clustering generalisability and adaptation for integrating mixed datatypes: two critical care cohorts
Source: Sci Rep. 2024 Jan 10;14:1045. doi: 10.1038/s41598-024-51699-z (PMC10781731; doi:10.1038/s41598-024-51699-z)
Supplement: Supplementary file 2 — Supplementary Table S3. [file 41598_2024_51699_MOESM2_ESM.docx]

**Table S3. Descriptive statistics of the input and outcome variables for the SICS and MUMC+ datasets.** The first column specifies the variables (in bold), and whether the variables is described by its mean with standard deviation (SD) and the range of values, or if it is a category, the number of patients in each level, and how much percentage of the patients fall in that level. If the variable is missing for some samples, this is indicated by ‘N-miss’, which specifies the number of samples for which this variable was missing. The other columns specify the different datasets, and the combination of both. The last column specifies the p-value. The Chi-square test was used for categorical variables, and the Wilcoxon test for numeric variables.

|  | MUMC+ (N=3894) | SICS (N=787) | Total (N=4681) | p value |
| --- | --- | --- | --- | --- |
| In-ICU mortality |  |  |  | 0.010 |
| Survivor | 3017 (77.5%) | 641 (81.4%) | 3658 (78.1%) |  |
| Non-survivor | 877 (22.5%) | 146 (18.6%) | 1023 (21.9%) |  |
| Length of stay |  |  |  | 0.006 |
| Mean (SD) | 7.748 (11.968) | 5.921 (7.599) | 7.441 (11.371) |  |
| Range | 1.003 - 188.375 | 1.000 - 83.651 | 1.000 - 188.375 |  |
| Apache IV mortality |  |  |  | < 0.001 |
| N-Miss | 776 | 109 | 885 |  |
| Mean (SD) | 0.369 (0.276) | 0.319 (0.255) | 0.360 (0.273) |  |
| Range | 0.002 - 0.996 | 0.001 - 0.981 | 0.001 - 0.996 |  |
| Apache IV score |  |  |  | 0.013 |
| N-Miss | 776 | 109 | 885 |  |
| Mean (SD) | 79.679 (31.131) | 76.242 (28.151) | 79.065 (30.645) |  |
| Range | 9.000 - 217.000 | 17.000 - 216.000 | 9.000 - 217.000 |  |
| SAPS II score |  |  |  | 0.017 |
| Mean (SD) | 45.013 (16.315) | 46.418 (16.288) | 45.249 (16.317) |  |
| Range | 6.000 - 113.000 | 6.000 - 111.000 | 6.000 - 113.000 |  |
| Post-operative |  |  |  | < 0.001 |
| No | 2935 (75.4%) | 520 (66.1%) | 3455 (73.8%) |  |
| Yes | 959 (24.6%) | 267 (33.9%) | 1226 (26.2%) |  |
| Admission diagnosis (Based on APACHE IV) |  |  |  | < 0.001 |
| Cardiovascular | 1267 (32.5%) | 251 (31.9%) | 1518 (32.4%) |  |
| Gastrointestinal | 502 (12.9%) | 108 (13.7%) | 610 (13.0%) |  |
| Genito-urinary | 47 (1.2%) | 13 (1.7%) | 60 (1.3%) |  |
| Haematological | 158 (4.1%) | 10 (1.3%) | 168 (3.6%) |  |
| Metabolic | 108 (2.8%) | 18 (2.3%) | 126 (2.7%) |  |
| Musculoskeletal/skin | 23 (0.6%) | 8 (1.0%) | 31 (0.7%) |  |
| Neurological | 582 (14.9%) | 123 (15.6%) | 705 (15.1%) |  |
| Respiratory | 910 (23.4%) | 160 (20.3%) | 1070 (22.9%) |  |
| Transplant | 11 (0.3%) | 38 (4.8%) | 49 (1.0%) |  |
| Trauma | 226 (5.8%) | 57 (7.2%) | 283 (6.0%) |  |
| Other | 60 (1.5%) | 1 (0.1%) | 61 (1.3%) |  |
| Age |  |  |  | 0.082 |
| N-Miss | 1 | 0 | 1 |  |
| Mean (SD) | 62.781 (15.255) | 62.131 (14.168) | 62.672 (15.078) |  |
| Range | 18.000 - 102.000 | 18.000 - 94.000 | 18.000 - 102.000 |  |
| Gender |  |  |  | 0.774 |
| Female | 1417 (36.4%) | 291 (37.0%) | 1708 (36.5%) |  |
| Male | 2477 (63.6%) | 496 (63.0%) | 2973 (63.5%) |  |
| vasoactive |  |  |  | < 0.001 |
| No | 919 (23.6%) | 403 (51.2%) | 1322 (28.2%) |  |
| Yes | 2975 (76.4%) | 384 (48.8%) | 3359 (71.8%) |  |
| Renal replacement therapy |  |  |  | < 0.001 |
| No | 3510 (90.1%) | 762 (96.8%) | 4272 (91.3%) |  |
| Yes | 384 (9.9%) | 25 (3.2%) | 409 (8.7%) |  |
| NOR |  |  |  | < 0.001 |
| No | 3017 (77.5%) | 413 (52.5%) | 3430 (73.3%) |  |
| Yes | 877 (22.5%) | 374 (47.5%) | 1251 (26.7%) |  |
| ICU readmission |  |  |  | 0.355 |
| 1st readmission | 335 (8.6%) | 78 (9.9%) | 413 (8.8%) |  |
| 2nd readmission | 55 (1.4%) | 10 (1.3%) | 65 (1.4%) |  |
| no readmission | 3471 (89.1%) | 699 (88.8%) | 4170 (89.1%) |  |
| EMV score |  |  |  | < 0.001 |
| N-Miss | 843 | 0 | 843 |  |
| Mean (SD) | 8.574 (5.456) | 11.313 (5.066) | 9.136 (5.490) |  |
| Range | 3.000 - 15.000 | 2.000 - 15.000 | 2.000 - 15.000 |  |
| Temperature centre |  |  |  | < 0.001 |
| N-Miss | 8 | 9 | 17 |  |
| Mean (SD) | 36.249 (1.723) | 36.948 (0.899) | 36.366 (1.635) |  |
| Range | 20.700 - 41.900 | 33.400 - 40.200 | 20.700 - 41.900 |  |
| Admission type |  |  |  | < 0.001 |
| Acute surgery | 853 (21.9%) | 244 (31.0%) | 1097 (23.4%) |  |
| Medical | 3041 (78.1%) | 514 (65.3%) | 3555 (75.9%) |  |
| ALAT mean |  |  |  | 0.002 |
| N-Miss | 21 | 8 | 29 |  |
| Mean (SD) | 125.446 (388.582) | 122.331 (337.898) | 124.924 (380.533) |  |
| Range | 5.000 - 8185.400 | 5.000 - 5141.200 | 5.000 - 8185.400 |  |
| ALAT variance |  |  |  | 0.007 |
| N-Miss | 21 | 8 | 29 |  |
| Mean (SD) | 62.650 (256.702) | 60.733 (266.928) | 62.329 (258.414) |  |
| Range | 0.000 - 4718.108 | 0.000 - 5065.483 | 0.000 - 5065.483 |  |
| ASAT mean |  |  |  | 0.644 |
| N-Miss | 21 | 8 | 29 |  |
| Mean (SD) | 190.240 (656.773) | 186.432 (570.866) | 189.603 (643.132) |  |
| Range | 6.667 - 12116.750 | 10.333 - 9648.600 | 6.667 - 12116.750 |  |
| ASAT variance |  |  |  | 0.306 |
| N-Miss | 21 | 8 | 29 |  |
| Mean (SD) | 127.807 (555.795) | 124.318 (563.614) | 127.223 (557.053) |  |
| Range | 0.000 - 8784.455 | 0.000 - 10628.866 | 0.000 - 10628.866 |  |
| Albumin mean |  |  |  | < 0.001 |
| N-Miss | 607 | 20 | 627 |  |
| Mean (SD) | 23.238 (7.057) | 29.662 (6.384) | 24.453 (7.377) |  |
| Range | 4.200 - 48.300 | 12.938 - 46.500 | 4.200 - 48.300 |  |
| Albumin variance |  |  |  | < 0.001 |
| N-Miss | 607 | 20 | 627 |  |
| Mean (SD) | 1.627 (1.810) | 2.150 (1.842) | 1.726 (1.828) |  |
| Range | 0.000 - 14.114 | 0.000 - 10.873 | 0.000 - 14.114 |  |
| ALP mean |  |  |  | < 0.001 |
| N-Miss | 161 | 13 | 174 |  |
| Mean (SD) | 130.024 (131.422) | 102.228 (158.102) | 125.250 (136.759) |  |
| Range | 16.500 - 2799.667 | 18.333 - 3860.000 | 16.500 - 3860.000 |  |
| ALP variance |  |  |  | < 0.001 |
| N-Miss | 161 | 13 | 174 |  |
| Mean (SD) | 28.128 (57.270) | 18.410 (38.850) | 26.459 (54.670) |  |
| Range | 0.000 - 909.332 | 0.000 - 643.456 | 0.000 - 909.332 |  |
| Bilirubin (total) mean |  |  |  | 0.087 |
| N-Miss | 193 | 14 | 207 |  |
| Mean (SD) | 17.568 (34.351) | 17.179 (41.061) | 17.501 (35.596) |  |
| Range | 2.100 - 635.629 | 3.000 - 554.714 | 2.100 - 635.629 |  |
| Bilirubin (total) variance |  |  |  | 0.320 |
| N-Miss | 193 | 14 | 207 |  |
| Mean (SD) | 4.146 (9.669) | 4.297 (13.044) | 4.172 (10.330) |  |
| Range | 0.000 - 163.082 | 0.000 - 202.583 | 0.000 - 202.583 |  |
| CK mean |  |  |  | < 0.001 |
| N-Miss | 313 | 0 | 313 |  |
| Mean (SD) | 762.107 (2603.716) | 905.992 (3878.614) | 788.031 (2875.466) |  |
| Range | 7.000 - 85000.667 | 7.000 - 68697.167 | 7.000 - 85000.667 |  |
| CK variance |  |  |  | < 0.001 |
| N-Miss | 313 | 0 | 313 |  |
| Mean (SD) | 405.321 (1358.918) | 474.350 (2006.426) | 417.759 (1496.377) |  |
| Range | 0.000 - 31656.416 | 0.000 - 39751.232 | 0.000 - 39751.232 |  |
| CRP mean |  |  |  | < 0.001 |
| N-Miss | 17 | 0 | 17 |  |
| Mean (SD) | 115.081 (85.144) | 94.453 (85.859) | 111.600 (85.605) |  |
| Range | 1.000 - 598.333 | 0.300 - 444.600 | 0.300 - 598.333 |  |
| CRP variance |  |  |  | < 0.001 |
| N-Miss | 17 | 0 | 17 |  |
| Mean (SD) | 50.361 (38.307) | 43.855 (35.769) | 49.263 (37.965) |  |
| Range | 0.000 - 225.975 | 0.000 - 194.114 | 0.000 - 225.975 |  |
| Calcium mean |  |  |  | < 0.001 |
| N-Miss | 387 | 17 | 404 |  |
| Mean (SD) | 2.018 (0.179) | 2.050 (0.159) | 2.024 (0.176) |  |
| Range | 1.247 - 2.844 | 1.584 - 2.933 | 1.247 - 2.933 |  |
| Calcium variance |  |  |  | 0.415 |
| N-Miss | 387 | 17 | 404 |  |
| Mean (SD) | 0.072 (0.068) | 0.072 (0.062) | 0.072 (0.067) |  |
| Range | 0.000 - 0.579 | 0.000 - 0.479 | 0.000 - 0.579 |  |
| Chloride mean |  |  |  | < 0.001 |
| N-Miss | 454 | 1 | 455 |  |
| Mean (SD) | 107.720 (6.310) | 103.839 (5.397) | 106.998 (6.332) |  |
| Range | 73.000 - 138.000 | 72.000 - 125.400 | 72.000 - 138.000 |  |
| Chloride variance |  |  |  | 0.005 |
| N-Miss | 454 | 1 | 455 |  |
| Mean (SD) | 2.480 (2.352) | 2.471 (1.718) | 2.478 (2.247) |  |
| Range | 0.000 - 18.707 | 0.000 - 12.021 | 0.000 - 18.707 |  |
| Protein (total) mean |  |  |  | < 0.001 |
| N-Miss | 1518 | 18 | 1536 |  |
| Mean (SD) | 51.667 (9.477) | 54.274 (8.899) | 52.304 (9.404) |  |
| Range | 12.500 - 100.912 | 28.444 - 80.000 | 12.500 - 100.912 |  |
| Protein (total) variance |  |  |  | < 0.001 |
| N-Miss | 1518 | 18 | 1536 |  |
| Mean (SD) | 1.765 (2.611) | 3.298 (3.297) | 2.140 (2.870) |  |
| Range | 0.000 - 18.528 | 0.000 - 24.366 | 0.000 - 24.366 |  |
| Fibrinogen mean |  |  |  | < 0.001 |
| N-Miss | 1194 | 79 | 1273 |  |
| Mean (SD) | 4.062 (1.864) | 3.533 (1.816) | 3.952 (1.866) |  |
| Range | 0.400 - 11.500 | 0.450 - 9.967 | 0.400 - 11.500 |  |
| Fibrinogen variance |  |  |  | 0.005 |
| N-Miss | 1194 | 79 | 1273 |  |
| Mean (SD) | 0.378 (0.591) | 0.368 (0.547) | 0.376 (0.583) |  |
| Range | 0.000 - 3.755 | 0.000 - 3.333 | 0.000 - 3.755 |  |
| Phosphate mean |  |  |  | < 0.001 |
| N-Miss | 158 | 1 | 159 |  |
| Mean (SD) | 1.150 (0.390) | 1.088 (0.353) | 1.139 (0.385) |  |
| Range | 0.270 - 3.870 | 0.305 - 3.447 | 0.270 - 3.870 |  |
| Phosphate variance |  |  |  | 0.152 |
| N-Miss | 158 | 1 | 159 |  |
| Mean (SD) | 0.234 (0.197) | 0.233 (0.165) | 0.234 (0.192) |  |
| Range | 0.000 - 1.808 | 0.000 - 1.319 | 0.000 - 1.808 |  |
| Gamma-GT mean |  |  |  | < 0.001 |
| N-Miss | 104 | 13 | 117 |  |
| Mean (SD) | 116.514 (174.898) | 94.985 (140.567) | 112.862 (169.745) |  |
| Range | 5.500 - 4794.500 | 6.333 - 1608.800 | 5.500 - 4794.500 |  |
| Gamma-GT variance |  |  |  | < 0.001 |
| N-Miss | 104 | 13 | 117 |  |
| Mean (SD) | 35.414 (71.852) | 22.815 (46.280) | 33.277 (68.353) |  |
| Range | 0.000 - 1186.909 | 0.000 - 571.626 | 0.000 - 1186.909 |  |
| Haemoglobin mean |  |  |  | < 0.001 |
| Mean (SD) | 6.525 (1.234) | 6.736 (1.309) | 6.560 (1.249) |  |
| Range | 4.050 - 11.850 | 4.067 - 10.850 | 4.050 - 11.850 |  |
| Haemoglobin variance |  |  |  | 0.310 |
| Mean (SD) | 0.602 (0.328) | 0.635 (0.383) | 0.608 (0.338) |  |
| Range | 0.000 - 2.444 | 0.000 - 2.173 | 0.000 - 2.444 |  |
| Haematocrit mean |  |  |  | < 0.001 |
| N-Miss | 65 | 0 | 65 |  |
| Mean (SD) | 0.323 (0.060) | 0.332 (0.063) | 0.324 (0.061) |  |
| Range | 0.190 - 0.567 | 0.197 - 0.515 | 0.190 - 0.567 |  |
| Haematocrit variance |  |  |  | < 0.001 |
| N-Miss | 65 | 0 | 65 |  |
| Mean (SD) | 0.026 (0.017) | 0.030 (0.019) | 0.027 (0.018) |  |
| Range | 0.000 - 0.120 | 0.000 - 0.111 | 0.000 - 0.120 |  |
| Potassium mean |  |  |  | < 0.001 |
| N-Miss | 532 | 158 | 690 |  |
| Mean (SD) | 4.279 (0.605) | 4.207 (0.861) | 4.268 (0.653) |  |
| Range | 1.880 - 9.360 | 2.300 - 13.000 | 1.880 - 13.000 |  |
| Potassium variance |  |  |  | < 0.001 |
| N-Miss | 532 | 158 | 690 |  |
| Mean (SD) | 0.336 (0.307) | 0.071 (0.201) | 0.294 (0.308) |  |
| Range | 0.000 - 2.950 | 0.000 - 1.850 | 0.000 - 2.950 |  |
| Creatinine mean |  |  |  | < 0.001 |
| Mean (SD) | 123.417 (107.379) | 109.734 (97.539) | 121.117 (105.903) |  |
| Range | 5.000 - 1557.833 | 5.500 - 1146.750 | 5.000 - 1557.833 |  |
| Creatinine variance |  |  |  | < 0.001 |
| Mean (SD) | 24.346 (35.178) | 19.438 (39.442) | 23.521 (35.972) |  |
| Range | 0.000 - 488.499 | 0.000 - 863.565 | 0.000 - 863.565 |  |
| LDH mean |  |  |  | 0.300 |
| N-Miss | 108 | 0 | 108 |  |
| Mean (SD) | 442.081 (665.884) | 416.123 (513.871) | 437.614 (642.316) |  |
| Range | 68.500 - 13633.400 | 84.000 - 6250.200 | 68.500 - 13633.400 |  |
| LDH variance |  |  |  | < 0.001 |
| N-Miss | 108 | 0 | 108 |  |
| Mean (SD) | 147.644 (518.665) | 146.330 (414.752) | 147.418 (502.274) |  |
| Range | 0.000 - 7313.211 | 0.000 - 5767.509 | 0.000 - 7313.211 |  |
| Leukocytes mean |  |  |  | < 0.001 |
| N-Miss | 23 | 0 | 23 |  |
| Mean (SD) | 12.990 (9.672) | 14.009 (8.969) | 13.162 (9.564) |  |
| Range | 0.100 - 417.750 | 0.000 - 170.000 | 0.000 - 417.750 |  |
| Leukocytes variance |  |  |  | 0.221 |
| N-Miss | 23 | 0 | 23 |  |
| Mean (SD) | 3.223 (3.614) | 3.284 (3.885) | 3.233 (3.661) |  |
| Range | 0.000 - 102.892 | 0.000 - 78.885 | 0.000 - 102.892 |  |
| Magnesium mean |  |  |  | 0.167 |
| N-Miss | 456 | 0 | 456 |  |
| Mean (SD) | 0.830 (0.145) | 0.847 (0.163) | 0.833 (0.149) |  |
| Range | 0.343 - 2.686 | 0.500 - 2.115 | 0.343 - 2.686 |  |
| Magnesium variance |  |  |  | < 0.001 |
| N-Miss | 456 | 0 | 456 |  |
| Mean (SD) | 0.067 (0.065) | 0.107 (0.087) | 0.075 (0.071) |  |
| Range | 0.000 - 0.871 | 0.000 - 0.586 | 0.000 - 0.871 |  |
| Sodium mean |  |  |  | < 0.001 |
| N-Miss | 544 | 0 | 544 |  |
| Mean (SD) | 140.485 (5.208) | 139.299 (4.681) | 140.260 (5.132) |  |
| Range | 108.500 - 169.750 | 110.250 - 155.375 | 108.500 - 169.750 |  |
| Sodium variance |  |  |  | < 0.001 |
| N-Miss | 544 | 0 | 544 |  |
| Mean (SD) | 2.109 (2.041) | 2.313 (1.520) | 2.148 (1.954) |  |
| Range | 0.000 - 17.913 | 0.000 - 10.852 | 0.000 - 17.913 |  |
| Thrombocytes mean |  |  |  | 0.386 |
| N-Miss | 2 | 0 | 2 |  |
| Mean (SD) | 226.759 (122.138) | 227.281 (110.063) | 226.847 (120.181) |  |
| Range | 12.545 - 1580.000 | 16.900 - 868.812 | 12.545 - 1580.000 |  |
| Thrombocytes variance |  |  |  | 0.017 |
| N-Miss | 2 | 0 | 2 |  |
| Mean (SD) | 48.900 (49.696) | 42.618 (42.919) | 47.843 (48.675) |  |
| Range | 0.000 - 571.822 | 0.000 - 507.755 | 0.000 - 571.822 |  |
| Urea mean |  |  |  | 0.029 |
| N-Miss | 2 | 0 | 2 |  |
| Mean (SD) | 10.374 (7.460) | 9.560 (6.607) | 10.237 (7.329) |  |
| Range | 1.000 - 72.300 | 0.867 - 47.650 | 0.867 - 72.300 |  |
| Urea variance |  |  |  | 0.002 |
| N-Miss | 2 | 0 | 2 |  |
| Mean (SD) | 2.268 (2.616) | 1.899 (2.210) | 2.206 (2.556) |  |
| Range | 0.000 - 24.404 | 0.000 - 18.464 | 0.000 - 24.404 |  |
| BMI |  |  |  | 0.005 |
| N-Miss | 384 | 0 | 384 |  |
| Mean (SD) | 26.279 (5.488) | 26.768 (5.242) | 26.369 (5.447) |  |
| Range | 11.400 - 82.700 | 13.870 - 57.370 | 11.400 - 82.700 |  |
| Previous ICU admission |  |  |  | 0.789 |
| No | 3471 (89.1%) | 699 (88.8%) | 4170 (89.1%) |  |
| Yes | 423 (10.9%) | 88 (11.2%) | 511 (10.9%) |  |
| Systolic blood pressure |  |  |  | < 0.001 |
| N-Miss | 1 | 2 | 3 |  |
| Mean (SD) | 125.354 (33.556) | 119.145 (24.925) | 124.312 (32.350) |  |
| Range | 0.000 - 313.000 | 64.000 - 212.000 | 0.000 - 313.000 |  |
| Diastolic blood pressure |  |  |  | < 0.001 |
| N-Miss | 1 | 2 | 3 |  |
| Mean (SD) | 66.177 (20.179) | 61.270 (12.209) | 65.354 (19.162) |  |
| Range | 0.000 - 196.000 | 26.000 - 147.000 | 0.000 - 196.000 |  |
| Mean arterial pressure |  |  |  | < 0.001 |
| N-Miss | 1 | 3 | 4 |  |
| Mean (SD) | 66.177 (20.179) | 79.429 (15.055) | 68.399 (20.034) |  |
| Range | 0.000 - 196.000 | 32.500 - 168.000 | 0.000 - 196.000 |  |
| Atrial fibrillation |  |  |  | 0.006 |
| No | 3502 (89.9%) | 733 (93.1%) | 4235 (90.5%) |  |
| Yes | 392 (10.1%) | 54 (6.9%) | 446 (9.5%) |  |
| Heart rate at admission |  |  |  | 0.830 |
| N-Miss | 0 | 6 | 6 |  |
| Mean (SD) | 95.379 (25.751) | 95.006 (24.319) | 95.316 (25.515) |  |
| Range | 1.000 - 250.000 | 40.000 - 207.000 | 1.000 - 250.000 |  |
| Urine output in previous 6 hours |  |  |  | < 0.001 |
| N-Miss | 143 | 15 | 158 |  |
| Mean (SD) | 0.551 (0.582) | 0.906 (0.796) | 0.612 (0.638) |  |
| Range | 0.000 - 12.000 | 0.000 - 5.689 | 0.000 - 12.000 |  |
| Central venous pressure |  |  |  | < 0.001 |
| No | 3361 (86.3%) | 621 (78.9%) | 3982 (85.1%) |  |
| Yes | 533 (13.7%) | 166 (21.1%) | 699 (14.9%) |  |
| Worsened respiratory condition |  |  |  | < 0.001 |
| No | 2734 (70.2%) | 690 (87.7%) | 3424 (73.1%) |  |
| Yes | 1160 (29.8%) | 97 (12.3%) | 1257 (26.9%) |  |
| Tidal volume |  |  |  | < 0.001 |
| N-Miss | 1065 | 330 | 1395 |  |
| Mean (SD) | 482.321 (161.387) | 511.314 (119.798) | 486.353 (156.573) |  |
| Range | 0.000 - 1919.000 | 0.500 - 1249.000 | 0.000 - 1919.000 |  |
| Respiratory rate |  |  |  | < 0.001 |
| N-Miss | 1412 | 330 | 1742 |  |
| Mean (SD) | 17.082 (3.763) | 15.795 (4.627) | 16.882 (3.936) |  |
| Range | 4.000 - 70.000 | 2.600 - 36.000 | 2.600 - 70.000 |  |
| Positive end-expiratory pressure |  |  |  | 0.286 |
| N-Miss | 14 | 324 | 338 |  |
| Mean (SD) | 7.188 (2.525) | 7.261 (2.336) | 7.196 (2.506) |  |
| Range | 0.000 - 24.000 | 2.000 - 15.000 | 0.000 - 24.000 |  |
| Mechanical ventilation after 24h |  |  |  | < 0.001 |
| No | 1781 (45.7%) | 205 (26.0%) | 1986 (42.4%) |  |
| Yes | 2113 (54.3%) | 582 (74.0%) | 2695 (57.6%) |  |
| Mechanical ventilation at admission |  |  |  | < 0.001 |
| No | 2568 (65.9%) | 302 (38.4%) | 2870 (61.3%) |  |
| Yes | 1326 (34.1%) | 485 (61.6%) | 1811 (38.7%) |  |
| Respiratory rate |  |  |  | < 0.001 |
| N-Miss | 0 | 3 | 3 |  |
| Mean (SD) | 20.879 (8.364) | 18.089 (5.678) | 20.412 (8.045) |  |
| Range | 0.000 - 120.000 | 5.000 - 58.000 | 0.000 - 120.000 |  |
| FiO2 low |  |  |  | < 0.001 |
| N-Miss | 787 | 6 | 793 |  |
| Mean (SD) | 31.709 (9.710) | 47.015 (22.760) | 34.784 (14.728) |  |
| Range | 21.000 - 100.000 | 21.000 - 100.000 | 21.000 - 100.000 |  |
| Myocardial infarction (history) |  |  |  | 0.055 |
| No | 3496 (89.8%) | 724 (92.0%) | 4220 (90.2%) |  |
| Yes | 398 (10.2%) | 63 (8.0%) | 461 (9.8%) |  |
| Diabetes (history) |  |  |  | < 0.001 |
| No | 3433 (88.2%) | 626 (79.5%) | 4059 (86.7%) |  |
| Yes | 461 (11.8%) | 161 (20.5%) | 622 (13.3%) |  |
| Cardiovascular disease (history) |  |  |  | 0.285 |
| No | 3661 (94.0%) | 748 (95.0%) | 4409 (94.2%) |  |
| Yes | 233 (6.0%) | 39 (5.0%) | 272 (5.8%) |  |
| Chronic Obstructive pulmonary disease (history) |  |  |  | 0.216 |
| No | 3471 (89.1%) | 689 (87.5%) | 4160 (88.9%) |  |
| Yes | 423 (10.9%) | 98 (12.5%) | 521 (11.1%) |  |
| Respiratory insufficiency (history) |  |  |  | 0.256 |
| No | 3733 (95.9%) | 747 (94.9%) | 4480 (95.7%) |  |
| Yes | 161 (4.1%) | 40 (5.1%) | 201 (4.3%) |  |
| Chronic kidney disease (history) |  |  |  | < 0.001 |
| No | 3894 (100.0%) | 733 (93.1%) | 4627 (98.8%) |  |
| Yes | 0 (0.0%) | 54 (6.9%) | 54 (1.2%) |  |
| Dialysis (history) |  |  |  | 0.193 |
| No | 3817 (98.0%) | 777 (98.7%) | 4594 (98.1%) |  |
| Yes | 77 (2.0%) | 10 (1.3%) | 87 (1.9%) |  |
| Cirrhosis (history) |  |  |  | 0.207 |
| No | 3803 (97.7%) | 762 (96.8%) | 4565 (97.5%) |  |
| Yes | 91 (2.3%) | 25 (3.2%) | 116 (2.5%) |  |
| Metastatic disease (history) |  |  |  | 0.040 |
| No | 3690 (94.8%) | 760 (96.6%) | 4450 (95.1%) |  |
| Yes | 204 (5.2%) | 27 (3.4%) | 231 (4.9%) |  |
| Haematological malignancy (history) |  |  |  | 0.033 |
| No | 3668 (94.2%) | 756 (96.1%) | 4424 (94.5%) |  |
| Yes | 226 (5.8%) | 31 (3.9%) | 257 (5.5%) |  |
| Immune insufficiency (history) |  |  |  | < 0.001 |
| No | 3550 (91.2%) | 676 (85.9%) | 4226 (90.3%) |  |
| Yes | 344 (8.8%) | 111 (14.1%) | 455 (9.7%) |  |
