# Supplementary material for: Deep embedded clustering generalisability and adaptation for integrating mixed datatypes: two critical care cohorts
Source: Sci Rep. 2024 Jan 10;14:1045. doi: 10.1038/s41598-024-51699-z (PMC10781731; doi:10.1038/s41598-024-51699-z)
Supplement: Supplementary file 3 — Supplementary Table S4. [file 41598_2024_51699_MOESM3_ESM.docx]

**Table S4. Descriptive statistics of the input and outcome variables for the recreated DEC clusters on the SICS dataset.** The first column specifies the variables (in bold), and whether the variables is described by its mean with standard deviation (SD) and the range of values, or if it is a category, the number of patients in each level, and how much percentage of the patients fall in that level. If the variable is missing for some samples, this is indicated by ‘N-miss’, which specifies the number of samples for which this variable was missing. The other columns specify the different clusters, and complete dataset. The last column specifies the p-value. The Chi-square test was used for categorical variables, and the Kruskal-Wallis Rank Test for numeric variables.

|  | Cluster 1 (N=247) | Cluster 2 (N=22) | Cluster 3 (N=111) | Cluster 4 (N=138) | Cluster 5 (N=172) | Cluster 6 (N=97) | Total (N=787) | p value |
| --- | --- | --- | --- | --- | --- | --- | --- | --- |
| In-ICU mortality |  |  |  |  |  |  |  | < 0.001 |
| Survivor | 207 (83.8%) | 10 (45.5%) | 94 (84.7%) | 112 (81.2%) | 152 (88.4%) | 66 (68.0%) | 641 (81.4%) |  |
| Non-survivor | 40 (16.2%) | 12 (54.5%) | 17 (15.3%) | 26 (18.8%) | 20 (11.6%) | 31 (32.0%) | 146 (18.6%) |  |
| Length of stay |  |  |  |  |  |  |  | < 0.001 |
| Mean (SD) | 5.194 (6.872) | 8.974 (7.709) | 4.620 (6.677) | 6.177 (7.012) | 5.671 (8.764) | 8.650 (8.221) | 5.921 (7.599) |  |
| Range | 1.083 - 83.651 | 1.549 - 32.369 | 1.012 - 50.140 | 1.022 - 42.450 | 1.035 - 70.924 | 1.000 - 33.719 | 1.000 - 83.651 |  |
| Apache IV mortality |  |  |  |  |  |  |  | < 0.001 |
| N-Miss | 55 | 1 | 9 | 22 | 12 | 10 | 109 |  |
| Mean (SD) | 0.305 (0.234) | 0.552 (0.302) | 0.267 (0.207) | 0.355 (0.298) | 0.274 (0.221) | 0.390 (0.287) | 0.319 (0.255) |  |
| Range | 0.001 - 0.890 | 0.045 - 0.975 | 0.015 - 0.944 | 0.004 - 0.944 | 0.006 - 0.848 | 0.026 - 0.981 | 0.001 - 0.981 |  |
| Apache IV score |  |  |  |  |  |  |  | < 0.001 |
| N-Miss | 55 | 1 | 9 | 22 | 12 | 10 | 109 |  |
| Mean (SD) | 74.047 (23.696) | 110.524 (37.651) | 72.647 (21.799) | 81.155 (27.681) | 63.712 (23.768) | 93.517 (33.087) | 76.242 (28.151) |  |
| Range | 21.000 - 140.000 | 41.000 - 194.000 | 31.000 - 157.000 | 32.000 - 151.000 | 17.000 - 121.000 | 39.000 - 216.000 | 17.000 - 216.000 |  |
| SAPS II score |  |  |  |  |  |  |  | < 0.001 |
| Mean (SD) | 43.773 (14.381) | 65.455 (19.415) | 40.297 (13.088) | 51.536 (15.563) | 42.919 (15.485) | 54.763 (17.789) | 46.418 (16.288) |  |
| Range | 6.000 - 86.000 | 37.000 - 111.000 | 6.000 - 69.000 | 13.000 - 94.000 | 14.000 - 79.000 | 20.000 - 101.000 | 6.000 - 111.000 |  |
| Postoperative (based on APACHE IV) |  |  |  |  |  |  |  | < 0.001 |
| No | 200 (81.0%) | 16 (72.7%) | 87 (78.4%) | 68 (49.3%) | 90 (52.3%) | 59 (60.8%) | 520 (66.1%) |  |
| Yes | 47 (19.0%) | 6 (27.3%) | 24 (21.6%) | 70 (50.7%) | 82 (47.7%) | 38 (39.2%) | 267 (33.9%) |  |
| Admission diagnosis (based on APACHE IV) |  |  |  |  |  |  |  | < 0.001 |
|  | 0 (0.0%) | 0 (0.0%) | 1 (0.9%) | 0 (0.0%) | 0 (0.0%) | 0 (0.0%) | 1 (0.1%) |  |
| Cardiovascular | 77 (31.2%) | 8 (36.4%) | 23 (20.7%) | 75 (54.3%) | 44 (25.6%) | 24 (24.7%) | 251 (31.9%) |  |
| Gastrointestinal | 34 (13.8%) | 5 (22.7%) | 22 (19.8%) | 15 (10.9%) | 4 (2.3%) | 28 (28.9%) | 108 (13.7%) |  |
| Genito-urinary | 2 (0.8%) | 1 (4.5%) | 3 (2.7%) | 3 (2.2%) | 0 (0.0%) | 4 (4.1%) | 13 (1.7%) |  |
| Haematological | 1 (0.4%) | 1 (4.5%) | 3 (2.7%) | 0 (0.0%) | 0 (0.0%) | 5 (5.2%) | 10 (1.3%) |  |
| Metabolic | 4 (1.6%) | 0 (0.0%) | 8 (7.2%) | 1 (0.7%) | 2 (1.2%) | 3 (3.1%) | 18 (2.3%) |  |
| Musculoskeletal/skin | 1 (0.4%) | 0 (0.0%) | 3 (2.7%) | 0 (0.0%) | 1 (0.6%) | 3 (3.1%) | 8 (1.0%) |  |
| Neurological | 29 (11.7%) | 0 (0.0%) | 5 (4.5%) | 9 (6.5%) | 77 (44.8%) | 3 (3.1%) | 123 (15.6%) |  |
| Respiratory | 79 (32.0%) | 4 (18.2%) | 39 (35.1%) | 12 (8.7%) | 12 (7.0%) | 14 (14.4%) | 160 (20.3%) |  |
| Transplant | 4 (1.6%) | 2 (9.1%) | 2 (1.8%) | 15 (10.9%) | 4 (2.3%) | 11 (11.3%) | 38 (4.8%) |  |
| Trauma | 16 (6.5%) | 1 (4.5%) | 2 (1.8%) | 8 (5.8%) | 28 (16.3%) | 2 (2.1%) | 57 (7.2%) |  |
| Age |  |  |  |  |  |  |  | < 0.001 |
| Mean (SD) | 66.676 (12.154) | 55.273 (17.002) | 61.306 (13.662) | 62.616 (13.258) | 58.308 (15.163) | 59.144 (15.073) | 62.131 (14.168) |  |
| Range | 23.000 - 94.000 | 21.000 - 82.000 | 18.000 - 84.000 | 25.000 - 89.000 | 20.000 - 89.000 | 21.000 - 87.000 | 18.000 - 94.000 |  |
| Gender |  |  |  |  |  |  |  | 0.181 |
| Female | 78 (31.6%) | 7 (31.8%) | 44 (39.6%) | 50 (36.2%) | 76 (44.2%) | 36 (37.1%) | 291 (37.0%) |  |
| Male | 169 (68.4%) | 15 (68.2%) | 67 (60.4%) | 88 (63.8%) | 96 (55.8%) | 61 (62.9%) | 496 (63.0%) |  |
| Vasoactive |  |  |  |  |  |  |  | < 0.001 |
| No | 150 (60.7%) | 4 (18.2%) | 68 (61.3%) | 36 (26.1%) | 121 (70.3%) | 24 (24.7%) | 403 (51.2%) |  |
| Yes | 97 (39.3%) | 18 (81.8%) | 43 (38.7%) | 102 (73.9%) | 51 (29.7%) | 73 (75.3%) | 384 (48.8%) |  |
| Renal replacement therapy |  |  |  |  |  |  |  | 0.002 |
| No | 243 (98.4%) | 19 (86.4%) | 104 (93.7%) | 135 (97.8%) | 171 (99.4%) | 90 (92.8%) | 762 (96.8%) |  |
| Yes | 4 (1.6%) | 3 (13.6%) | 7 (6.3%) | 3 (2.2%) | 1 (0.6%) | 7 (7.2%) | 25 (3.2%) |  |
| NOR |  |  |  |  |  |  |  | < 0.001 |
| No | 153 (61.9%) | 4 (18.2%) | 69 (62.2%) | 40 (29.0%) | 123 (71.5%) | 24 (24.7%) | 413 (52.5%) |  |
| Yes | 94 (38.1%) | 18 (81.8%) | 42 (37.8%) | 98 (71.0%) | 49 (28.5%) | 73 (75.3%) | 374 (47.5%) |  |
| ICU readmission |  |  |  |  |  |  |  | < 0.001 |
| 1st readmission | 47 (19.0%) | 1 (4.5%) | 6 (5.4%) | 11 (8.0%) | 8 (4.7%) | 5 (5.2%) | 78 (9.9%) |  |
| 2nd readmission | 7 (2.8%) | 0 (0.0%) | 0 (0.0%) | 0 (0.0%) | 0 (0.0%) | 3 (3.1%) | 10 (1.3%) |  |
| no readmission | 193 (78.1%) | 21 (95.5%) | 105 (94.6%) | 127 (92.0%) | 164 (95.3%) | 89 (91.8%) | 699 (88.8%) |  |
| EMV score |  |  |  |  |  |  |  | < 0.001 |
| Mean (SD) | 12.053 (4.553) | 11.182 (5.721) | 14.108 (2.661) | 10.833 (5.420) | 8.180 (5.213) | 12.495 (4.648) | 11.313 (5.066) |  |
| Range | 3.000 - 15.000 | 3.000 - 15.000 | 3.000 - 15.000 | 3.000 - 15.000 | 2.000 - 15.000 | 3.000 - 15.000 | 2.000 - 15.000 |  |
| Temperature center |  |  |  |  |  |  |  | 0.065 |
| N-Miss | 1 | 0 | 1 | 1 | 5 | 1 | 9 |  |
| Mean (SD) | 37.032 (0.847) | 37.132 (1.628) | 36.994 (0.778) | 36.745 (0.935) | 36.943 (0.799) | 36.938 (1.012) | 36.948 (0.899) |  |
| Range | 34.000 - 39.900 | 35.000 - 40.200 | 35.000 - 39.500 | 33.400 - 39.400 | 34.900 - 38.700 | 34.800 - 40.100 | 33.400 - 40.200 |  |
| Admission type |  |  |  |  |  |  |  | < 0.001 |
| Acute surgery | 47 (19.0%) | 7 (31.8%) | 17 (15.3%) | 64 (46.4%) | 78 (45.3%) | 31 (32.0%) | 244 (31.0%) |  |
| Medical | 196 (79.4%) | 15 (68.2%) | 89 (80.2%) | 66 (47.8%) | 90 (52.3%) | 58 (59.8%) | 514 (65.3%) |  |
| Planned surgery | 4 (1.6%) | 0 (0.0%) | 5 (4.5%) | 8 (5.8%) | 4 (2.3%) | 8 (8.2%) | 29 (3.7%) |  |
| ALAT mean |  |  |  |  |  |  |  | < 0.001 |
| N-Miss | 1 | 0 | 5 | 0 | 2 | 0 | 8 |  |
| Mean (SD) | 87.327 (179.088) | 625.617 (1134.677) | 40.711 (42.491) | 124.459 (296.795) | 45.653 (58.573) | 317.503 (557.341) | 122.331 (337.898) |  |
| Range | 5.667 - 1463.333 | 5.667 - 5141.200 | 6.333 - 292.273 | 5.000 - 2608.250 | 5.500 - 442.231 | 5.500 - 3104.833 | 5.000 - 5141.200 |  |
| ALAT variance |  |  |  |  |  |  |  | < 0.001 |
| N-Miss | 1 | 0 | 5 | 0 | 2 | 0 | 8 |  |
| Mean (SD) | 28.475 (78.251) | 462.471 (1079.865) | 10.101 (31.784) | 52.794 (155.700) | 10.231 (18.815) | 206.558 (449.237) | 60.733 (266.928) |  |
| Range | 0.000 - 674.000 | 0.471 - 5065.483 | 0.000 - 265.654 | 0.000 - 1359.360 | 0.000 - 122.204 | 0.000 - 2876.831 | 0.000 - 5065.483 |  |
| ASAT mean |  |  |  |  |  |  |  | < 0.001 |
| N-Miss | 1 | 0 | 5 | 0 | 2 | 0 | 8 |  |
| Mean (SD) | 100.718 (189.431) | 1307.751 (2244.464) | 56.258 (78.226) | 170.771 (303.595) | 66.088 (93.896) | 524.937 (909.004) | 186.432 (570.866) |  |
| Range | 12.000 - 1579.167 | 18.667 - 9648.600 | 10.333 - 656.091 | 17.000 - 2811.375 | 10.818 - 710.154 | 10.500 - 4742.143 | 10.333 - 9648.600 |  |
| ASAT variance |  |  |  |  |  |  |  | < 0.001 |
| N-Miss | 1 | 0 | 5 | 0 | 2 | 0 | 8 |  |
| Mean (SD) | 45.601 (138.086) | 1177.248 (2382.132) | 18.754 (69.915) | 89.665 (208.860) | 21.147 (46.545) | 430.614 (902.976) | 124.318 (563.614) |  |
| Range | 0.000 - 1462.999 | 2.867 - 10628.866 | 0.000 - 643.292 | 0.000 - 1881.675 | 0.000 - 370.640 | 0.000 - 4920.835 | 0.000 - 10628.866 |  |
| Albumin mean |  |  |  |  |  |  |  | < 0.001 |
| N-Miss | 6 | 0 | 7 | 1 | 6 | 0 | 20 |  |
| Mean (SD) | 31.612 (5.642) | 25.027 (6.251) | 26.272 (5.565) | 28.035 (5.065) | 34.382 (5.054) | 23.727 (4.338) | 29.662 (6.384) |  |
| Range | 18.000 - 46.500 | 16.500 - 41.333 | 12.938 - 40.000 | 16.765 - 42.000 | 19.833 - 46.000 | 13.222 - 34.500 | 12.938 - 46.500 |  |
| Albumin variance |  |  |  |  |  |  |  | < 0.001 |
| N-Miss | 6 | 0 | 7 | 1 | 6 | 0 | 20 |  |
| Mean (SD) | 2.262 (1.700) | 2.539 (1.188) | 1.395 (1.140) | 3.041 (2.299) | 1.340 (1.586) | 2.724 (1.743) | 2.150 (1.842) |  |
| Range | 0.000 - 9.549 | 0.756 - 5.573 | 0.000 - 4.497 | 0.000 - 10.873 | 0.000 - 8.500 | 0.000 - 7.789 | 0.000 - 10.873 |  |
| ALP mean |  |  |  |  |  |  |  | < 0.001 |
| N-Miss | 3 | 0 | 6 | 0 | 4 | 0 | 13 |  |
| Mean (SD) | 82.522 (49.148) | 409.008 (796.929) | 122.440 (84.170) | 72.673 (35.229) | 68.599 (22.072) | 160.634 (127.489) | 102.228 (158.102) |  |
| Range | 24.667 - 458.250 | 59.400 - 3860.000 | 32.000 - 421.636 | 18.333 - 198.429 | 25.000 - 158.667 | 26.500 - 654.667 | 18.333 - 3860.000 |  |
| ALP variance |  |  |  |  |  |  |  | < 0.001 |
| N-Miss | 3 | 0 | 6 | 0 | 4 | 0 | 13 |  |
| Mean (SD) | 10.045 (11.127) | 123.398 (146.974) | 11.680 (13.816) | 18.905 (20.579) | 6.354 (8.249) | 43.104 (50.604) | 18.410 (38.850) |  |
| Range | 0.000 - 83.799 | 10.656 - 643.456 | 0.000 - 79.443 | 0.000 - 133.602 | 0.000 - 54.390 | 0.000 - 278.373 | 0.000 - 643.456 |  |
| Bilirubin (total) mean |  |  |  |  |  |  |  | < 0.001 |
| N-Miss | 4 | 0 | 6 | 0 | 4 | 0 | 14 |  |
| Mean (SD) | 10.957 (10.024) | 127.789 (168.264) | 11.467 (8.341) | 10.902 (8.278) | 9.310 (6.448) | 36.424 (58.221) | 17.179 (41.061) |  |
| Range | 3.000 - 103.667 | 4.300 - 554.714 | 3.000 - 57.000 | 3.000 - 73.375 | 3.000 - 54.250 | 3.333 - 385.000 | 3.000 - 554.714 |  |
| Bilirubin (total) variance |  |  |  |  |  |  |  | < 0.001 |
| N-Miss | 4 | 0 | 6 | 0 | 4 | 0 | 14 |  |
| Mean (SD) | 2.296 (2.796) | 39.440 (57.769) | 2.154 (2.642) | 3.097 (3.155) | 1.923 (4.235) | 9.477 (15.114) | 4.297 (13.044) |  |
| Range | 0.000 - 19.253 | 0.471 - 202.583 | 0.000 - 13.491 | 0.000 - 22.710 | 0.000 - 48.085 | 0.000 - 114.615 | 0.000 - 202.583 |  |
| CK mean |  |  |  |  |  |  |  | < 0.001 |
| Mean (SD) | 493.887 (809.503) | 4955.853 (15233.038) | 395.159 (1004.624) | 1080.601 (1914.624) | 861.806 (3355.812) | 1451.349 (6279.983) | 905.992 (3878.614) |  |
| Range | 12.000 - 5782.286 | 20.800 - 68697.167 | 7.000 - 6653.200 | 17.000 - 16578.500 | 21.500 - 37659.833 | 10.000 - 54524.250 | 7.000 - 68697.167 |  |
| CK variance |  |  |  |  |  |  |  | < 0.001 |
| Mean (SD) | 256.314 (547.400) | 3450.614 (9525.313) | 174.077 (545.634) | 588.717 (921.146) | 351.231 (1121.631) | 753.744 (2484.163) | 474.350 (2006.426) |  |
| Range | 0.000 - 4624.583 | 0.000 - 39751.232 | 0.000 - 3879.205 | 0.000 - 5205.988 | 0.000 - 9996.416 | 0.000 - 15029.164 | 0.000 - 39751.232 |  |
| CRP mean |  |  |  |  |  |  |  | < 0.001 |
| Mean (SD) | 88.817 (74.724) | 111.302 (90.996) | 165.666 (108.827) | 86.502 (72.389) | 46.776 (46.664) | 119.345 (92.250) | 94.453 (85.859) |  |
| Range | 0.300 - 429.667 | 8.450 - 444.600 | 0.800 - 438.250 | 1.450 - 360.500 | 0.300 - 250.250 | 5.640 - 402.444 | 0.300 - 444.600 |  |
| CRP variance |  |  |  |  |  |  |  | < 0.001 |
| Mean (SD) | 50.021 (39.543) | 47.154 (31.329) | 34.692 (29.915) | 53.144 (36.060) | 29.866 (27.499) | 49.480 (36.354) | 43.855 (35.769) |  |
| Range | 0.000 - 194.114 | 2.409 - 99.352 | 0.000 - 154.530 | 0.000 - 157.921 | 0.000 - 137.472 | 0.500 - 151.261 | 0.000 - 194.114 |  |
| Calcium mean |  |  |  |  |  |  |  | < 0.001 |
| N-Miss | 4 | 0 | 6 | 1 | 6 | 0 | 17 |  |
| Mean (SD) | 2.084 (0.154) | 2.006 (0.181) | 2.042 (0.181) | 1.999 (0.152) | 2.108 (0.118) | 1.955 (0.150) | 2.050 (0.159) |  |
| Range | 1.590 - 2.905 | 1.608 - 2.281 | 1.630 - 2.660 | 1.746 - 2.933 | 1.780 - 2.360 | 1.584 - 2.359 | 1.584 - 2.933 |  |
| Calcium variance |  |  |  |  |  |  |  | < 0.001 |
| N-Miss | 4 | 0 | 6 | 1 | 6 | 0 | 17 |  |
| Mean (SD) | 0.062 (0.053) | 0.128 (0.066) | 0.061 (0.054) | 0.096 (0.072) | 0.040 (0.041) | 0.115 (0.066) | 0.072 (0.062) |  |
| Range | 0.000 - 0.479 | 0.045 - 0.325 | 0.000 - 0.325 | 0.000 - 0.431 | 0.000 - 0.158 | 0.000 - 0.333 | 0.000 - 0.479 |  |
| Chloride mean |  |  |  |  |  |  |  | < 0.001 |
| N-Miss | 1 | 0 | 0 | 0 | 0 | 0 | 1 |  |
| Mean (SD) | 103.698 (4.292) | 104.302 (6.367) | 100.613 (7.989) | 105.151 (4.306) | 104.422 (3.460) | 104.883 (6.713) | 103.839 (5.397) |  |
| Range | 94.000 - 119.000 | 94.000 - 119.938 | 72.000 - 117.000 | 89.615 - 125.400 | 92.500 - 111.800 | 78.000 - 122.750 | 72.000 - 125.400 |  |
| Chloride variance |  |  |  |  |  |  |  | < 0.001 |
| N-Miss | 1 | 0 | 0 | 0 | 0 | 0 | 1 |  |
| Mean (SD) | 2.398 (1.424) | 3.372 (2.062) | 2.292 (2.371) | 2.400 (1.267) | 2.003 (1.386) | 3.585 (1.995) | 2.471 (1.718) |  |
| Range | 0.000 - 9.022 | 0.000 - 7.859 | 0.000 - 12.021 | 0.000 - 5.971 | 0.000 - 6.765 | 0.000 - 11.481 | 0.000 - 12.021 |  |
| Protein (total) mean |  |  |  |  |  |  |  | < 0.001 |
| N-Miss | 5 | 0 | 6 | 1 | 6 | 0 | 18 |  |
| Mean (SD) | 56.921 (8.001) | 47.785 (8.052) | 54.554 (7.761) | 49.833 (8.011) | 58.548 (7.369) | 47.794 (8.920) | 54.274 (8.899) |  |
| Range | 34.500 - 80.000 | 34.286 - 62.429 | 33.500 - 70.000 | 30.500 - 70.667 | 36.000 - 79.500 | 28.444 - 70.000 | 28.444 - 80.000 |  |
| Protein (total) variance |  |  |  |  |  |  |  | < 0.001 |
| N-Miss | 5 | 0 | 6 | 1 | 6 | 0 | 18 |  |
| Mean (SD) | 2.930 (2.571) | 4.341 (2.748) | 1.975 (1.668) | 5.258 (3.933) | 2.050 (2.267) | 4.782 (4.696) | 3.298 (3.297) |  |
| Range | 0.000 - 14.720 | 0.943 - 10.130 | 0.000 - 7.318 | 0.000 - 19.816 | 0.000 - 13.304 | 0.000 - 24.366 | 0.000 - 24.366 |  |
| Fibrinogen mean |  |  |  |  |  |  |  | < 0.001 |
| N-Miss | 37 | 0 | 14 | 6 | 18 | 4 | 79 |  |
| Mean (SD) | 3.510 (1.551) | 3.467 (2.508) | 5.585 (1.996) | 2.853 (1.373) | 2.911 (1.052) | 3.455 (1.999) | 3.533 (1.816) |  |
| Range | 0.450 - 9.000 | 0.680 - 9.967 | 1.700 - 9.000 | 0.711 - 8.667 | 1.100 - 7.300 | 0.911 - 8.967 | 0.450 - 9.967 |  |
| Fibrinogen variance |  |  |  |  |  |  |  | < 0.001 |
| N-Miss | 37 | 0 | 14 | 6 | 18 | 4 | 79 |  |
| Mean (SD) | 0.323 (0.569) | 0.604 (0.757) | 0.173 (0.383) | 0.557 (0.585) | 0.262 (0.490) | 0.523 (0.493) | 0.368 (0.547) |  |
| Range | 0.000 - 3.333 | 0.000 - 2.422 | 0.000 - 2.610 | 0.000 - 2.416 | 0.000 - 3.300 | 0.000 - 2.687 | 0.000 - 3.333 |  |
| Phosphate mean |  |  |  |  |  |  |  | < 0.001 |
| N-Miss | 1 | 0 | 0 | 0 | 0 | 0 | 1 |  |
| Mean (SD) | 1.013 (0.253) | 1.646 (0.641) | 1.171 (0.440) | 1.077 (0.332) | 0.972 (0.209) | 1.274 (0.395) | 1.088 (0.353) |  |
| Range | 0.470 - 2.366 | 0.895 - 3.447 | 0.497 - 2.790 | 0.305 - 2.318 | 0.550 - 1.600 | 0.686 - 2.558 | 0.305 - 3.447 |  |
| Phosphate variance |  |  |  |  |  |  |  | < 0.001 |
| N-Miss | 1 | 0 | 0 | 0 | 0 | 0 | 1 |  |
| Mean (SD) | 0.215 (0.130) | 0.431 (0.284) | 0.183 (0.152) | 0.287 (0.185) | 0.180 (0.114) | 0.313 (0.189) | 0.233 (0.165) |  |
| Range | 0.000 - 0.823 | 0.050 - 1.319 | 0.000 - 0.755 | 0.000 - 0.912 | 0.000 - 0.585 | 0.000 - 1.065 | 0.000 - 1.319 |  |
| Gamma-GT mean |  |  |  |  |  |  |  | < 0.001 |
| N-Miss | 3 | 0 | 6 | 0 | 4 | 0 | 13 |  |
| Mean (SD) | 70.342 (78.460) | 382.789 (422.695) | 122.072 (150.461) | 71.008 (74.896) | 57.985 (90.700) | 160.570 (169.406) | 94.985 (140.567) |  |
| Range | 7.000 - 769.333 | 32.000 - 1608.800 | 6.333 - 1074.429 | 8.333 - 473.000 | 7.000 - 931.667 | 10.500 - 876.333 | 6.333 - 1608.800 |  |
| Gamma-GT variance |  |  |  |  |  |  |  | < 0.001 |
| N-Miss | 3 | 0 | 6 | 0 | 4 | 0 | 13 |  |
| Mean (SD) | 14.223 (22.790) | 137.549 (166.468) | 12.937 (17.704) | 23.131 (31.990) | 9.494 (17.722) | 51.721 (55.293) | 22.815 (46.280) |  |
| Range | 0.000 - 160.329 | 3.487 - 571.626 | 0.000 - 110.820 | 0.000 - 184.403 | 0.000 - 124.000 | 0.000 - 261.937 | 0.000 - 571.626 |  |
| Haemoglobin mean |  |  |  |  |  |  |  | < 0.001 |
| Mean (SD) | 7.251 (1.241) | 5.722 (1.088) | 5.970 (0.943) | 6.607 (1.112) | 7.295 (1.178) | 5.727 (1.165) | 6.736 (1.309) |  |
| Range | 4.600 - 10.425 | 4.473 - 8.175 | 4.325 - 8.100 | 4.740 - 9.600 | 4.545 - 10.325 | 4.067 - 10.850 | 4.067 - 10.850 |  |
| Haemoglobin variance |  |  |  |  |  |  |  | < 0.001 |
| Mean (SD) | 0.603 (0.290) | 0.655 (0.223) | 0.362 (0.233) | 0.988 (0.435) | 0.529 (0.305) | 0.709 (0.430) | 0.635 (0.383) |  |
| Range | 0.050 - 1.949 | 0.249 - 1.291 | 0.000 - 1.160 | 0.050 - 2.060 | 0.000 - 1.806 | 0.112 - 2.173 | 0.000 - 2.173 |  |
| Haematocrit mean |  |  |  |  |  |  |  | < 0.001 |
| Mean (SD) | 0.359 (0.059) | 0.280 (0.054) | 0.295 (0.046) | 0.325 (0.055) | 0.356 (0.055) | 0.282 (0.053) | 0.332 (0.063) |  |
| Range | 0.220 - 0.505 | 0.210 - 0.407 | 0.214 - 0.400 | 0.222 - 0.482 | 0.202 - 0.515 | 0.197 - 0.500 | 0.197 - 0.515 |  |
| Haematocrit variance |  |  |  |  |  |  |  | < 0.001 |
| Mean (SD) | 0.029 (0.014) | 0.033 (0.012) | 0.017 (0.012) | 0.048 (0.021) | 0.025 (0.015) | 0.034 (0.022) | 0.030 (0.019) |  |
| Range | 0.000 - 0.097 | 0.015 - 0.064 | 0.000 - 0.054 | 0.005 - 0.106 | 0.000 - 0.083 | 0.005 - 0.111 | 0.000 - 0.111 |  |
| Potassium mean |  |  |  |  |  |  |  | < 0.001 |
| N-Miss | 50 | 3 | 30 | 24 | 25 | 26 | 158 |  |
| Mean (SD) | 4.288 (0.625) | 4.338 (0.831) | 4.250 (0.942) | 4.251 (0.666) | 3.804 (0.549) | 4.661 (1.564) | 4.207 (0.861) |  |
| Range | 2.800 - 7.900 | 2.700 - 6.400 | 2.300 - 8.100 | 2.600 - 6.300 | 2.400 - 6.700 | 2.800 - 13.000 | 2.300 - 13.000 |  |
| Potassium variance |  |  |  |  |  |  |  | < 0.001 |
| N-Miss | 50 | 3 | 30 | 24 | 25 | 26 | 158 |  |
| Mean (SD) | 0.059 (0.150) | 0.092 (0.189) | 0.099 (0.239) | 0.072 (0.211) | 0.025 (0.099) | 0.161 (0.345) | 0.071 (0.201) |  |
| Range | 0.000 - 0.950 | 0.000 - 0.793 | 0.000 - 1.268 | 0.000 - 1.556 | 0.000 - 0.750 | 0.000 - 1.850 | 0.000 - 1.850 |  |
| Creatinine mean |  |  |  |  |  |  |  | < 0.001 |
| Mean (SD) | 94.100 (62.697) | 231.330 (241.175) | 153.020 (153.451) | 109.347 (74.739) | 69.752 (24.262) | 143.875 (94.185) | 109.734 (97.539) |  |
| Range | 30.000 - 640.000 | 73.100 - 1146.750 | 27.000 - 859.250 | 5.500 - 591.250 | 30.167 - 202.250 | 7.750 - 559.857 | 5.500 - 1146.750 |  |
| Creatinine variance |  |  |  |  |  |  |  | < 0.001 |
| Mean (SD) | 12.651 (12.909) | 95.393 (177.529) | 20.121 (25.245) | 21.190 (22.552) | 7.518 (6.100) | 37.355 (43.231) | 19.438 (39.442) |  |
| Range | 0.500 - 93.060 | 12.004 - 863.565 | 0.000 - 131.395 | 0.500 - 116.355 | 0.000 - 40.938 | 1.700 - 322.472 | 0.000 - 863.565 |  |
| LDH mean |  |  |  |  |  |  |  | < 0.001 |
| Mean (SD) | 326.881 (226.527) | 1646.375 (1819.400) | 285.887 (136.136) | 452.285 (343.832) | 265.542 (137.160) | 728.939 (744.738) | 416.123 (513.871) |  |
| Range | 105.333 - 1473.333 | 181.000 - 6250.200 | 84.000 - 958.500 | 117.500 - 2595.125 | 114.250 - 1084.500 | 137.750 - 3483.333 | 84.000 - 6250.200 |  |
| LDH variance |  |  |  |  |  |  |  | < 0.001 |
| Mean (SD) | 80.323 (142.580) | 1016.060 (1486.380) | 41.558 (57.436) | 151.924 (236.243) | 43.497 (51.166) | 411.431 (705.468) | 146.330 (414.752) |  |
| Range | 0.000 - 1465.884 | 17.395 - 5767.509 | 0.000 - 444.575 | 1.886 - 1561.869 | 0.000 - 315.205 | 0.000 - 4121.881 | 0.000 - 5767.509 |  |
| Leukocytes mean |  |  |  |  |  |  |  | < 0.001 |
| Mean (SD) | 13.576 (4.908) | 27.168 (36.715) | 13.159 (6.424) | 14.491 (7.936) | 12.346 (4.062) | 15.359 (9.344) | 14.009 (8.969) |  |
| Range | 1.800 - 31.840 | 1.500 - 170.000 | 0.000 - 34.600 | 2.367 - 88.517 | 4.200 - 31.500 | 0.000 - 59.825 | 0.000 - 170.000 |  |
| Leukocytes variance |  |  |  |  |  |  |  | < 0.001 |
| Mean (SD) | 2.822 (1.947) | 9.886 (17.889) | 2.590 (1.890) | 3.712 (2.284) | 2.424 (1.491) | 4.671 (3.707) | 3.284 (3.885) |  |
| Range | 0.150 - 13.715 | 0.469 - 78.885 | 0.000 - 9.633 | 0.200 - 12.302 | 0.000 - 7.564 | 0.000 - 17.990 | 0.000 - 78.885 |  |
| Magnesium mean |  |  |  |  |  |  |  | < 0.001 |
| Mean (SD) | 0.816 (0.115) | 0.914 (0.101) | 0.825 (0.144) | 0.933 (0.224) | 0.804 (0.103) | 0.892 (0.218) | 0.847 (0.163) |  |
| Range | 0.500 - 1.187 | 0.765 - 1.120 | 0.510 - 1.335 | 0.520 - 2.115 | 0.557 - 1.209 | 0.608 - 1.950 | 0.500 - 2.115 |  |
| Magnesium variance |  |  |  |  |  |  |  | < 0.001 |
| Mean (SD) | 0.103 (0.069) | 0.105 (0.053) | 0.078 (0.071) | 0.174 (0.112) | 0.066 (0.055) | 0.130 (0.096) | 0.107 (0.087) |  |
| Range | 0.000 - 0.445 | 0.025 - 0.213 | 0.000 - 0.329 | 0.000 - 0.586 | 0.000 - 0.273 | 0.000 - 0.454 | 0.000 - 0.586 |  |
| Sodium mean |  |  |  |  |  |  |  | < 0.001 |
| Mean (SD) | 139.478 (3.434) | 140.256 (4.314) | 136.586 (7.497) | 139.508 (3.069) | 140.199 (2.974) | 139.839 (6.399) | 139.299 (4.681) |  |
| Range | 130.250 - 153.000 | 132.333 - 151.412 | 110.250 - 152.500 | 129.000 - 148.000 | 128.000 - 148.667 | 112.600 - 155.375 | 110.250 - 155.375 |  |
| Sodium variance |  |  |  |  |  |  |  | < 0.001 |
| Mean (SD) | 2.122 (1.281) | 3.280 (1.586) | 2.258 (1.921) | 2.401 (1.405) | 2.000 (1.262) | 3.072 (1.789) | 2.313 (1.520) |  |
| Range | 0.000 - 10.852 | 1.050 - 6.387 | 0.000 - 10.761 | 0.000 - 7.856 | 0.000 - 7.176 | 0.000 - 10.055 | 0.000 - 10.852 |  |
| Thrombocytes mean |  |  |  |  |  |  |  | < 0.001 |
| Mean (SD) | 231.064 (80.096) | 173.920 (152.598) | 296.100 (160.113) | 202.404 (81.108) | 243.619 (90.229) | 157.420 (109.179) | 227.281 (110.063) |  |
| Range | 33.500 - 596.250 | 18.500 - 496.000 | 18.250 - 868.812 | 60.529 - 469.833 | 82.500 - 650.750 | 16.900 - 666.294 | 16.900 - 868.812 |  |
| Thrombocytes variance |  |  |  |  |  |  |  | 0.003 |
| Mean (SD) | 38.482 (32.374) | 70.104 (105.683) | 37.492 (36.606) | 50.294 (44.555) | 41.360 (47.745) | 44.093 (34.448) | 42.618 (42.919) |  |
| Range | 1.700 - 199.616 | 7.499 - 507.755 | 0.000 - 209.163 | 5.831 - 291.505 | 0.000 - 305.059 | 4.546 - 214.063 | 0.000 - 507.755 |  |
| Urea mean |  |  |  |  |  |  |  | < 0.001 |
| Mean (SD) | 8.410 (3.997) | 16.380 (8.164) | 13.026 (9.796) | 9.441 (4.639) | 5.925 (2.413) | 13.588 (9.103) | 9.560 (6.607) |  |
| Range | 2.620 - 28.783 | 7.050 - 34.025 | 1.967 - 47.650 | 2.733 - 26.900 | 1.600 - 18.560 | 0.867 - 41.167 | 0.867 - 47.650 |  |
| Urea variance |  |  |  |  |  |  |  | < 0.001 |
| Mean (SD) | 1.426 (1.232) | 4.733 (3.350) | 2.242 (3.173) | 2.160 (2.103) | 1.062 (1.015) | 3.184 (2.898) | 1.899 (2.210) |  |
| Range | 0.000 - 8.905 | 1.066 - 13.742 | 0.000 - 18.464 | 0.050 - 11.198 | 0.000 - 8.742 | 0.000 - 15.387 | 0.000 - 18.464 |  |
| BMI |  |  |  |  |  |  |  | 0.002 |
| Mean (SD) | 27.656 (5.055) | 28.907 (6.218) | 26.478 (6.462) | 26.725 (4.866) | 25.808 (4.030) | 26.119 (5.935) | 26.768 (5.242) |  |
| Range | 15.960 - 50.810 | 19.030 - 38.870 | 14.460 - 50.190 | 16.650 - 45.910 | 16.330 - 43.670 | 13.870 - 57.370 | 13.870 - 57.370 |  |
| Previous ICU admission |  |  |  |  |  |  |  | < 0.001 |
| Mean (SD) | 193 (78.1%) | 21 (95.5%) | 105 (94.6%) | 127 (92.0%) | 164 (95.3%) | 89 (91.8%) | 699 (88.8%) |  |
| Range | 54 (21.9%) | 1 (4.5%) | 6 (5.4%) | 11 (8.0%) | 8 (4.7%) | 8 (8.2%) | 88 (11.2%) |  |
| Systolic blood pressure |  |  |  |  |  |  |  | < 0.001 |
| N-Miss | 2 | 0 | 0 | 0 | 0 | 0 | 2 |  |
| Mean (SD) | 127.041 (25.088) | 106.591 (22.800) | 120.964 (22.480) | 105.149 (17.570) | 128.061 (25.390) | 104.067 (18.422) | 119.145 (24.925) |  |
| Range | 80.000 - 199.000 | 68.000 - 165.000 | 65.500 - 212.000 | 66.000 - 170.000 | 76.000 - 211.000 | 64.000 - 175.000 | 64.000 - 212.000 |  |
| Diastolic blood pressure |  |  |  |  |  |  |  | < 0.001 |
| N-Miss | 2 | 0 | 0 | 0 | 0 | 0 | 2 |  |
| Mean (SD) | 64.549 (12.676) | 55.500 (10.069) | 59.176 (10.628) | 56.812 (8.874) | 64.945 (14.025) | 56.521 (8.877) | 61.270 (12.209) |  |
| Range | 35.500 - 103.000 | 37.000 - 81.000 | 35.500 - 87.000 | 26.000 - 82.000 | 36.000 - 147.000 | 40.500 - 82.000 | 26.000 - 147.000 |  |
| Mean arterial pressure |  |  |  |  |  |  |  | < 0.001 |
| N-Miss | 3 | 0 | 0 | 0 | 0 | 0 | 3 |  |
| Mean (SD) | 84.586 (15.287) | 71.455 (11.722) | 77.752 (11.918) | 71.790 (9.871) | 85.334 (16.489) | 70.577 (11.399) | 79.429 (15.055) |  |
| Range | 48.500 - 130.000 | 53.000 - 96.000 | 44.500 - 106.500 | 44.000 - 106.000 | 55.000 - 168.000 | 32.500 - 104.000 | 32.500 - 168.000 |  |
| Atrial fibrillation |  |  |  |  |  |  |  | < 0.001 |
| Mean (SD) | 209 (84.6%) | 21 (95.5%) | 104 (93.7%) | 135 (97.8%) | 171 (99.4%) | 93 (95.9%) | 733 (93.1%) |  |
| Range | 38 (15.4%) | 1 (4.5%) | 7 (6.3%) | 3 (2.2%) | 1 (0.6%) | 4 (4.1%) | 54 (6.9%) |  |
| Heart rate at admission |  |  |  |  |  |  |  | < 0.001 |
| N-Miss | 3 | 0 | 0 | 2 | 1 | 0 | 6 |  |
| Mean (SD) | 96.885 (23.109) | 104.273 (29.498) | 102.396 (27.286) | 92.456 (21.309) | 80.298 (17.709) | 109.227 (22.920) | 95.006 (24.319) |  |
| Range | 53.000 - 167.000 | 56.000 - 161.000 | 45.000 - 207.000 | 53.000 - 173.000 | 40.000 - 136.000 | 60.000 - 178.000 | 40.000 - 207.000 |  |
| Urine output in previous 6 hours |  |  |  |  |  |  |  | < 0.001 |
| N-Miss | 4 | 1 | 5 | 1 | 2 | 2 | 15 |  |
| Mean (SD) | 0.910 (0.750) | 0.334 (0.487) | 1.069 (0.885) | 0.852 (0.774) | 1.042 (0.892) | 0.678 (0.579) | 0.906 (0.796) |  |
| Range | 0.000 - 4.841 | 0.000 - 1.839 | 0.010 - 4.821 | 0.000 - 4.357 | 0.116 - 5.689 | 0.000 - 2.632 | 0.000 - 5.689 |  |
| Central venous pressure |  |  |  |  |  |  |  | < 0.001 |
| Not measured | 216 (87.4%) | 16 (72.7%) | 94 (84.7%) | 70 (50.7%) | 156 (90.7%) | 69 (71.1%) | 621 (78.9%) |  |
| Measured | 31 (12.6%) | 6 (27.3%) | 17 (15.3%) | 68 (49.3%) | 16 (9.3%) | 28 (28.9%) | 166 (21.1%) |  |
| Worsened respiratory condition |  |  |  |  |  |  |  | < 0.001 |
| No | 192 (77.7%) | 19 (86.4%) | 100 (90.1%) | 132 (95.7%) | 171 (99.4%) | 76 (78.4%) | 690 (87.7%) |  |
| Yes | 55 (22.3%) | 3 (13.6%) | 11 (9.9%) | 6 (4.3%) | 1 (0.6%) | 21 (21.6%) | 97 (12.3%) |  |
| Tidal volume |  |  |  |  |  |  |  | 0.033 |
| N-Miss | 131 | 8 | 72 | 28 | 56 | 35 | 330 |  |
| Mean (SD) | 540.578 (131.912) | 463.571 (189.030) | 491.103 (102.364) | 512.423 (117.983) | 502.000 (85.078) | 495.516 (137.781) | 511.314 (119.798) |  |
| Range | 350.000 - 1249.000 | 10.000 - 650.000 | 263.000 - 750.000 | 0.500 - 987.000 | 50.000 - 740.000 | 50.000 - 1130.000 | 0.500 - 1249.000 |  |
| Respiratory rate |  |  |  |  |  |  |  | < 0.001 |
| N-Miss | 133 | 7 | 73 | 27 | 56 | 34 | 330 |  |
| Mean (SD) | 14.294 (3.934) | 16.200 (5.480) | 15.079 (4.950) | 17.000 (3.830) | 14.583 (3.687) | 18.952 (6.049) | 15.795 (4.627) |  |
| Range | 5.000 - 27.000 | 10.000 - 26.000 | 6.000 - 27.000 | 8.000 - 26.000 | 2.600 - 27.000 | 8.000 - 36.000 | 2.600 - 36.000 |  |
| Positive end-expiratory pressure |  |  |  |  |  |  |  | < 0.001 |
| N-Miss | 129 | 7 | 71 | 27 | 56 | 34 | 324 |  |
| Mean (SD) | 7.064 (1.958) | 8.867 (2.200) | 7.975 (2.154) | 7.572 (2.578) | 6.103 (2.002) | 8.381 (2.282) | 7.261 (2.336) |  |
| Range | 2.000 - 12.000 | 5.000 - 12.000 | 5.000 - 14.000 | 4.000 - 15.000 | 5.000 - 14.000 | 5.000 - 14.000 | 2.000 - 15.000 |  |
| Mechanical ventilation after 24h |  |  |  |  |  |  |  | < 0.001 |
| No | 89 (36.0%) | 5 (22.7%) | 58 (52.3%) | 7 (5.1%) | 31 (18.0%) | 15 (15.5%) | 205 (26.0%) |  |
| Yes | 158 (64.0%) | 17 (77.3%) | 53 (47.7%) | 131 (94.9%) | 141 (82.0%) | 82 (84.5%) | 582 (74.0%) |  |
| Mechanical ventilation at admission |  |  |  |  |  |  |  | < 0.001 |
| No | 144 (58.3%) | 8 (36.4%) | 69 (62.2%) | 13 (9.4%) | 32 (18.6%) | 36 (37.1%) | 302 (38.4%) |  |
| Yes | 103 (41.7%) | 14 (63.6%) | 42 (37.8%) | 125 (90.6%) | 140 (81.4%) | 61 (62.9%) | 485 (61.6%) |  |
| Respiratory rate |  |  |  |  |  |  |  | < 0.001 |
| N-Miss | 1 | 0 | 0 | 2 | 0 | 0 | 3 |  |
| Mean (SD) | 17.565 (4.966) | 20.227 (8.199) | 20.405 (5.956) | 17.750 (5.094) | 15.895 (3.898) | 20.649 (7.601) | 18.089 (5.678) |  |
| Range | 5.000 - 33.000 | 10.000 - 40.000 | 10.000 - 45.000 | 7.000 - 35.000 | 7.000 - 27.000 | 7.000 - 58.000 | 5.000 - 58.000 |  |
| FiO2 low |  |  |  |  |  |  |  | < 0.001 |
| N-Miss | 4 | 0 | 2 | 0 | 0 | 0 | 6 |  |
| Mean (SD) | 47.416 (20.863) | 56.409 (22.240) | 48.220 (24.573) | 53.290 (25.972) | 35.140 (13.813) | 54.660 (25.119) | 47.015 (22.760) |  |
| Range | 21.000 - 100.000 | 21.000 - 100.000 | 21.000 - 100.000 | 21.000 - 100.000 | 21.000 - 100.000 | 21.000 - 100.000 | 21.000 - 100.000 |  |
| Myocardial infarction (history) |  |  |  |  |  |  |  | < 0.001 |
| No | 239 (96.8%) | 21 (95.5%) | 108 (97.3%) | 109 (79.0%) | 155 (90.1%) | 92 (94.8%) | 724 (92.0%) |  |
| Yes | 8 (3.2%) | 1 (4.5%) | 3 (2.7%) | 29 (21.0%) | 17 (9.9%) | 5 (5.2%) | 63 (8.0%) |  |
| Diabetes (history) |  |  |  |  |  |  |  | < 0.001 |
| No | 239 (96.8%) | 21 (95.5%) | 108 (97.3%) | 109 (79.0%) | 155 (90.1%) | 92 (94.8%) | 724 (92.0%) |  |
| Yes | 8 (3.2%) | 1 (4.5%) | 3 (2.7%) | 29 (21.0%) | 17 (9.9%) | 5 (5.2%) | 63 (8.0%) |  |
| Cardiovascular disease (history) |  |  |  |  |  |  |  | < 0.001 |
| No | 174 (70.4%) | 19 (86.4%) | 78 (70.3%) | 112 (81.2%) | 163 (94.8%) | 80 (82.5%) | 626 (79.5%) |  |
| Yes | 73 (29.6%) | 3 (13.6%) | 33 (29.7%) | 26 (18.8%) | 9 (5.2%) | 17 (17.5%) | 161 (20.5%) |  |
| Chronic Obstructive pulmonary disease (history) |  |  |  |  |  |  |  | < 0.001 |
| No | 235 (95.1%) | 22 (100.0%) | 107 (96.4%) | 120 (87.0%) | 170 (98.8%) | 94 (96.9%) | 748 (95.0%) |  |
| Yes | 12 (4.9%) | 0 (0.0%) | 4 (3.6%) | 18 (13.0%) | 2 (1.2%) | 3 (3.1%) | 39 (5.0%) |  |
| Respiratory insufficiency (history) |  |  |  |  |  |  |  | 0.006 |
| No | 204 (82.6%) | 22 (100.0%) | 92 (82.9%) | 127 (92.0%) | 156 (90.7%) | 88 (90.7%) | 689 (87.5%) |  |
| Yes | 43 (17.4%) | 0 (0.0%) | 19 (17.1%) | 11 (8.0%) | 16 (9.3%) | 9 (9.3%) | 98 (12.5%) |  |
| Chronic kidney disease (history) |  |  |  |  |  |  |  | 0.383 |
| No | 234 (94.7%) | 21 (95.5%) | 107 (96.4%) | 126 (91.3%) | 166 (96.5%) | 93 (95.9%) | 747 (94.9%) |  |
| Yes | 13 (5.3%) | 1 (4.5%) | 4 (3.6%) | 12 (8.7%) | 6 (3.5%) | 4 (4.1%) | 40 (5.1%) |  |
| Dialysis (history) |  |  |  |  |  |  |  | < 0.001 |
| No | 237 (96.0%) | 19 (86.4%) | 91 (82.0%) | 131 (94.9%) | 171 (99.4%) | 84 (86.6%) | 733 (93.1%) |  |
| Yes | 10 (4.0%) | 3 (13.6%) | 20 (18.0%) | 7 (5.1%) | 1 (0.6%) | 13 (13.4%) | 54 (6.9%) |  |
| Cirrhosis (history) |  |  |  |  |  |  |  | 0.106 |
| No | 246 (99.6%) | 22 (100.0%) | 108 (97.3%) | 135 (97.8%) | 172 (100.0%) | 94 (96.9%) | 777 (98.7%) |  |
| Yes | 1 (0.4%) | 0 (0.0%) | 3 (2.7%) | 3 (2.2%) | 0 (0.0%) | 3 (3.1%) | 10 (1.3%) |  |
| Metastatic disease (history) |  |  |  |  |  |  |  | < 0.001 |
| No | 244 (98.8%) | 19 (86.4%) | 108 (97.3%) | 133 (96.4%) | 171 (99.4%) | 87 (89.7%) | 762 (96.8%) |  |
| Yes | 3 (1.2%) | 3 (13.6%) | 3 (2.7%) | 5 (3.6%) | 1 (0.6%) | 10 (10.3%) | 25 (3.2%) |  |
| Haematological malignancy (history) |  |  |  |  |  |  |  | 0.019 |
| No | 240 (97.2%) | 19 (86.4%) | 106 (95.5%) | 131 (94.9%) | 171 (99.4%) | 93 (95.9%) | 760 (96.6%) |  |
| Yes | 7 (2.8%) | 3 (13.6%) | 5 (4.5%) | 7 (5.1%) | 1 (0.6%) | 4 (4.1%) | 27 (3.4%) |  |
| Immune insufficiency (history) |  |  |  |  |  |  |  | < 0.001 |
| No | 243 (98.4%) | 15 (68.2%) | 103 (92.8%) | 136 (98.6%) | 172 (100.0%) | 87 (89.7%) | 756 (96.1%) |  |
| Yes | 4 (1.6%) | 7 (31.8%) | 8 (7.2%) | 2 (1.4%) | 0 (0.0%) | 10 (10.3%) | 31 (3.9%) |  |
