# Supplementary material for: Deep embedded clustering generalisability and adaptation for integrating mixed datatypes: two critical care cohorts
Source: Sci Rep. 2024 Jan 10;14:1045. doi: 10.1038/s41598-024-51699-z (PMC10781731; doi:10.1038/s41598-024-51699-z)
Supplement: Supplementary file 4 — Supplementary Table S5. [file 41598_2024_51699_MOESM4_ESM.docx]

**Table S5. Descriptive statistics of the input and outcome variables for the recreated DEC clusters on the MUMC+ dataset.** The first column specifies the variables (in bold), and whether the variables is described by its mean with standard deviation (SD) and the range of values, or if it is a category, the number of patients in each level, and how much percentage of the patients fall in that level. If the variable is missing for some samples, this is indicated by ‘N-miss’, which specifies the number of samples for which this variable was missing. The other columns specify the different clusters, and complete dataset. The last column specifies the p-value. The Chi-square test was used for categorical variables, and the Kruskal-Wallis Rank Test for numeric variables.

|  | Cluster 1 (N=801) | Cluster 2 (N=219) | Cluster 3 (N=916) | Cluster 4 (N=241) | Cluster 5 (N=888) | Cluster 6 (N=829) | Total (N=3894) | p value |
| --- | --- | --- | --- | --- | --- | --- | --- | --- |
| In-ICU mortality |  |  |  |  |  |  |  | < 0.001 |
| Survivor | 628 (78.4%) | 117 (53.4%) | 804 (87.8%) | 180 (74.7%) | 750 (84.5%) | 538 (64.9%) | 3017 (77.5%) |  |
| Non-survivor | 173 (21.6%) | 102 (46.6%) | 112 (12.2%) | 61 (25.3%) | 138 (15.5%) | 291 (35.1%) | 877 (22.5%) |  |
| Length of stay |  |  |  |  |  |  |  | < 0.001 |
| Mean (SD) | 6.801 (10.778) | 13.337 (17.316) | 6.378 (11.146) | 6.747 (9.160) | 5.310 (6.752) | 11.603 (15.460) | 7.748 (11.968) |  |
| Range | 1.003 - 150.875 | 1.014 - 141.601 | 1.010 - 132.375 | 1.073 - 68.757 | 1.003 - 66.792 | 1.003 - 188.375 | 1.003 - 188.375 |  |
| Apache IV mortality |  |  |  |  |  |  |  | < 0.001 |
| N-Miss | 132 | 49 | 266 | 32 | 120 | 177 | 776 |  |
| Mean (SD) | 0.397 (0.280) | 0.534 (0.270) | 0.292 (0.226) | 0.416 (0.276) | 0.269 (0.243) | 0.479 (0.286) | 0.369 (0.276) |  |
| Range | 0.002 - 0.996 | 0.028 - 0.985 | 0.002 - 0.984 | 0.005 - 0.971 | 0.003 - 0.952 | 0.005 - 0.994 | 0.002 - 0.996 |  |
| Apache IV score |  |  |  |  |  |  |  | < 0.001 |
| N-Miss | 132 | 49 | 266 | 32 | 120 | 177 | 776 |  |
| Mean (SD) | 79.513 (27.992) | 102.059 (30.687) | 71.251 (24.403) | 90.211 (29.130) | 64.507 (27.143) | 96.914 (32.444) | 79.679 (31.131) |  |
| Range | 12.000 - 195.000 | 23.000 - 190.000 | 17.000 - 188.000 | 31.000 - 184.000 | 9.000 - 175.000 | 11.000 - 217.000 | 9.000 - 217.000 |  |
| SAPS II score |  |  |  |  |  |  |  | < 0.001 |
| Mean (SD) | 45.707 (14.701) | 54.840 (17.491) | 38.957 (13.642) | 51.033 (15.615) | 39.818 (15.084) | 52.251 (16.837) | 45.013 (16.315) |  |
| Range | 10.000 - 99.000 | 15.000 - 97.000 | 6.000 - 92.000 | 21.000 - 95.000 | 6.000 - 86.000 | 14.000 - 113.000 | 6.000 - 113.000 |  |
| Post-operative |  |  |  |  |  |  |  | < 0.001 |
| No | 720 (89.9%) | 171 (78.1%) | 720 (78.6%) | 148 (61.4%) | 604 (68.0%) | 572 (69.0%) | 2935 (75.4%) |  |
| Yes | 81 (10.1%) | 48 (21.9%) | 196 (21.4%) | 93 (38.6%) | 284 (32.0%) | 257 (31.0%) | 959 (24.6%) |  |
| Admission diagnosis (Based on APACHE IV) |  |  |  |  |  |  |  | < 0.001 |
|  | 12 (1.5%) | 0 (0.0%) | 14 (1.5%) | 5 (2.1%) | 16 (1.8%) | 13 (1.6%) | 60 (1.5%) |  |
| Cardiovascular | 282 (35.2%) | 45 (20.5%) | 248 (27.1%) | 144 (59.8%) | 257 (28.9%) | 291 (35.1%) | 1267 (32.5%) |  |
| Gastrointestinal | 49 (6.1%) | 62 (28.3%) | 167 (18.2%) | 22 (9.1%) | 45 (5.1%) | 157 (18.9%) | 502 (12.9%) |  |
| Genito-urinary | 5 (0.6%) | 3 (1.4%) | 26 (2.8%) | 1 (0.4%) | 3 (0.3%) | 9 (1.1%) | 47 (1.2%) |  |
| Haematological | 13 (1.6%) | 40 (18.3%) | 23 (2.5%) | 2 (0.8%) | 3 (0.3%) | 77 (9.3%) | 158 (4.1%) |  |
| Metabolic | 17 (2.1%) | 10 (4.6%) | 31 (3.4%) | 4 (1.7%) | 16 (1.8%) | 30 (3.6%) | 108 (2.8%) |  |
| Musculoskeletal/skin | 2 (0.2%) | 2 (0.9%) | 6 (0.7%) | 3 (1.2%) | 4 (0.5%) | 6 (0.7%) | 23 (0.6%) |  |
| Neurological | 179 (22.3%) | 8 (3.7%) | 46 (5.0%) | 16 (6.6%) | 284 (32.0%) | 49 (5.9%) | 582 (14.9%) |  |
| Respiratory | 193 (24.1%) | 48 (21.9%) | 335 (36.6%) | 30 (12.4%) | 153 (17.2%) | 151 (18.2%) | 910 (23.4%) |  |
| Transplant | 0 (0.0%) | 0 (0.0%) | 1 (0.1%) | 1 (0.4%) | 0 (0.0%) | 9 (1.1%) | 11 (0.3%) |  |
| Trauma | 49 (6.1%) | 1 (0.5%) | 19 (2.1%) | 13 (5.4%) | 107 (12.0%) | 37 (4.5%) | 226 (5.8%) |  |
| Age |  |  |  |  |  |  |  | < 0.001 |
| N-Miss | 0 | 0 | 1 | 0 | 0 | 0 | 1 |  |
| Mean (SD) | 66.983 (12.938) | 59.831 (14.002) | 63.089 (15.051) | 66.548 (12.946) | 59.742 (17.493) | 61.323 (14.777) | 62.781 (15.255) |  |
| Range | 19.000 - 100.000 | 19.000 - 99.000 | 18.000 - 92.000 | 22.000 - 100.000 | 18.000 - 100.000 | 18.000 - 102.000 | 18.000 - 102.000 |  |
| Gender |  |  |  |  |  |  |  | < 0.001 |
| Female | 285 (35.6%) | 59 (26.9%) | 372 (40.6%) | 72 (29.9%) | 367 (41.3%) | 262 (31.6%) | 1417 (36.4%) |  |
| Male | 516 (64.4%) | 160 (73.1%) | 544 (59.4%) | 169 (70.1%) | 521 (58.7%) | 567 (68.4%) | 2477 (63.6%) |  |
| vasoactive |  |  |  |  |  |  |  | < 0.001 |
| No | 225 (28.1%) | 23 (10.5%) | 329 (35.9%) | 6 (2.5%) | 276 (31.1%) | 60 (7.2%) | 919 (23.6%) |  |
| Yes | 576 (71.9%) | 196 (89.5%) | 587 (64.1%) | 235 (97.5%) | 612 (68.9%) | 769 (92.8%) | 2975 (76.4%) |  |
| Renal replacement therapy |  |  |  |  |  |  |  | < 0.001 |
| No | 765 (95.5%) | 163 (74.4%) | 821 (89.6%) | 223 (92.5%) | 874 (98.4%) | 664 (80.1%) | 3510 (90.1%) |  |
| Yes | 36 (4.5%) | 56 (25.6%) | 95 (10.4%) | 18 (7.5%) | 14 (1.6%) | 165 (19.9%) | 384 (9.9%) |  |
| NOR |  |  |  |  |  |  |  | < 0.001 |
| No | 628 (78.4%) | 117 (53.4%) | 804 (87.8%) | 180 (74.7%) | 750 (84.5%) | 538 (64.9%) | 3017 (77.5%) |  |
| Yes | 173 (21.6%) | 102 (46.6%) | 112 (12.2%) | 61 (25.3%) | 138 (15.5%) | 291 (35.1%) | 877 (22.5%) |  |
| ICU readmission |  |  |  |  |  |  |  | < 0.001 |
| 1st readmission | 83 (10.4%) | 18 (8.2%) | 124 (13.5%) | 11 (4.6%) | 38 (4.3%) | 61 (7.4%) | 335 (8.6%) |  |
| 2nd readmission | 13 (1.6%) | 4 (1.8%) | 22 (2.4%) | 0 (0.0%) | 5 (0.6%) | 11 (1.3%) | 55 (1.4%) |  |
| no readmission | 694 (86.6%) | 197 (90.0%) | 760 (83.0%) | 230 (95.4%) | 839 (94.5%) | 751 (90.6%) | 3471 (89.1%) |  |
| EMV score |  |  |  |  |  |  |  | < 0.001 |
| N-Miss | 147 | 49 | 240 | 48 | 133 | 224 | 841 |  |
| Mean (SD) | 8.826 (5.313) | 9.335 (5.657) | 11.496 (4.849) | 6.503 (5.095) | 6.657 (4.962) | 7.853 (5.432) | 8.569 (5.458) |  |
| Range | 3.000 - 15.000 | 3.000 - 15.000 | 3.000 - 15.000 | 3.000 - 15.000 | 1.000 - 15.000 | 3.000 - 15.000 | 1.000 - 15.000 |  |
| Temperature center |  |  |  |  |  |  |  | < 0.001 |
| N-Miss | 2 | 1 | 4 | 0 | 1 | 0 | 8 |  |
| Mean (SD) | 36.071 (1.784) | 36.665 (1.893) | 36.852 (1.270) | 35.393 (1.970) | 35.874 (1.452) | 36.298 (1.979) | 36.249 (1.723) |  |
| Range | 22.700 - 40.400 | 22.000 - 40.500 | 30.600 - 41.000 | 23.400 - 39.700 | 20.700 - 40.100 | 24.200 - 41.900 | 20.700 - 41.900 |  |
| Admission type |  |  |  |  |  |  |  | < 0.001 |
| Acute surgery | 79 (9.9%) | 36 (16.4%) | 145 (15.8%) | 100 (41.5%) | 281 (31.6%) | 212 (25.6%) | 853 (21.9%) |  |
| Medical | 722 (90.1%) | 183 (83.6%) | 771 (84.2%) | 141 (58.5%) | 607 (68.4%) | 617 (74.4%) | 3041 (78.1%) |  |
| ALAT mean |  |  |  |  |  |  |  | < 0.001 |
| N-Miss | 4 | 1 | 5 | 1 | 9 | 1 | 21 |  |
| Mean (SD) | 76.956 (129.600) | 531.808 (1185.605) | 51.892 (104.696) | 122.166 (312.243) | 58.867 (97.563) | 217.688 (454.560) | 125.446 (388.582) |  |
| Range | 7.000 - 1728.333 | 8.667 - 8185.400 | 5.000 - 1230.000 | 7.000 - 3873.273 | 6.500 - 1430.500 | 5.000 - 3500.500 | 5.000 - 8185.400 |  |
| ALAT variance |  |  |  |  |  |  |  | < 0.001 |
| N-Miss | 4 | 1 | 5 | 1 | 9 | 1 | 21 |  |
| Mean (SD) | 27.305 (67.910) | 336.617 (768.693) | 13.117 (41.040) | 58.987 (179.922) | 19.122 (58.858) | 126.311 (323.084) | 62.650 (256.702) |  |
| Range | 0.000 - 1096.500 | 0.000 - 4718.108 | 0.000 - 697.973 | 0.000 - 2155.673 | 0.000 - 969.500 | 0.000 - 4218.825 | 0.000 - 4718.108 |  |
| ASAT mean |  |  |  |  |  |  |  | < 0.001 |
| N-Miss | 4 | 2 | 3 | 1 | 9 | 2 | 21 |  |
| Mean (SD) | 100.644 (169.537) | 1037.586 (2223.369) | 64.589 (122.428) | 172.321 (321.394) | 78.419 (150.897) | 317.019 (622.632) | 190.240 (656.773) |  |
| Range | 9.833 - 1964.500 | 11.250 - 12116.750 | 6.667 - 1881.000 | 10.250 - 3378.091 | 7.500 - 2848.333 | 10.000 - 5140.250 | 6.667 - 12116.750 |  |
| ASAT variance |  |  |  |  |  |  |  | < 0.001 |
| N-Miss | 4 | 2 | 3 | 1 | 9 | 2 | 21 |  |
| Mean (SD) | 46.942 (131.001) | 827.911 (1799.383) | 20.272 (65.380) | 114.441 (374.911) | 29.858 (114.502) | 248.741 (593.923) | 127.807 (555.795) |  |
| Range | 0.000 - 1876.500 | 0.000 - 8784.455 | 0.000 - 848.093 | 0.000 - 4192.569 | 0.000 - 2868.779 | 0.000 - 5077.154 | 0.000 - 8784.455 |  |
| Albumin mean |  |  |  |  |  |  |  | < 0.001 |
| N-Miss | 132 | 12 | 154 | 33 | 202 | 74 | 607 |  |
| Mean (SD) | 27.641 (5.902) | 16.468 (5.669) | 21.130 (5.898) | 22.151 (5.156) | 28.058 (5.732) | 19.238 (5.780) | 23.238 (7.057) |  |
| Range | 12.820 - 48.300 | 7.150 - 41.633 | 7.557 - 39.500 | 11.100 - 37.700 | 11.900 - 44.500 | 4.200 - 40.900 | 4.200 - 48.300 |  |
| Albumin variance |  |  |  |  |  |  |  | < 0.001 |
| N-Miss | 132 | 12 | 154 | 33 | 202 | 74 | 607 |  |
| Mean (SD) | 1.909 (1.932) | 1.946 (1.723) | 0.994 (1.305) | 2.031 (2.098) | 1.474 (1.857) | 1.956 (1.851) | 1.627 (1.810) |  |
| Range | 0.000 - 9.976 | 0.000 - 7.900 | 0.000 - 7.797 | 0.000 - 10.150 | 0.000 - 9.300 | 0.000 - 14.114 | 0.000 - 14.114 |  |
| ALP mean |  |  |  |  |  |  |  | < 0.001 |
| N-Miss | 20 | 2 | 52 | 8 | 61 | 18 | 161 |  |
| Mean (SD) | 97.009 (45.376) | 362.717 (373.824) | 132.104 (79.456) | 89.605 (47.497) | 83.205 (35.443) | 156.693 (112.672) | 130.024 (131.422) |  |
| Range | 32.333 - 494.500 | 50.500 - 2799.667 | 21.000 - 630.500 | 30.750 - 503.750 | 16.500 - 303.000 | 29.000 - 979.500 | 16.500 - 2799.667 |  |
| ALP variance |  |  |  |  |  |  |  | < 0.001 |
| N-Miss | 20 | 2 | 52 | 8 | 61 | 18 | 161 |  |
| Mean (SD) | 13.485 (17.540) | 143.466 (163.824) | 15.632 (21.785) | 17.535 (22.045) | 11.069 (17.029) | 45.119 (46.501) | 28.128 (57.270) |  |
| Range | 0.000 - 181.015 | 0.000 - 909.332 | 0.000 - 162.398 | 0.000 - 188.668 | 0.000 - 131.216 | 0.000 - 242.989 | 0.000 - 909.332 |  |
| Bilirubin (total) mean |  |  |  |  |  |  |  | < 0.001 |
| N-Miss | 45 | 3 | 66 | 6 | 56 | 17 | 193 |  |
| Mean (SD) | 11.187 (9.166) | 75.098 (105.663) | 11.712 (12.550) | 13.051 (10.425) | 9.880 (6.866) | 23.519 (32.302) | 17.568 (34.351) |  |
| Range | 2.400 - 95.300 | 3.050 - 635.629 | 2.100 - 139.075 | 2.300 - 71.125 | 2.200 - 71.029 | 2.100 - 297.500 | 2.100 - 635.629 |  |
| Bilirubin (total) variance |  |  |  |  |  |  |  | < 0.001 |
| N-Miss | 45 | 3 | 66 | 6 | 56 | 17 | 193 |  |
| Mean (SD) | 2.289 (3.014) | 21.548 (28.940) | 1.809 (2.808) | 3.213 (3.554) | 1.819 (2.541) | 6.346 (8.793) | 4.146 (9.669) |  |
| Range | 0.000 - 31.150 | 0.000 - 163.082 | 0.000 - 22.637 | 0.000 - 23.962 | 0.000 - 26.699 | 0.000 - 79.920 | 0.000 - 163.082 |  |
| CK mean |  |  |  |  |  |  |  | < 0.001 |
| N-Miss | 54 | 13 | 109 | 4 | 95 | 38 | 313 |  |
| Mean (SD) | 651.351 (1344.444) | 1716.646 (7511.577) | 359.216 (1252.948) | 991.441 (1869.252) | 650.046 (1216.642) | 1072.784 (3115.061) | 762.107 (2603.716) |  |
| Range | 8.750 - 16156.500 | 10.250 - 85000.667 | 7.000 - 20818.000 | 16.333 - 15540.667 | 11.000 - 10415.571 | 9.200 - 45617.833 | 7.000 - 85000.667 |  |
| CK variance |  |  |  |  |  |  |  | < 0.001 |
| N-Miss | 54 | 13 | 109 | 4 | 95 | 38 | 313 |  |
| Mean (SD) | 327.857 (704.267) | 948.117 (3159.242) | 141.014 (602.225) | 572.230 (1262.364) | 283.804 (645.838) | 678.586 (1947.828) | 405.321 (1358.918) |  |
| Range | 0.000 - 5634.855 | 0.000 - 31656.416 | 0.000 - 10048.825 | 0.000 - 11958.044 | 0.000 - 6640.871 | 0.000 - 21530.112 | 0.000 - 31656.416 |  |
| CRP mean |  |  |  |  |  |  |  | < 0.001 |
| N-Miss | 0 | 1 | 0 | 2 | 11 | 3 | 17 |  |
| Mean (SD) | 91.585 (67.027) | 150.965 (100.621) | 144.787 (97.588) | 116.643 (68.502) | 71.995 (59.703) | 140.745 (82.762) | 115.081 (85.144) |  |
| Range | 1.000 - 478.333 | 2.000 - 598.333 | 1.333 - 541.667 | 1.000 - 431.667 | 1.000 - 338.200 | 2.333 - 586.250 | 1.000 - 598.333 |  |
| CRP variance |  |  |  |  |  |  |  | < 0.001 |
| N-Miss | 0 | 1 | 0 | 2 | 11 | 3 | 17 |  |
| Mean (SD) | 52.633 (37.385) | 60.454 (40.840) | 40.698 (34.179) | 60.443 (36.164) | 42.346 (37.416) | 61.802 (39.705) | 50.361 (38.307) |  |
| Range | 0.000 - 213.671 | 0.000 - 225.975 | 0.000 - 180.441 | 0.000 - 184.252 | 0.000 - 185.146 | 0.000 - 197.102 | 0.000 - 225.975 |  |
| Calcium mean |  |  |  |  |  |  |  | < 0.001 |
| N-Miss | 74 | 6 | 114 | 27 | 119 | 47 | 387 |  |
| Mean (SD) | 2.103 (0.135) | 1.879 (0.197) | 2.013 (0.175) | 1.958 (0.152) | 2.083 (0.153) | 1.935 (0.176) | 2.018 (0.179) |  |
| Range | 1.580 - 2.670 | 1.265 - 2.595 | 1.247 - 2.735 | 1.465 - 2.320 | 1.530 - 2.844 | 1.310 - 2.732 | 1.247 - 2.844 |  |
| Calcium variance |  |  |  |  |  |  |  | < 0.001 |
| N-Miss | 74 | 6 | 114 | 27 | 119 | 47 | 387 |  |
| Mean (SD) | 0.069 (0.057) | 0.114 (0.088) | 0.053 (0.053) | 0.084 (0.086) | 0.053 (0.054) | 0.100 (0.075) | 0.072 (0.068) |  |
| Range | 0.000 - 0.372 | 0.000 - 0.528 | 0.000 - 0.423 | 0.000 - 0.525 | 0.000 - 0.299 | 0.000 - 0.579 | 0.000 - 0.579 |  |
| Chloride mean |  |  |  |  |  |  |  | < 0.001 |
| N-Miss | 82 | 11 | 125 | 33 | 141 | 62 | 454 |  |
| Mean (SD) | 106.524 (5.137) | 109.533 (7.593) | 105.260 (6.500) | 110.058 (4.948) | 107.887 (5.350) | 110.089 (6.689) | 107.720 (6.310) |  |
| Range | 89.279 - 132.000 | 78.400 - 136.000 | 73.000 - 124.000 | 96.667 - 127.000 | 84.000 - 138.000 | 81.333 - 138.000 | 73.000 - 138.000 |  |
| Chloride variance |  |  |  |  |  |  |  | < 0.001 |
| N-Miss | 82 | 11 | 125 | 33 | 141 | 62 | 454 |  |
| Mean (SD) | 2.320 (1.862) | 3.938 (2.913) | 1.887 (2.084) | 1.837 (1.874) | 1.933 (2.055) | 3.554 (2.688) | 2.480 (2.352) |  |
| Range | 0.000 - 9.836 | 0.000 - 16.000 | 0.000 - 17.250 | 0.000 - 10.055 | 0.000 - 12.000 | 0.000 - 18.707 | 0.000 - 18.707 |  |
| Protein (total) mean |  |  |  |  |  |  |  | < 0.001 |
| N-Miss | 335 | 47 | 394 | 92 | 398 | 252 | 1518 |  |
| Mean (SD) | 57.098 (7.413) | 44.649 (11.103) | 52.176 (8.234) | 47.966 (8.574) | 55.083 (8.125) | 46.967 (8.869) | 51.667 (9.477) |  |
| Range | 34.000 - 89.600 | 18.300 - 100.912 | 25.400 - 83.200 | 21.100 - 77.500 | 33.500 - 88.400 | 12.500 - 85.300 | 12.500 - 100.912 |  |
| Protein (total) variance |  |  |  |  |  |  |  | < 0.001 |
| N-Miss | 335 | 47 | 394 | 92 | 398 | 252 | 1518 |  |
| Mean (SD) | 1.634 (2.336) | 2.617 (3.254) | 0.949 (1.630) | 2.276 (2.879) | 1.465 (2.568) | 2.476 (2.991) | 1.765 (2.611) |  |
| Range | 0.000 - 12.500 | 0.000 - 18.460 | 0.000 - 9.343 | 0.000 - 14.038 | 0.000 - 18.528 | 0.000 - 16.500 | 0.000 - 18.528 |  |
| Fibrinogen mean |  |  |  |  |  |  |  | < 0.001 |
| N-Miss | 296 | 39 | 339 | 45 | 308 | 167 | 1194 |  |
| Mean (SD) | 3.938 (1.408) | 4.207 (1.996) | 5.324 (1.846) | 2.841 (1.382) | 3.284 (1.428) | 4.059 (1.973) | 4.062 (1.864) |  |
| Range | 1.100 - 8.100 | 0.600 - 9.000 | 1.300 - 11.500 | 0.400 - 9.350 | 0.400 - 8.900 | 0.450 - 10.000 | 0.400 - 11.500 |  |
| Fibrinogen variance |  |  |  |  |  |  |  | < 0.001 |
| N-Miss | 296 | 39 | 339 | 45 | 308 | 167 | 1194 |  |
| Mean (SD) | 0.311 (0.527) | 0.548 (0.590) | 0.206 (0.418) | 0.457 (0.616) | 0.327 (0.609) | 0.552 (0.680) | 0.378 (0.591) |  |
| Range | 0.000 - 2.666 | 0.000 - 2.626 | 0.000 - 2.350 | 0.000 - 3.500 | 0.000 - 3.350 | 0.000 - 3.755 | 0.000 - 3.755 |  |
| Phosphate mean |  |  |  |  |  |  |  | < 0.001 |
| N-Miss | 26 | 2 | 33 | 17 | 59 | 21 | 158 |  |
| Mean (SD) | 1.071 (0.285) | 1.462 (0.610) | 1.177 (0.415) | 1.069 (0.314) | 1.037 (0.265) | 1.251 (0.426) | 1.150 (0.390) |  |
| Range | 0.382 - 2.360 | 0.449 - 3.480 | 0.270 - 3.870 | 0.270 - 2.173 | 0.270 - 2.770 | 0.400 - 3.320 | 0.270 - 3.870 |  |
| Phosphate variance |  |  |  |  |  |  |  | < 0.001 |
| N-Miss | 26 | 2 | 33 | 17 | 59 | 21 | 158 |  |
| Mean (SD) | 0.202 (0.149) | 0.398 (0.274) | 0.188 (0.170) | 0.228 (0.195) | 0.177 (0.146) | 0.332 (0.227) | 0.234 (0.197) |  |
| Range | 0.000 - 1.225 | 0.000 - 1.808 | 0.000 - 0.906 | 0.000 - 0.975 | 0.000 - 1.155 | 0.000 - 1.570 | 0.000 - 1.808 |  |
| Gamma-GT mean |  |  |  |  |  |  |  | < 0.001 |
| N-Miss | 17 | 1 | 35 | 5 | 38 | 8 | 104 |  |
| Mean (SD) | 74.929 (69.830) | 387.148 (492.433) | 109.894 (108.461) | 75.220 (76.082) | 64.082 (75.754) | 157.620 (161.518) | 116.514 (174.898) |  |
| Range | 7.500 - 789.000 | 14.800 - 4794.500 | 6.750 - 833.000 | 5.500 - 699.333 | 6.000 - 924.000 | 6.667 - 1477.250 | 5.500 - 4794.500 |  |
| Gamma-GT variance |  |  |  |  |  |  |  | < 0.001 |
| N-Miss | 17 | 1 | 35 | 5 | 38 | 8 | 104 |  |
| Mean (SD) | 18.127 (26.768) | 160.871 (189.048) | 18.427 (31.125) | 21.778 (31.212) | 17.712 (37.679) | 59.086 (72.147) | 35.414 (71.852) |  |
| Range | 0.000 - 207.976 | 0.000 - 1186.909 | 0.000 - 287.858 | 0.000 - 223.734 | 0.000 - 438.579 | 0.000 - 523.559 | 0.000 - 1186.909 |  |
| Haemoglobin mean |  |  |  |  |  |  |  | < 0.001 |
| Mean (SD) | 7.283 (1.221) | 5.730 (0.920) | 5.970 (0.893) | 6.674 (1.055) | 7.157 (1.224) | 5.894 (0.887) | 6.525 (1.234) |  |
| Range | 4.582 - 11.850 | 4.116 - 9.189 | 4.050 - 9.900 | 4.434 - 10.215 | 4.471 - 11.400 | 4.114 - 10.270 | 4.050 - 11.850 |  |
| Haemoglobin variance |  |  |  |  |  |  |  | < 0.001 |
| Mean (SD) | 0.634 (0.300) | 0.641 (0.318) | 0.425 (0.241) | 0.938 (0.353) | 0.594 (0.318) | 0.670 (0.330) | 0.602 (0.328) |  |
| Range | 0.000 - 2.024 | 0.082 - 2.124 | 0.000 - 1.912 | 0.212 - 2.158 | 0.000 - 1.879 | 0.000 - 2.444 | 0.000 - 2.444 |  |
| Haematocrit mean |  |  |  |  |  |  |  | < 0.001 |
| N-Miss | 18 | 1 | 17 | 1 | 22 | 6 | 65 |  |
| Mean (SD) | 0.360 (0.059) | 0.281 (0.044) | 0.299 (0.044) | 0.330 (0.057) | 0.351 (0.060) | 0.293 (0.046) | 0.323 (0.060) |  |
| Range | 0.229 - 0.567 | 0.195 - 0.438 | 0.190 - 0.497 | 0.215 - 0.525 | 0.210 - 0.562 | 0.200 - 0.520 | 0.190 - 0.567 |  |
| Haematocrit variance |  |  |  |  |  |  |  | < 0.001 |
| N-Miss | 18 | 1 | 17 | 1 | 22 | 6 | 65 |  |
| Mean (SD) | 0.028 (0.017) | 0.028 (0.015) | 0.017 (0.013) | 0.044 (0.019) | 0.024 (0.017) | 0.030 (0.018) | 0.026 (0.017) |  |
| Range | 0.000 - 0.097 | 0.000 - 0.089 | 0.000 - 0.081 | 0.000 - 0.096 | 0.000 - 0.120 | 0.000 - 0.114 | 0.000 - 0.120 |  |
| Potassium mean |  |  |  |  |  |  |  | < 0.001 |
| N-Miss | 63 | 21 | 152 | 42 | 123 | 131 | 532 |  |
| Mean (SD) | 4.266 (0.570) | 4.458 (0.626) | 4.276 (0.606) | 4.355 (0.652) | 4.097 (0.481) | 4.423 (0.684) | 4.279 (0.605) |  |
| Range | 2.870 - 9.360 | 3.010 - 7.040 | 2.750 - 8.130 | 3.110 - 8.490 | 1.880 - 6.187 | 2.775 - 8.860 | 1.880 - 9.360 |  |
| Potassium variance |  |  |  |  |  |  |  | < 0.001 |
| N-Miss | 63 | 21 | 152 | 42 | 123 | 131 | 532 |  |
| Mean (SD) | 0.296 (0.222) | 0.503 (0.363) | 0.302 (0.285) | 0.315 (0.301) | 0.263 (0.270) | 0.455 (0.374) | 0.336 (0.307) |  |
| Range | 0.000 - 1.312 | 0.000 - 1.941 | 0.000 - 2.000 | 0.000 - 1.386 | 0.000 - 2.025 | 0.000 - 2.950 | 0.000 - 2.950 |  |
| Creatinine mean |  |  |  |  |  |  |  | < 0.001 |
| Mean (SD) | 98.143 (57.981) | 189.965 (180.295) | 142.376 (135.055) | 109.588 (55.918) | 78.078 (33.741) | 161.896 (119.136) | 123.417 (107.379) |  |
| Range | 19.923 - 590.000 | 17.250 - 1557.833 | 5.000 - 1017.333 | 14.385 - 559.143 | 14.000 - 364.000 | 27.333 - 1162.400 | 5.000 - 1557.833 |  |
| Creatinine variance |  |  |  |  |  |  |  | < 0.001 |
| Mean (SD) | 14.766 (15.753) | 54.524 (65.033) | 22.881 (32.765) | 21.771 (20.262) | 10.139 (11.357) | 43.215 (45.984) | 24.346 (35.178) |  |
| Range | 0.000 - 114.910 | 1.700 - 488.499 | 0.000 - 277.694 | 0.000 - 109.102 | 0.000 - 134.345 | 0.000 - 351.813 | 0.000 - 488.499 |  |
| LDH mean |  |  |  |  |  |  |  | < 0.001 |
| N-Miss | 27 | 1 | 33 | 0 | 43 | 4 | 108 |  |
| Mean (SD) | 347.461 (256.836) | 1328.983 (2085.324) | 305.072 (199.826) | 435.555 (339.225) | 288.608 (231.061) | 602.237 (642.063) | 442.081 (665.884) |  |
| Range | 88.500 - 3029.000 | 68.500 - 13633.400 | 81.333 - 3059.333 | 101.750 - 2719.833 | 100.500 - 4126.833 | 111.500 - 6409.000 | 68.500 - 13633.400 |  |
| LDH variance |  |  |  |  |  |  |  | < 0.001 |
| N-Miss | 27 | 1 | 33 | 0 | 43 | 4 | 108 |  |
| Mean (SD) | 72.662 (142.462) | 805.320 (1634.025) | 37.803 (62.243) | 134.901 (334.103) | 50.295 (134.465) | 265.200 (549.618) | 147.644 (518.665) |  |
| Range | 0.000 - 1687.500 | 0.000 - 7313.211 | 0.000 - 769.404 | 0.000 - 3393.486 | 0.000 - 3323.449 | 0.000 - 5106.921 | 0.000 - 7313.211 |  |
| Leukocytes mean |  |  |  |  |  |  |  | < 0.001 |
| N-Miss | 0 | 7 | 4 | 1 | 2 | 9 | 23 |  |
| Mean (SD) | 12.959 (5.124) | 17.120 (31.676) | 12.712 (6.410) | 12.876 (5.345) | 11.815 (4.092) | 13.565 (8.914) | 12.990 (9.672) |  |
| Range | 0.367 - 77.075 | 0.100 - 417.750 | 0.100 - 60.950 | 0.225 - 40.530 | 3.450 - 39.650 | 0.133 - 97.500 | 0.100 - 417.750 |  |
| Leukocytes variance |  |  |  |  |  |  |  | < 0.001 |
| N-Miss | 0 | 7 | 4 | 1 | 2 | 9 | 23 |  |
| Mean (SD) | 2.752 (1.859) | 6.315 (10.411) | 2.562 (2.302) | 3.590 (2.058) | 2.476 (1.773) | 4.318 (3.857) | 3.223 (3.614) |  |
| Range | 0.000 - 24.228 | 0.000 - 102.892 | 0.000 - 17.045 | 0.000 - 12.424 | 0.000 - 12.873 | 0.000 - 34.627 | 0.000 - 102.892 |  |
| Magnesium mean |  |  |  |  |  |  |  | < 0.001 |
| N-Miss | 91 | 6 | 127 | 30 | 151 | 51 | 456 |  |
| Mean (SD) | 0.835 (0.125) | 0.881 (0.182) | 0.818 (0.137) | 0.836 (0.154) | 0.802 (0.123) | 0.848 (0.168) | 0.830 (0.145) |  |
| Range | 0.437 - 1.390 | 0.500 - 2.170 | 0.420 - 1.625 | 0.343 - 1.490 | 0.400 - 2.110 | 0.400 - 2.686 | 0.343 - 2.686 |  |
| Magnesium variance |  |  |  |  |  |  |  | < 0.001 |
| N-Miss | 91 | 6 | 127 | 30 | 151 | 51 | 456 |  |
| Mean (SD) | 0.066 (0.063) | 0.093 (0.082) | 0.055 (0.056) | 0.083 (0.077) | 0.047 (0.048) | 0.089 (0.071) | 0.067 (0.065) |  |
| Range | 0.000 - 0.486 | 0.000 - 0.789 | 0.000 - 0.295 | 0.000 - 0.495 | 0.000 - 0.290 | 0.000 - 0.871 | 0.000 - 0.871 |  |
| Sodium mean |  |  |  |  |  |  |  | < 0.001 |
| N-Miss | 63 | 23 | 157 | 44 | 124 | 133 | 544 |  |
| Mean (SD) | 139.790 (4.181) | 142.128 (6.364) | 138.917 (5.464) | 140.617 (3.404) | 140.688 (4.424) | 142.208 (6.040) | 140.485 (5.208) |  |
| Range | 125.000 - 158.176 | 125.000 - 159.400 | 108.500 - 156.000 | 129.000 - 149.167 | 112.000 - 164.000 | 122.833 - 169.750 | 108.500 - 169.750 |  |
| Sodium variance |  |  |  |  |  |  |  | < 0.001 |
| N-Miss | 63 | 23 | 157 | 44 | 124 | 133 | 544 |  |
| Mean (SD) | 1.851 (1.627) | 3.364 (2.310) | 1.738 (1.811) | 1.775 (1.631) | 1.720 (1.866) | 2.953 (2.458) | 2.109 (2.041) |  |
| Range | 0.000 - 8.432 | 0.000 - 9.672 | 0.000 - 17.913 | 0.000 - 8.261 | 0.000 - 13.411 | 0.000 - 14.900 | 0.000 - 17.913 |  |
| Thrombocytes mean |  |  |  |  |  |  |  | < 0.001 |
| N-Miss | 0 | 0 | 0 | 0 | 0 | 2 | 2 |  |
| Mean (SD) | 236.699 (91.138) | 156.364 (119.737) | 284.848 (148.855) | 182.267 (81.149) | 233.247 (104.981) | 177.433 (109.266) | 226.759 (122.138) |  |
| Range | 13.882 - 595.364 | 13.333 - 479.364 | 15.000 - 1025.000 | 23.000 - 586.000 | 60.625 - 1580.000 | 12.545 - 751.271 | 12.545 - 1580.000 |  |
| Thrombocytes variance |  |  |  |  |  |  |  | < 0.001 |
| N-Miss | 0 | 0 | 0 | 0 | 0 | 2 | 2 |  |
| Mean (SD) | 44.443 (37.229) | 55.712 (55.290) | 41.286 (39.922) | 50.902 (44.282) | 47.797 (59.886) | 60.447 (55.375) | 48.900 (49.696) |  |
| Range | 0.000 - 283.337 | 3.709 - 342.047 | 0.000 - 309.382 | 0.000 - 226.330 | 0.000 - 571.822 | 0.000 - 391.646 | 0.000 - 571.822 |  |
| Urea mean |  |  |  |  |  |  |  | < 0.001 |
| N-Miss | 0 | 0 | 1 | 0 | 1 | 0 | 2 |  |
| Mean (SD) | 8.719 (4.576) | 17.202 (10.957) | 11.276 (8.073) | 8.764 (3.929) | 6.236 (3.341) | 14.069 (8.523) | 10.374 (7.460) |  |
| Range | 1.740 - 32.500 | 1.525 - 71.050 | 1.000 - 48.033 | 2.062 - 25.911 | 1.367 - 36.837 | 1.750 - 72.300 | 1.000 - 72.300 |  |
| Urea variance |  |  |  |  |  |  |  | < 0.001 |
| N-Miss | 0 | 0 | 1 | 0 | 1 | 0 | 2 |  |
| Mean (SD) | 1.655 (1.464) | 5.144 (4.334) | 2.035 (2.409) | 2.019 (1.741) | 1.115 (1.095) | 3.666 (3.250) | 2.268 (2.616) |  |
| Range | 0.000 - 10.155 | 0.100 - 24.404 | 0.000 - 16.342 | 0.000 - 14.190 | 0.000 - 8.461 | 0.000 - 19.307 | 0.000 - 24.404 |  |
| BMI |  |  |  |  |  |  |  | < 0.001 |
| N-Miss | 63 | 16 | 123 | 12 | 100 | 70 | 384 |  |
| Mean (SD) | 26.756 (5.013) | 25.261 (4.721) | 26.441 (6.553) | 27.555 (6.393) | 25.846 (4.846) | 25.984 (5.120) | 26.279 (5.488) |  |
| Range | 14.800 - 48.400 | 13.800 - 51.900 | 11.400 - 82.700 | 15.600 - 62.400 | 13.800 - 58.600 | 13.100 - 57.400 | 11.400 - 82.700 |  |
| Previous ICU admission |  |  |  |  |  |  |  | < 0.001 |
| No | 265 (83.1%) | 1210 (93.6%) | 342 (89.3%) | 569 (86.3%) | 372 (80.3%) | 713 (91.8%) | 3471 (89.1%) |  |
| Yes | 54 (16.9%) | 83 (6.4%) | 41 (10.7%) | 90 (13.7%) | 91 (19.7%) | 64 (8.2%) | 423 (10.9%) |  |
| Systolic blood pressure |  |  |  |  |  |  |  | < 0.001 |
| N-Miss | 0 | 0 | 0 | 0 | 1 | 0 | 1 |  |
| Mean (SD) | 140.557 (33.137) | 111.699 (29.557) | 127.753 (32.410) | 109.797 (26.368) | 131.981 (32.597) | 109.053 (28.977) | 125.354 (33.556) |  |
| Range | 57.000 - 313.000 | 45.000 - 228.000 | 55.000 - 305.000 | 54.000 - 211.000 | 43.000 - 265.000 | 0.000 - 228.000 | 0.000 - 313.000 |  |
| Diastolic blood pressure |  |  |  |  |  |  |  | < 0.001 |
| N-Miss | 0 | 0 | 0 | 0 | 1 | 0 | 1 |  |
| Mean (SD) | 76.101 (18.069) | 59.306 (17.538) | 66.064 (21.198) | 58.788 (15.803) | 69.861 (19.817) | 56.735 (17.347) | 66.177 (20.179) |  |
| Range | 17.000 - 160.000 | 15.000 - 105.000 | 18.000 - 172.000 | 20.000 - 119.000 | 20.000 - 196.000 | 0.000 - 141.000 | 0.000 - 196.000 |  |
| Mean arterial pressure |  |  |  |  |  |  |  | < 0.001 |
| N-Miss | 0 | 0 | 0 | 0 | 1 | 0 | 1 |  |
| Mean (SD) | 76.101 (18.069) | 59.306 (17.538) | 66.064 (21.198) | 58.788 (15.803) | 69.861 (19.817) | 56.735 (17.347) | 66.177 (20.179) |  |
| Range | 17.000 - 160.000 | 15.000 - 105.000 | 18.000 - 172.000 | 20.000 - 119.000 | 20.000 - 196.000 | 0.000 - 141.000 | 0.000 - 196.000 |  |
| Atrial fibrillation |  |  |  |  |  |  |  | < 0.001 |
| No | 281 (88.1%) | 1151 (89.0%) | 354 (92.4%) | 612 (92.9%) | 408 (88.1%) | 696 (89.6%) | 3502 (89.9%) |  |
| Yes | 38 (11.9%) | 142 (11.0%) | 29 (7.6%) | 47 (7.1%) | 55 (11.9%) | 81 (10.4%) | 392 (10.1%) |  |
| Heart rate at admission |  |  |  |  |  |  |  | < 0.001 |
| Mean (SD) | 93.644 (24.630) | 110.205 (26.542) | 100.128 (23.845) | 86.656 (21.136) | 83.307 (23.004) | 103.356 (26.330) | 95.379 (25.751) |  |
| Range | 41.000 - 195.000 | 52.000 - 203.000 | 35.000 - 213.000 | 45.000 - 164.000 | 1.000 - 215.000 | 41.000 - 250.000 | 1.000 - 250.000 |  |
| Urine output in previous 6 hours |  |  |  |  |  |  |  | < 0.001 |
| N-Miss | 28 | 9 | 44 | 4 | 29 | 29 | 143 |  |
| Mean (SD) | 0.613 (0.557) | 0.383 (0.405) | 0.565 (0.574) | 0.467 (0.462) | 0.633 (0.530) | 0.456 (0.701) | 0.551 (0.582) |  |
| Range | 0.000 - 4.060 | 0.000 - 2.500 | 0.000 - 6.100 | 0.000 - 3.900 | 0.000 - 6.700 | 0.000 - 12.000 | 0.000 - 12.000 |  |
| Central venous pressure |  |  |  |  |  |  |  | < 0.001 |
| No | 290 (90.9%) | 1111 (85.9%) | 296 (77.3%) | 644 (97.7%) | 432 (93.3%) | 588 (75.7%) | 3361 (86.3%) |  |
| Yes | 29 (9.1%) | 182 (14.1%) | 87 (22.7%) | 15 (2.3%) | 31 (6.7%) | 189 (24.3%) | 533 (13.7%) |  |
| Worsened respiratory condition |  |  |  |  |  |  |  | < 0.001 |
| No | 231 (72.4%) | 881 (68.1%) | 281 (73.4%) | 520 (78.9%) | 371 (80.1%) | 450 (57.9%) | 2734 (70.2%) |  |
| Yes | 88 (27.6%) | 412 (31.9%) | 102 (26.6%) | 139 (21.1%) | 92 (19.9%) | 327 (42.1%) | 1160 (29.8%) |  |
| Tidal volume |  |  |  |  |  |  |  | < 0.001 |
| N-Miss | 217 | 64 | 398 | 22 | 180 | 184 | 1065 |  |
| Mean (SD) | 482.401 (159.733) | 490.219 (150.194) | 453.373 (175.846) | 511.872 (129.220) | 491.484 (166.315) | 483.507 (154.789) | 482.321 (161.387) |  |
| Range | 0.000 - 1347.000 | 58.000 - 1280.000 | 0.000 - 1100.000 | 0.000 - 935.000 | 0.000 - 1919.000 | 0.000 - 1410.000 | 0.000 - 1919.000 |  |
| Respiratory rate |  |  |  |  |  |  |  | < 0.001 |
| N-Miss | 305 | 74 | 531 | 28 | 238 | 236 | 1412 |  |
| Mean (SD) | 16.438 (2.896) | 20.117 (6.544) | 17.501 (4.092) | 16.958 (3.115) | 15.965 (2.668) | 17.875 (3.878) | 17.082 (3.763) |  |
| Range | 9.000 - 30.000 | 12.000 - 70.000 | 10.000 - 40.000 | 12.000 - 33.000 | 4.000 - 26.000 | 5.000 - 30.000 | 4.000 - 70.000 |  |
| Positive end-expiratory pressure |  |  |  |  |  |  |  | < 0.001 |
| N-Miss | 5 | 0 | 2 | 2 | 2 | 3 | 14 |  |
| Mean (SD) | 6.706 (1.967) | 8.479 (3.635) | 6.982 (2.542) | 8.255 (2.371) | 6.604 (1.936) | 7.856 (2.848) | 7.188 (2.525) |  |
| Range | 0.000 - 14.000 | 5.000 - 23.000 | 4.000 - 20.000 | 4.000 - 18.000 | 0.000 - 16.000 | 0.000 - 24.000 | 0.000 - 24.000 |  |
| Mechanical ventilation after 24h |  |  |  |  |  |  |  | < 0.001 |
| No | 193 (60.5%) | 367 (28.4%) | 220 (57.4%) | 476 (72.2%) | 313 (67.6%) | 212 (27.3%) | 1781 (45.7%) |  |
| Yes | 126 (39.5%) | 926 (71.6%) | 163 (42.6%) | 183 (27.8%) | 150 (32.4%) | 565 (72.7%) | 2113 (54.3%) |  |
| Mechanical ventilation at admission |  |  |  |  |  |  |  | < 0.001 |
| No | 264 (82.8%) | 571 (44.2%) | 273 (71.3%) | 572 (86.8%) | 374 (80.8%) | 514 (66.2%) | 2568 (65.9%) |  |
| Yes | 55 (17.2%) | 722 (55.8%) | 110 (28.7%) | 87 (13.2%) | 89 (19.2%) | 263 (33.8%) | 1326 (34.1%) |  |
| Respiratory rate |  |  |  |  |  |  |  | < 0.001 |
| Mean (SD) | 19.617 (5.849) | 26.470 (15.260) | 23.190 (8.506) | 17.656 (4.662) | 17.440 (4.774) | 22.689 (9.443) | 20.879 (8.364) |  |
| Range | 0.000 - 42.000 | 10.000 - 120.000 | 0.000 - 95.000 | 0.000 - 38.000 | 0.000 - 41.000 | 0.000 - 100.000 | 0.000 - 120.000 |  |
| FiO2 low |  |  |  |  |  |  |  | < 0.001 |
| N-Miss | 154 | 37 | 324 | 11 | 148 | 113 | 787 |  |
| Mean (SD) | 31.440 (9.162) | 32.599 (11.704) | 33.970 (11.477) | 32.326 (9.209) | 29.676 (7.140) | 31.760 (10.071) | 31.709 (9.710) |  |
| Range | 21.000 - 100.000 | 21.000 - 80.000 | 21.000 - 100.000 | 21.000 - 100.000 | 21.000 - 100.000 | 21.000 - 100.000 | 21.000 - 100.000 |  |
| Myocardial infarction (history) |  |  |  |  |  |  |  | < 0.001 |
| No | 297 (93.1%) | 1122 (86.8%) | 336 (87.7%) | 610 (92.6%) | 429 (92.7%) | 702 (90.3%) | 3496 (89.8%) |  |
| Yes | 22 (6.9%) | 171 (13.2%) | 47 (12.3%) | 49 (7.4%) | 34 (7.3%) | 75 (9.7%) | 398 (10.2%) |  |
| Diabetes (history) |  |  |  |  |  |  |  | < 0.001 |
| No | 238 (74.6%) | 1182 (91.4%) | 339 (88.5%) | 584 (88.6%) | 404 (87.3%) | 686 (88.3%) | 3433 (88.2%) |  |
| Yes | 81 (25.4%) | 111 (8.6%) | 44 (11.5%) | 75 (11.4%) | 59 (12.7%) | 91 (11.7%) | 461 (11.8%) |  |
| Cardiovascular disease (history) |  |  |  |  |  |  |  | < 0.001 |
| Mean (SD) | 277 (86.8%) | 1226 (94.8%) | 354 (92.4%) | 614 (93.2%) | 438 (94.6%) | 752 (96.8%) | 3661 (94.0%) |  |
| Range | 42 (13.2%) | 67 (5.2%) | 29 (7.6%) | 45 (6.8%) | 25 (5.4%) | 25 (3.2%) | 233 (6.0%) |  |
| Chronic Obstructive pulmonary disease (history) |  |  |  |  |  |  |  | < 0.001 |
| No | 286 (89.7%) | 1192 (92.2%) | 347 (90.6%) | 541 (82.1%) | 395 (85.3%) | 710 (91.4%) | 3471 (89.1%) |  |
| Yes | 33 (10.3%) | 101 (7.8%) | 36 (9.4%) | 118 (17.9%) | 68 (14.7%) | 67 (8.6%) | 423 (10.9%) |  |
| Respiratory insufficiency (history) |  |  |  |  |  |  |  | < 0.001 |
| No | 307 (96.2%) | 1254 (97.0%) | 369 (96.3%) | 601 (91.2%) | 437 (94.4%) | 765 (98.5%) | 3733 (95.9%) |  |
| Yes | 12 (3.8%) | 39 (3.0%) | 14 (3.7%) | 58 (8.8%) | 26 (5.6%) | 12 (1.5%) | 161 (4.1%) |  |
| Chronic kidney disease (history) |  |  |  |  |  |  |  | NaN |
| No | 319 (100.0%) | 1293 (100.0%) | 383 (100.0%) | 659 (100.0%) | 463 (100.0%) | 777 (100.0%) | 3894 (100.0%) |  |
| Yes | 0 (0.0%) | 0 (0.0%) | 0 (0.0%) | 0 (0.0%) | 0 (100.0%) | 0 (0.0%) | 0 (0.0%) |  |
| Dialysis (history) |  |  |  |  |  |  |  | < 0.001 |
| No | 277 (86.8%) | 1288 (99.6%) | 379 (99.0%) | 658 (99.8%) | 455 (98.3%) | 760 (97.8%) | 3817 (98.0%) |  |
| Yes | 42 (13.2%) | 5 (0.4%) | 4 (1.0%) | 1 (0.2%) | 8 (1.7%) | 17 (2.2%) | 77 (2.0%) |  |
| Cirrhosis (history) |  |  |  |  |  |  |  | 0.004 |
| No | 309 (96.9%) | 1266 (97.9%) | 378 (98.7%) | 651 (98.8%) | 458 (98.9%) | 741 (95.4%) | 3803 (97.7%) |  |
| Yes | 10 (3.1%) | 27 (2.1%) | 5 (1.3%) | 8 (1.2%) | 5 (1.1%) | 36 (4.6%) | 91 (2.3%) |  |
| Metastatic disease (history) |  |  |  |  |  |  |  | < 0.001 |
| No | 302 (94.7%) | 1245 (96.3%) | 357 (93.2%) | 632 (95.9%) | 426 (92.0%) | 728 (93.7%) | 3690 (94.8%) |  |
| Yes | 17 (5.3%) | 48 (3.7%) | 26 (6.8%) | 27 (4.1%) | 37 (8.0%) | 49 (6.3%) | 204 (5.2%) |  |
| Haematological malignancy (history) |  |  |  |  |  |  |  | < 0.001 |
| No | 283 (88.7%) | 1266 (97.9%) | 360 (94.0%) | 630 (95.6%) | 433 (93.5%) | 696 (89.6%) | 3668 (94.2%) |  |
| Yes | 36 (11.3%) | 27 (2.1%) | 23 (6.0%) | 29 (4.4%) | 30 (6.5%) | 81 (10.4%) | 226 (5.8%) |  |
| Immune insufficiency (history) |  |  |  |  |  |  |  | < 0.001 |
| No | 273 (85.6%) | 1240 (95.9%) | 347 (90.6%) | 613 (93.0%) | 417 (90.1%) | 660 (84.9%) | 3550 (91.2%) |  |
| Yes | 46 (14.4%) | 53 (4.1%) | 36 (9.4%) | 46 (7.0%) | 46 (9.9%) | 117 (15.1%) | 344 (8.8%) |  |
