# Supplementary material for: Deep embedded clustering generalisability and adaptation for integrating mixed datatypes: two critical care cohorts
Source: Sci Rep. 2024 Jan 10;14:1045. doi: 10.1038/s41598-024-51699-z (PMC10781731; doi:10.1038/s41598-024-51699-z)
Supplement: Supplementary file 5 — Supplementary Table S6. [file 41598_2024_51699_MOESM5_ESM.docx]

**Table S6. Descriptive statistics of the input and outcome variables for the X-DEC clusters on the SICS dataset.** The first column specifies the variables (in bold), and whether the variables is described by its mean with standard deviation (SD) and the range of values, or if it is a category, the number of patients in each level, and how much percentage of the patients fall in that level. If the variable is missing for some samples, this is indicated by ‘N-miss’, which specifies the number of samples for which this variable was missing. The other columns specify the different clusters, and complete dataset. The last column specifies the p-value. The Chi-square test was used for categorical variables, and the Kruskal-Wallis Rank Test for numeric variables.

|  | Cluster 1 (N=61) | Cluster 2 (N=200) | Cluster 3 (N=149) | Cluster 4 (N=158) | Cluster 5 (N=132) | Cluster 6 (N=87) | Total (N=787) | p value |
| --- | --- | --- | --- | --- | --- | --- | --- | --- |
| In-ICU mortality |  |  |  |  |  |  |  | < 0.001 |
| Survivor | 38 (62.3%) | 149 (74.5%) | 127 (85.2%) | 150 (94.9%) | 111 (84.1%) | 66 (75.9%) | 641 (81.4%) |  |
| Non-survivor | 23 (37.7%) | 51 (25.5%) | 22 (14.8%) | 8 (5.1%) | 21 (15.9%) | 21 (24.1%) | 146 (18.6%) |  |
| Length of stay |  |  |  |  |  |  |  | < 0.001 |
| Mean (SD) | 7.720 (11.481) | 6.126 (7.305) | 6.963 (8.851) | 3.802 (3.833) | 5.239 (7.108) | 7.289 (7.657) | 5.921 (7.599) |  |
| Range | 1.000 - 83.651 | 1.023 - 70.924 | 1.073 - 50.567 | 1.054 - 26.990 | 1.012 - 50.140 | 1.022 - 33.719 | 1.000 - 83.651 |  |
| Apache IV mortality |  |  |  |  |  |  |  | < 0.001 |
| N-Miss | 5 | 9 | 31 | 25 | 22 | 17 | 109 |  |
| Mean (SD) | 0.453 (0.266) | 0.493 (0.242) | 0.220 (0.201) | 0.155 (0.133) | 0.277 (0.214) | 0.286 (0.276) | 0.319 (0.255) |  |
| Range | 0.033 - 0.975 | 0.014 - 0.981 | 0.001 - 0.803 | 0.007 - 0.778 | 0.006 - 0.892 | 0.004 - 0.950 | 0.001 - 0.981 |  |
| Apache IV score |  |  |  |  |  |  |  | < 0.001 |
| N-Miss | 5 | 9 | 31 | 25 | 22 | 17 | 109 |  |
| Mean (SD) | 94.375 (25.463) | 88.670 (25.814) | 71.237 (24.620) | 53.737 (17.030) | 73.245 (23.349) | 83.729 (34.569) | 76.242 (28.151) |  |
| Range | 45.000 - 175.000 | 24.000 - 216.000 | 21.000 - 140.000 | 17.000 - 96.000 | 17.000 - 161.000 | 37.000 - 194.000 | 17.000 - 216.000 |  |
| SAPS II score |  |  |  |  |  |  |  | < 0.001 |
| Mean (SD) | 54.000 (13.988) | 58.230 (13.328) | 42.550 (12.570) | 33.563 (11.014) | 42.182 (14.311) | 50.345 (18.276) | 46.418 (16.288) |  |
| Range | 23.000 - 94.000 | 28.000 - 99.000 | 6.000 - 73.000 | 13.000 - 72.000 | 6.000 - 91.000 | 26.000 - 111.000 | 6.000 - 111.000 |  |
| Postoperative (based on APACHE IV) |  |  |  |  |  |  |  | < 0.001 |
| No | 50 (82.0%) | 147 (73.5%) | 66 (44.3%) | 116 (73.4%) | 100 (75.8%) | 41 (47.1%) | 520 (66.1%) |  |
| Yes | 11 (18.0%) | 53 (26.5%) | 83 (55.7%) | 42 (26.6%) | 32 (24.2%) | 46 (52.9%) | 267 (33.9%) |  |
| Admission diagnosis (based on APACHE IV) |  |  |  |  |  |  |  | < 0.001 |
|  | 0 (0.0%) | 0 (0.0%) | 0 (0.0%) | 0 (0.0%) | 1 (0.8%) | 0 (0.0%) | 1 (0.1%) |  |
| Cardiovascular | 17 (27.9%) | 84 (42.0%) | 55 (36.9%) | 43 (27.2%) | 28 (21.2%) | 24 (27.6%) | 251 (31.9%) |  |
| Gastrointestinal | 14 (23.0%) | 7 (3.5%) | 29 (19.5%) | 8 (5.1%) | 31 (23.5%) | 19 (21.8%) | 108 (13.7%) |  |
| Genito-urinary | 3 (4.9%) | 1 (0.5%) | 3 (2.0%) | 1 (0.6%) | 3 (2.3%) | 2 (2.3%) | 13 (1.7%) |  |
| Haematological | 3 (4.9%) | 0 (0.0%) | 2 (1.3%) | 0 (0.0%) | 3 (2.3%) | 2 (2.3%) | 10 (1.3%) |  |
| Metabolic | 1 (1.6%) | 1 (0.5%) | 1 (0.7%) | 1 (0.6%) | 11 (8.3%) | 3 (3.4%) | 18 (2.3%) |  |
| Musculoskeletal/skin | 1 (1.6%) | 0 (0.0%) | 3 (2.0%) | 0 (0.0%) | 4 (3.0%) | 0 (0.0%) | 8 (1.0%) |  |
| Neurological | 0 (0.0%) | 70 (35.0%) | 7 (4.7%) | 40 (25.3%) | 3 (2.3%) | 3 (3.4%) | 123 (15.6%) |  |
| Respiratory | 21 (34.4%) | 9 (4.5%) | 28 (18.8%) | 51 (32.3%) | 42 (31.8%) | 9 (10.3%) | 160 (20.3%) |  |
| Transplant | 1 (1.6%) | 0 (0.0%) | 13 (8.7%) | 1 (0.6%) | 4 (3.0%) | 19 (21.8%) | 38 (4.8%) |  |
| Trauma | 0 (0.0%) | 28 (14.0%) | 8 (5.4%) | 13 (8.2%) | 2 (1.5%) | 6 (6.9%) | 57 (7.2%) |  |
| Age |  |  |  |  |  |  |  | < 0.001 |
| Mean (SD) | 67.033 (10.844) | 62.315 (14.222) | 61.013 (14.158) | 60.405 (13.968) | 65.682 (13.471) | 57.931 (15.653) | 62.131 (14.168) |  |
| Range | 33.000 - 87.000 | 21.000 - 94.000 | 20.000 - 89.000 | 18.000 - 89.000 | 20.000 - 94.000 | 21.000 - 89.000 | 18.000 - 94.000 |  |
| gender |  |  |  |  |  |  |  | 0.148 |
| Female | 18 (29.5%) | 65 (32.5%) | 54 (36.2%) | 57 (36.1%) | 58 (43.9%) | 39 (44.8%) | 291 (37.0%) |  |
| Male | 43 (70.5%) | 135 (67.5%) | 95 (63.8%) | 101 (63.9%) | 74 (56.1%) | 48 (55.2%) | 496 (63.0%) |  |
| vasoactive |  |  |  |  |  |  |  | < 0.001 |
| No | 28 (45.9%) | 105 (52.5%) | 50 (33.6%) | 127 (80.4%) | 70 (53.0%) | 23 (26.4%) | 403 (51.2%) |  |
| Yes | 33 (54.1%) | 95 (47.5%) | 99 (66.4%) | 31 (19.6%) | 62 (47.0%) | 64 (73.6%) | 384 (48.8%) |  |
| Renal replacement therapy |  |  |  |  |  |  |  | < 0.001 |
| No | 47 (77.0%) | 199 (99.5%) | 143 (96.0%) | 157 (99.4%) | 131 (99.2%) | 85 (97.7%) | 762 (96.8%) |  |
| Yes | 14 (23.0%) | 1 (0.5%) | 6 (4.0%) | 1 (0.6%) | 1 (0.8%) | 2 (2.3%) | 25 (3.2%) |  |
| NOR |  |  |  |  |  |  |  | < 0.001 |
| No | 28 (45.9%) | 107 (53.5%) | 54 (36.2%) | 129 (81.6%) | 72 (54.5%) | 23 (26.4%) | 413 (52.5%) |  |
| Yes | 33 (54.1%) | 93 (46.5%) | 95 (63.8%) | 29 (18.4%) | 60 (45.5%) | 64 (73.6%) | 374 (47.5%) |  |
| ICU readmission |  |  |  |  |  |  |  | 0.009 |
| 1st readmission | 5 (8.2%) | 9 (4.5%) | 17 (11.4%) | 22 (13.9%) | 17 (12.9%) | 8 (9.2%) | 78 (9.9%) |  |
| 2nd readmission | 0 (0.0%) | 0 (0.0%) | 3 (2.0%) | 2 (1.3%) | 1 (0.8%) | 4 (4.6%) | 10 (1.3%) |  |
| no readmission | 56 (91.8%) | 191 (95.5%) | 129 (86.6%) | 134 (84.8%) | 114 (86.4%) | 75 (86.2%) | 699 (88.8%) |  |
| EMV score |  |  |  |  |  |  |  | < 0.001 |
| Mean (SD) | 13.295 (3.671) | 4.095 (2.056) | 14.383 (2.130) | 13.449 (2.983) | 14.242 (2.348) | 12.931 (4.294) | 11.313 (5.066) |  |
| Range | 3.000 - 15.000 | 2.000 - 12.000 | 3.000 - 15.000 | 3.000 - 15.000 | 3.000 - 15.000 | 3.000 - 15.000 | 2.000 - 15.000 |  |
| Temperature center |  |  |  |  |  |  |  | 0.155 |
| N-Miss | 1 | 0 | 1 | 6 | 0 | 1 | 9 |  |
| Mean (SD) | 36.867 (1.077) | 36.819 (1.006) | 36.995 (0.865) | 37.016 (0.698) | 37.061 (0.843) | 36.931 (0.939) | 36.948 (0.899) |  |
| Range | 35.100 - 40.200 | 33.400 - 39.500 | 34.800 - 40.100 | 35.000 - 39.900 | 35.000 - 39.500 | 34.900 - 40.100 | 33.400 - 40.200 |  |
| Admission type |  |  |  |  |  |  |  | < 0.001 |
| Acute surgery | 9 (14.8%) | 57 (28.5%) | 70 (47.0%) | 41 (25.9%) | 25 (18.9%) | 42 (48.3%) | 244 (31.0%) |  |
| Medical | 51 (83.6%) | 143 (71.5%) | 65 (43.6%) | 114 (72.2%) | 101 (76.5%) | 40 (46.0%) | 514 (65.3%) |  |
| Planned surgery | 1 (1.6%) | 0 (0.0%) | 14 (9.4%) | 3 (1.9%) | 6 (4.5%) | 5 (5.7%) | 29 (3.7%) |  |
| ALAT mean |  |  |  |  |  |  |  | < 0.001 |
| N-Miss | 0 | 1 | 0 | 5 | 2 | 0 | 8 |  |
| Mean (SD) | 85.958 (113.853) | 88.199 (155.957) | 57.309 (86.084) | 53.046 (111.615) | 59.172 (126.654) | 553.482 (833.922) | 122.331 (337.898) |  |
| Range | 6.400 - 654.833 | 5.000 - 1463.333 | 6.333 - 541.833 | 5.500 - 1075.750 | 6.000 - 987.750 | 5.500 - 5141.200 | 5.000 - 5141.200 |  |
| ALAT variance |  |  |  |  |  |  |  | < 0.001 |
| N-Miss | 0 | 1 | 0 | 5 | 2 | 0 | 8 |  |
| Mean (SD) | 33.616 (77.949) | 20.561 (27.459) | 24.160 (53.909) | 10.830 (32.620) | 15.050 (45.940) | 390.293 (710.294) | 60.733 (266.928) |  |
| Range | 0.000 - 509.976 | 0.000 - 146.261 | 0.000 - 401.567 | 0.000 - 302.907 | 0.000 - 447.327 | 0.000 - 5065.483 | 0.000 - 5065.483 |  |
| ASAT mean |  |  |  |  |  |  |  | < 0.001 |
| N-Miss | 0 | 1 | 0 | 5 | 2 | 0 | 8 |  |
| Mean (SD) | 140.057 (212.613) | 114.015 (166.525) | 101.189 (126.829) | 54.709 (77.807) | 68.705 (105.841) | 938.152 (1465.558) | 186.432 (570.866) |  |
| Range | 11.538 - 1241.667 | 11.667 - 1460.667 | 10.333 - 710.154 | 11.500 - 562.750 | 10.818 - 881.000 | 10.500 - 9648.600 | 10.333 - 9648.600 |  |
| ASAT variance |  |  |  |  |  |  |  | < 0.001 |
| N-Miss | 0 | 1 | 0 | 5 | 2 | 0 | 8 |  |
| Mean (SD) | 67.888 (153.778) | 37.747 (57.794) | 52.922 (104.044) | 16.502 (46.109) | 22.588 (54.409) | 825.794 (1503.381) | 124.318 (563.614) |  |
| Range | 0.000 - 914.903 | 0.000 - 338.690 | 0.000 - 896.568 | 0.000 - 370.640 | 0.000 - 426.171 | 0.000 - 10628.866 | 0.000 - 10628.866 |  |
| Albumin mean |  |  |  |  |  |  |  | < 0.001 |
| N-Miss | 0 | 3 | 0 | 13 | 4 | 0 | 20 |  |
| Mean (SD) | 27.392 (5.467) | 32.918 (4.510) | 25.977 (5.269) | 35.034 (5.469) | 27.065 (5.344) | 25.061 (4.923) | 29.662 (6.384) |  |
| Range | 16.500 - 40.000 | 17.909 - 43.000 | 12.938 - 36.667 | 21.500 - 46.500 | 14.667 - 39.750 | 17.200 - 41.333 | 12.938 - 46.500 |  |
| Albumin variance |  |  |  |  |  |  |  | < 0.001 |
| N-Miss | 0 | 3 | 0 | 13 | 4 | 0 | 20 |  |
| Mean (SD) | 1.877 (1.216) | 1.889 (1.535) | 2.826 (2.029) | 1.341 (1.416) | 1.761 (1.245) | 3.697 (2.516) | 2.150 (1.842) |  |
| Range | 0.000 - 4.710 | 0.000 - 7.106 | 0.000 - 10.677 | 0.000 - 6.000 | 0.000 - 4.989 | 0.000 - 10.873 | 0.000 - 10.873 |  |
| ALP mean |  |  |  |  |  |  |  | < 0.001 |
| N-Miss | 0 | 1 | 0 | 10 | 2 | 0 | 13 |  |
| Mean (SD) | 208.068 (495.489) | 77.946 (35.967) | 79.193 (65.161) | 78.790 (34.898) | 127.781 (108.942) | 124.701 (102.701) | 102.228 (158.102) |  |
| Range | 36.000 - 3860.000 | 26.500 - 307.429 | 29.333 - 502.364 | 24.667 - 280.500 | 29.500 - 630.143 | 18.333 - 654.667 | 18.333 - 3860.000 |  |
| ALP variance |  |  |  |  |  |  |  | < 0.001 |
| N-Miss | 0 | 1 | 0 | 10 | 2 | 0 | 13 |  |
| Mean (SD) | 38.491 (94.290) | 12.088 (18.953) | 19.155 (23.962) | 5.663 (7.126) | 16.631 (30.811) | 41.864 (53.080) | 18.410 (38.850) |  |
| Range | 0.000 - 643.456 | 0.000 - 146.229 | 0.000 - 148.139 | 0.000 - 41.427 | 0.000 - 270.241 | 0.000 - 278.373 | 0.000 - 643.456 |  |
| Bilirubin (total) mean |  |  |  |  |  |  |  | < 0.001 |
| N-Miss | 0 | 1 | 0 | 11 | 2 | 0 | 14 |  |
| Mean (SD) | 39.859 (93.516) | 9.912 (7.145) | 13.564 (18.850) | 10.225 (9.369) | 11.871 (9.943) | 43.773 (81.590) | 17.179 (41.061) |  |
| Range | 3.000 - 528.750 | 3.000 - 54.250 | 3.000 - 188.000 | 3.000 - 103.667 | 3.000 - 73.000 | 3.500 - 554.714 | 3.000 - 554.714 |  |
| Bilirubin (total) variance |  |  |  |  |  |  |  | < 0.001 |
| N-Miss | 0 | 1 | 0 | 11 | 2 | 0 | 14 |  |
| Mean (SD) | 6.048 (9.745) | 2.464 (4.427) | 3.975 (5.041) | 1.300 (1.840) | 2.541 (2.956) | 15.499 (34.589) | 4.297 (13.044) |  |
| Range | 0.000 - 64.210 | 0.000 - 48.085 | 0.000 - 39.543 | 0.000 - 14.091 | 0.000 - 19.221 | 0.000 - 202.583 | 0.000 - 202.583 |  |
| CK mean |  |  |  |  |  |  |  | < 0.001 |
| Mean (SD) | 474.593 (982.655) | 791.418 (1306.045) | 936.652 (2302.451) | 535.897 (3007.375) | 393.956 (823.714) | 2868.355 (10074.202) | 905.992 (3878.614) |  |
| Range | 8.333 - 5398.727 | 15.000 - 8126.500 | 12.000 - 21362.385 | 14.500 - 37659.833 | 7.000 - 6653.200 | 20.800 - 68697.167 | 7.000 - 68697.167 |  |
| CK variance |  |  |  |  |  |  |  | < 0.001 |
| Mean (SD) | 270.274 (698.327) | 447.790 (800.040) | 453.247 (946.027) | 172.564 (817.421) | 183.935 (471.555) | 1703.338 (5478.751) | 474.350 (2006.426) |  |
| Range | 0.000 - 3879.205 | 0.000 - 4584.077 | 0.000 - 9478.092 | 0.000 - 9996.416 | 0.000 - 4624.583 | 0.000 - 39751.232 | 0.000 - 39751.232 |  |
| CRP mean |  |  |  |  |  |  |  | < 0.001 |
| Mean (SD) | 136.867 (96.614) | 65.863 (55.530) | 97.217 (76.193) | 55.740 (71.576) | 172.006 (96.098) | 78.346 (69.855) | 94.453 (85.859) |  |
| Range | 12.429 - 444.600 | 0.750 - 276.667 | 1.450 - 360.500 | 0.300 - 361.000 | 14.275 - 438.250 | 1.933 - 310.333 | 0.300 - 444.600 |  |
| CRP variance |  |  |  |  |  |  |  | < 0.001 |
| Mean (SD) | 48.128 (37.647) | 42.623 (30.076) | 53.154 (35.272) | 21.757 (22.620) | 62.753 (42.183) | 39.223 (34.531) | 43.855 (35.769) |  |
| Range | 0.500 - 151.261 | 0.000 - 139.953 | 0.250 - 152.285 | 0.000 - 110.186 | 0.000 - 194.114 | 0.000 - 157.921 | 0.000 - 194.114 |  |
| Calcium mean |  |  |  |  |  |  |  | < 0.001 |
| N-Miss | 0 | 3 | 0 | 11 | 3 | 0 | 17 |  |
| Mean (SD) | 2.102 (0.211) | 2.080 (0.108) | 1.952 (0.119) | 2.152 (0.144) | 2.049 (0.151) | 1.940 (0.159) | 2.050 (0.159) |  |
| Range | 1.584 - 2.933 | 1.750 - 2.373 | 1.630 - 2.493 | 1.590 - 2.905 | 1.630 - 2.660 | 1.608 - 2.280 | 1.584 - 2.933 |  |
| Calcium variance |  |  |  |  |  |  |  | < 0.001 |
| N-Miss | 0 | 3 | 0 | 11 | 3 | 0 | 17 |  |
| Mean (SD) | 0.112 (0.089) | 0.059 (0.045) | 0.084 (0.059) | 0.039 (0.043) | 0.062 (0.042) | 0.120 (0.082) | 0.072 (0.062) |  |
| Range | 0.000 - 0.479 | 0.000 - 0.242 | 0.000 - 0.280 | 0.000 - 0.178 | 0.000 - 0.162 | 0.000 - 0.383 | 0.000 - 0.479 |  |
| Chloride mean |  |  |  |  |  |  |  | < 0.001 |
| N-Miss | 0 | 0 | 0 | 1 | 0 | 0 | 1 |  |
| Mean (SD) | 102.097 (5.977) | 104.449 (3.872) | 105.521 (4.569) | 102.811 (3.969) | 101.570 (7.933) | 106.078 (4.804) | 103.839 (5.397) |  |
| Range | 91.500 - 122.750 | 89.615 - 119.500 | 89.750 - 125.400 | 89.500 - 117.000 | 72.000 - 117.000 | 93.000 - 119.000 | 72.000 - 125.400 |  |
| Chloride variance |  |  |  |  |  |  |  | < 0.001 |
| N-Miss | 0 | 0 | 0 | 1 | 0 | 0 | 1 |  |
| Mean (SD) | 2.559 (1.669) | 2.509 (1.273) | 2.461 (1.626) | 1.704 (1.433) | 2.858 (2.289) | 3.133 (1.796) | 2.471 (1.718) |  |
| Range | 0.000 - 7.859 | 0.000 - 6.440 | 0.000 - 11.481 | 0.000 - 6.944 | 0.000 - 12.021 | 0.000 - 8.340 | 0.000 - 12.021 |  |
| Protein (total) mean |  |  |  |  |  |  |  | < 0.001 |
| N-Miss | 0 | 3 | 0 | 12 | 3 | 0 | 18 |  |
| Mean (SD) | 55.064 (6.951) | 57.808 (6.034) | 47.232 (7.655) | 61.418 (7.168) | 54.328 (6.756) | 45.706 (7.694) | 54.274 (8.899) |  |
| Range | 43.000 - 70.000 | 38.000 - 72.556 | 28.444 - 66.000 | 36.000 - 80.000 | 39.000 - 70.000 | 31.857 - 68.500 | 28.444 - 80.000 |  |
| Protein (total) variance |  |  |  |  |  |  |  | < 0.001 |
| N-Miss | 0 | 3 | 0 | 12 | 3 | 0 | 18 |  |
| Mean (SD) | 2.742 (1.760) | 2.637 (2.096) | 4.941 (3.298) | 1.788 (1.879) | 2.126 (1.702) | 6.645 (5.723) | 3.298 (3.297) |  |
| Range | 0.000 - 7.760 | 0.000 - 10.592 | 0.000 - 13.809 | 0.000 - 7.500 | 0.000 - 6.500 | 0.000 - 24.366 | 0.000 - 24.366 |  |
| Fibrinogen mean |  |  |  |  |  |  |  | < 0.001 |
| N-Miss | 6 | 11 | 7 | 36 | 18 | 1 | 79 |  |
| Mean (SD) | 5.164 (2.210) | 3.048 (1.453) | 3.008 (1.397) | 3.608 (1.597) | 4.975 (1.921) | 2.405 (1.044) | 3.533 (1.816) |  |
| Range | 1.200 - 9.967 | 1.100 - 9.000 | 0.633 - 8.667 | 0.450 - 9.000 | 1.967 - 9.000 | 0.680 - 5.700 | 0.450 - 9.967 |  |
| Fibrinogen variance |  |  |  |  |  |  |  | < 0.001 |
| N-Miss | 6 | 11 | 7 | 36 | 18 | 1 | 79 |  |
| Mean (SD) | 0.316 (0.436) | 0.331 (0.564) | 0.607 (0.662) | 0.160 (0.370) | 0.167 (0.288) | 0.646 (0.584) | 0.368 (0.547) |  |
| Range | 0.000 - 1.842 | 0.000 - 3.300 | 0.000 - 3.333 | 0.000 - 2.733 | 0.000 - 1.306 | 0.000 - 2.610 | 0.000 - 3.333 |  |
| Phosphate mean |  |  |  |  |  |  |  | < 0.001 |
| N-Miss | 0 | 0 | 0 | 1 | 0 | 0 | 1 |  |
| Mean (SD) | 1.713 (0.497) | 1.001 (0.251) | 1.030 (0.268) | 1.026 (0.252) | 1.012 (0.270) | 1.171 (0.383) | 1.088 (0.353) |  |
| Range | 1.028 - 3.447 | 0.523 - 2.065 | 0.480 - 2.318 | 0.470 - 2.280 | 0.497 - 1.888 | 0.305 - 2.625 | 0.305 - 3.447 |  |
| Phosphate variance |  |  |  |  |  |  |  | < 0.001 |
| N-Miss | 0 | 0 | 0 | 1 | 0 | 0 | 1 |  |
| Mean (SD) | 0.318 (0.202) | 0.234 (0.143) | 0.229 (0.148) | 0.169 (0.136) | 0.232 (0.156) | 0.298 (0.221) | 0.233 (0.165) |  |
| Range | 0.000 - 1.065 | 0.000 - 0.912 | 0.000 - 0.823 | 0.000 - 0.684 | 0.000 - 0.755 | 0.000 - 1.319 | 0.000 - 1.319 |  |
| Gamma-GT mean |  |  |  |  |  |  |  | < 0.001 |
| N-Miss | 0 | 1 | 0 | 10 | 2 | 0 | 13 |  |
| Mean (SD) | 165.615 (274.013) | 84.175 (114.348) | 65.587 (95.862) | 69.018 (94.810) | 124.000 (156.495) | 121.351 (136.959) | 94.985 (140.567) |  |
| Range | 15.000 - 1608.800 | 7.000 - 1074.429 | 6.333 - 822.000 | 9.000 - 931.667 | 9.000 - 876.333 | 11.500 - 632.000 | 6.333 - 1608.800 |  |
| Gamma-GT variance |  |  |  |  |  |  |  | < 0.001 |
| N-Miss | 0 | 1 | 0 | 10 | 2 | 0 | 13 |  |
| Mean (SD) | 42.395 (84.816) | 22.608 (50.225) | 21.392 (34.372) | 8.027 (14.089) | 18.451 (33.746) | 43.674 (56.776) | 22.815 (46.280) |  |
| Range | 0.000 - 571.626 | 0.000 - 558.390 | 0.000 - 241.693 | 0.000 - 72.493 | 0.000 - 261.937 | 0.000 - 296.293 | 0.000 - 571.626 |  |
| Haemoglobin mean |  |  |  |  |  |  |  | < 0.001 |
| Mean (SD) | 5.824 (1.080) | 7.260 (1.144) | 6.224 (1.043) | 7.784 (1.019) | 6.344 (1.189) | 5.743 (0.979) | 6.736 (1.309) |  |
| Range | 4.450 - 8.986 | 4.600 - 9.600 | 4.067 - 10.425 | 5.000 - 10.350 | 4.325 - 10.850 | 4.233 - 8.267 | 4.067 - 10.850 |  |
| Haemoglobin variance |  |  |  |  |  |  |  | < 0.001 |
| Mean (SD) | 0.508 (0.274) | 0.618 (0.293) | 0.907 (0.462) | 0.453 (0.275) | 0.472 (0.276) | 0.875 (0.411) | 0.635 (0.383) |  |
| Range | 0.047 - 1.130 | 0.050 - 1.871 | 0.125 - 2.173 | 0.000 - 1.476 | 0.000 - 1.473 | 0.000 - 1.856 | 0.000 - 2.173 |  |
| Haematocrit mean |  |  |  |  |  |  |  | < 0.001 |
| Mean (SD) | 0.289 (0.048) | 0.356 (0.053) | 0.305 (0.052) | 0.384 (0.049) | 0.314 (0.056) | 0.282 (0.046) | 0.332 (0.063) |  |
| Range | 0.214 - 0.410 | 0.222 - 0.482 | 0.197 - 0.505 | 0.242 - 0.515 | 0.220 - 0.500 | 0.205 - 0.407 | 0.197 - 0.515 |  |
| Haematocrit variance |  |  |  |  |  |  |  | < 0.001 |
| Mean (SD) | 0.024 (0.013) | 0.030 (0.015) | 0.043 (0.023) | 0.022 (0.014) | 0.022 (0.014) | 0.044 (0.020) | 0.030 (0.019) |  |
| Range | 0.000 - 0.057 | 0.000 - 0.095 | 0.008 - 0.111 | 0.000 - 0.061 | 0.000 - 0.069 | 0.000 - 0.091 | 0.000 - 0.111 |  |
| Potassium mean |  |  |  |  |  |  |  | < 0.001 |
| N-Miss | 14 | 23 | 30 | 33 | 39 | 19 | 158 |  |
| Mean (SD) | 4.615 (0.938) | 3.915 (0.620) | 4.264 (0.608) | 4.207 (0.710) | 4.314 (1.393) | 4.436 (0.822) | 4.207 (0.861) |  |
| Range | 2.800 - 7.700 | 2.400 - 6.300 | 2.600 - 6.200 | 2.900 - 7.900 | 2.300 - 13.000 | 2.700 - 6.725 | 2.300 - 13.000 |  |
| Potassium variance |  |  |  |  |  |  |  | < 0.001 |
| N-Miss | 14 | 23 | 30 | 33 | 39 | 19 | 158 |  |
| Mean (SD) | 0.075 (0.160) | 0.041 (0.132) | 0.032 (0.093) | 0.051 (0.148) | 0.032 (0.102) | 0.305 (0.426) | 0.071 (0.201) |  |
| Range | 0.000 - 0.550 | 0.000 - 0.950 | 0.000 - 0.450 | 0.000 - 0.900 | 0.000 - 0.700 | 0.000 - 1.850 | 0.000 - 1.850 |  |
| Creatinine mean |  |  |  |  |  |  |  | < 0.001 |
| Mean (SD) | 313.757 (201.999) | 77.768 (30.062) | 91.256 (47.535) | 82.287 (44.400) | 99.912 (60.143) | 136.559 (92.968) | 109.734 (97.539) |  |
| Range | 98.333 - 1146.750 | 30.167 - 246.333 | 5.500 - 393.875 | 29.000 - 374.000 | 23.889 - 404.333 | 7.750 - 591.250 | 5.500 - 1146.750 |  |
| Creatinine variance |  |  |  |  |  |  |  | < 0.001 |
| Mean (SD) | 74.946 (114.554) | 11.034 (8.899) | 15.861 (16.897) | 8.428 (7.077) | 15.878 (16.409) | 31.362 (33.597) | 19.438 (39.442) |  |
| Range | 3.399 - 863.565 | 1.247 - 68.719 | 0.500 - 101.961 | 0.000 - 39.540 | 0.000 - 106.655 | 0.000 - 200.031 | 0.000 - 863.565 |  |
| LDH mean |  |  |  |  |  |  |  | < 0.001 |
| Mean (SD) | 441.418 (327.578) | 364.441 (252.849) | 342.113 (213.019) | 260.357 (115.342) | 298.294 (165.623) | 1105.611 (1217.987) | 416.123 (513.871) |  |
| Range | 111.667 - 1821.500 | 122.833 - 1473.333 | 112.000 - 1302.333 | 114.333 - 809.000 | 84.000 - 958.500 | 137.750 - 6250.200 | 84.000 - 6250.200 |  |
| LDH variance |  |  |  |  |  |  |  | < 0.001 |
| Mean (SD) | 116.103 (169.053) | 84.501 (132.422) | 87.621 (96.013) | 45.065 (67.624) | 53.240 (68.027) | 735.356 (1040.080) | 146.330 (414.752) |  |
| Range | 0.000 - 770.685 | 0.000 - 1337.691 | 0.000 - 581.075 | 0.000 - 480.845 | 0.000 - 501.384 | 0.000 - 5767.509 | 0.000 - 5767.509 |  |
| Leukocytes mean |  |  |  |  |  |  |  | < 0.001 |
| Mean (SD) | 13.644 (7.869) | 13.247 (6.733) | 13.742 (6.497) | 12.883 (4.659) | 14.442 (6.952) | 17.857 (19.545) | 14.009 (8.969) |  |
| Range | 0.000 - 41.440 | 1.867 - 88.517 | 1.800 - 59.825 | 3.000 - 31.500 | 0.000 - 34.227 | 1.760 - 170.000 | 0.000 - 170.000 |  |
| Leukocytes variance |  |  |  |  |  |  |  | < 0.001 |
| Mean (SD) | 3.005 (2.079) | 2.793 (1.690) | 3.628 (2.297) | 2.103 (1.481) | 3.414 (2.880) | 5.964 (9.589) | 3.284 (3.885) |  |
| Range | 0.000 - 10.748 | 0.100 - 12.302 | 0.386 - 11.669 | 0.000 - 8.939 | 0.000 - 17.990 | 0.000 - 78.885 | 0.000 - 78.885 |  |
| Magnesium mean |  |  |  |  |  |  |  | < 0.001 |
| Mean (SD) | 0.970 (0.181) | 0.852 (0.133) | 0.871 (0.173) | 0.808 (0.150) | 0.801 (0.139) | 0.852 (0.199) | 0.847 (0.163) |  |
| Range | 0.628 - 1.483 | 0.557 - 1.386 | 0.585 - 1.561 | 0.500 - 2.115 | 0.510 - 1.606 | 0.520 - 1.950 | 0.500 - 2.115 |  |
| Magnesium variance |  |  |  |  |  |  |  | < 0.001 |
| Mean (SD) | 0.124 (0.110) | 0.105 (0.077) | 0.145 (0.095) | 0.061 (0.055) | 0.107 (0.088) | 0.119 (0.087) | 0.107 (0.087) |  |
| Range | 0.000 - 0.546 | 0.000 - 0.459 | 0.000 - 0.479 | 0.000 - 0.233 | 0.000 - 0.586 | 0.000 - 0.454 | 0.000 - 0.586 |  |
| Sodium mean |  |  |  |  |  |  |  | < 0.001 |
| Mean (SD) | 138.638 (5.522) | 140.178 (3.219) | 139.390 (3.764) | 139.712 (3.302) | 136.919 (7.291) | 140.449 (4.160) | 139.299 (4.681) |  |
| Range | 126.500 - 154.400 | 130.250 - 152.500 | 126.750 - 150.737 | 128.000 - 153.000 | 110.250 - 152.250 | 129.667 - 155.375 | 110.250 - 155.375 |  |
| Sodium variance |  |  |  |  |  |  |  | < 0.001 |
| Mean (SD) | 2.376 (1.224) | 2.305 (1.340) | 2.328 (1.476) | 1.741 (1.261) | 2.697 (2.012) | 2.717 (1.450) | 2.313 (1.520) |  |
| Range | 0.373 - 5.550 | 0.433 - 7.176 | 0.000 - 10.055 | 0.000 - 7.482 | 0.000 - 10.852 | 0.000 - 6.387 | 0.000 - 10.852 |  |
| Thrombocytes mean |  |  |  |  |  |  |  | < 0.001 |
| Mean (SD) | 202.469 (123.966) | 233.249 (94.769) | 205.665 (93.239) | 242.764 (73.874) | 278.875 (150.123) | 161.580 (98.674) | 227.281 (110.063) |  |
| Range | 16.900 - 496.000 | 40.000 - 868.812 | 33.500 - 593.471 | 90.200 - 596.250 | 18.250 - 717.083 | 18.500 - 469.833 | 16.900 - 868.812 |  |
| Thrombocytes variance |  |  |  |  |  |  |  | < 0.001 |
| Mean (SD) | 34.293 (27.262) | 43.883 (44.551) | 53.524 (43.835) | 26.521 (21.520) | 42.162 (37.220) | 56.793 (67.253) | 42.618 (42.919) |  |
| Range | 0.000 - 177.667 | 0.500 - 283.056 | 2.062 - 305.059 | 0.000 - 166.432 | 0.000 - 209.163 | 4.922 - 507.755 | 0.000 - 507.755 |  |
| Urea mean |  |  |  |  |  |  |  | < 0.001 |
| Mean (SD) | 24.468 (8.930) | 7.073 (2.929) | 8.665 (4.535) | 6.965 (3.299) | 9.502 (4.567) | 11.157 (6.936) | 9.560 (6.607) |  |
| Range | 10.100 - 47.650 | 1.400 - 20.667 | 0.867 - 29.094 | 1.600 - 19.400 | 1.967 - 23.175 | 1.586 - 41.167 | 0.867 - 47.650 |  |
| Urea variance |  |  |  |  |  |  |  | < 0.001 |
| Mean (SD) | 5.307 (3.996) | 1.319 (0.896) | 1.991 (2.277) | 0.985 (0.937) | 1.719 (1.701) | 2.622 (2.360) | 1.899 (2.210) |  |
| Range | 0.000 - 18.464 | 0.050 - 4.787 | 0.000 - 15.387 | 0.000 - 5.467 | 0.000 - 8.742 | 0.000 - 11.289 | 0.000 - 18.464 |  |
| BMI |  |  |  |  |  |  |  | 0.011 |
| Mean (SD) | 28.770 (7.082) | 26.365 (4.269) | 25.916 (4.552) | 26.912 (5.093) | 27.021 (6.043) | 27.108 (5.533) | 26.768 (5.242) |  |
| Range | 16.300 - 57.370 | 16.330 - 42.300 | 13.870 - 45.910 | 15.790 - 43.670 | 14.460 - 50.810 | 18.730 - 41.800 | 13.870 - 57.370 |  |
| Previous ICU admission |  |  |  |  |  |  |  | 0.008 |
| Mean (SD) | 56 (91.8%) | 191 (95.5%) | 129 (86.6%) | 134 (84.8%) | 114 (86.4%) | 75 (86.2%) | 699 (88.8%) |  |
| Range | 5 (8.2%) | 9 (4.5%) | 20 (13.4%) | 24 (15.2%) | 18 (13.6%) | 12 (13.8%) | 88 (11.2%) |  |
| Systolic blood pressure |  |  |  |  |  |  |  | < 0.001 |
| N-Miss | 0 | 0 | 0 | 0 | 2 | 0 | 2 |  |
| Mean (SD) | 120.516 (25.092) | 122.435 (23.733) | 106.802 (16.913) | 135.892 (26.677) | 113.669 (21.702) | 109.523 (21.870) | 119.145 (24.925) |  |
| Range | 75.000 - 189.000 | 66.000 - 199.000 | 68.000 - 153.500 | 76.000 - 211.000 | 65.500 - 212.000 | 64.000 - 175.000 | 64.000 - 212.000 |  |
| Diastolic blood pressure |  |  |  |  |  |  |  | < 0.001 |
| N-Miss | 0 | 0 | 0 | 0 | 2 | 0 | 2 |  |
| Mean (SD) | 57.131 (10.563) | 63.215 (10.971) | 57.936 (9.157) | 71.038 (14.105) | 54.469 (8.475) | 57.833 (9.475) | 61.270 (12.209) |  |
| Range | 37.000 - 86.500 | 41.000 - 98.500 | 41.000 - 90.000 | 36.000 - 147.000 | 35.500 - 75.000 | 26.000 - 82.000 | 26.000 - 147.000 |  |
| Mean arterial pressure |  |  |  |  |  |  |  | < 0.001 |
| N-Miss | 0 | 0 | 0 | 1 | 2 | 0 | 3 |  |
| Mean (SD) | 76.902 (13.129) | 82.040 (13.847) | 73.074 (9.722) | 92.022 (16.972) | 72.519 (10.841) | 73.678 (12.354) | 79.429 (15.055) |  |
| Range | 51.000 - 114.000 | 44.000 - 130.000 | 52.000 - 107.000 | 55.000 - 168.000 | 39.500 - 96.000 | 32.500 - 104.000 | 32.500 - 168.000 |  |
| Atrial fibrillation |  |  |  |  |  |  |  | 0.151 |
| No | 55 (90.2%) | 187 (93.5%) | 141 (94.6%) | 145 (91.8%) | 119 (90.2%) | 86 (98.9%) | 733 (93.1%) |  |
| Yes | 6 (9.8%) | 13 (6.5%) | 8 (5.4%) | 13 (8.2%) | 13 (9.8%) | 1 (1.1%) | 54 (6.9%) |  |
| Heart rate at admission |  |  |  |  |  |  |  | < 0.001 |
| N-Miss | 0 | 2 | 2 | 1 | 0 | 1 | 6 |  |
| Mean (SD) | 97.000 (25.703) | 84.793 (20.080) | 98.531 (22.697) | 93.083 (24.074) | 103.530 (26.965) | 101.512 (22.961) | 95.006 (24.319) |  |
| Range | 45.000 - 162.000 | 48.000 - 141.000 | 55.000 - 173.000 | 40.000 - 171.000 | 53.000 - 207.000 | 56.000 - 157.000 | 40.000 - 207.000 |  |
| Urine output in previous 6 hours |  |  |  |  |  |  |  | < 0.001 |
| N-Miss | 4 | 2 | 2 | 4 | 2 | 1 | 15 |  |
| Mean (SD) | 0.540 (0.567) | 0.898 (0.698) | 0.910 (0.850) | 1.087 (0.876) | 0.988 (0.863) | 0.716 (0.678) | 0.906 (0.796) |  |
| Range | 0.000 - 2.013 | 0.028 - 4.935 | 0.000 - 4.357 | 0.085 - 5.689 | 0.026 - 4.841 | 0.000 - 3.429 | 0.000 - 5.689 |  |
| Central venous pressure |  |  |  |  |  |  |  | < 0.001 |
| No | 51 (83.6%) | 179 (89.5%) | 85 (57.0%) | 142 (89.9%) | 109 (82.6%) | 55 (63.2%) | 621 (78.9%) |  |
| Yes | 10 (16.4%) | 21 (10.5%) | 64 (43.0%) | 16 (10.1%) | 23 (17.4%) | 32 (36.8%) | 166 (21.1%) |  |
| Worsened respiratory condition |  |  |  |  |  |  |  | 0.003 |
| No | 47 (77.0%) | 188 (94.0%) | 131 (87.9%) | 140 (88.6%) | 108 (81.8%) | 76 (87.4%) | 690 (87.7%) |  |
| Yes | 14 (23.0%) | 12 (6.0%) | 18 (12.1%) | 18 (11.4%) | 24 (18.2%) | 11 (12.6%) | 97 (12.3%) |  |
| Tidal volume |  |  |  |  |  |  |  | 0.361 |
| N-Miss | 38 | 22 | 55 | 104 | 79 | 32 | 330 |  |
| Mean (SD) | 505.739 (73.665) | 517.753 (101.489) | 503.548 (138.640) | 537.741 (159.188) | 495.849 (92.017) | 495.036 (132.793) | 511.314 (119.798) |  |
| Range | 350.000 - 710.000 | 50.000 - 987.000 | 0.500 - 1130.000 | 263.000 - 1249.000 | 300.000 - 750.000 | 10.000 - 780.000 | 0.500 - 1249.000 |  |
| Respiratory rate |  |  |  |  |  |  |  | 0.003 |
| N-Miss | 39 | 23 | 54 | 105 | 79 | 30 | 330 |  |
| Mean (SD) | 15.000 (4.840) | 15.175 (3.818) | 16.369 (4.793) | 14.981 (4.517) | 15.792 (5.256) | 17.825 (5.448) | 15.795 (4.627) |  |
| Range | 8.000 - 24.000 | 8.000 - 28.000 | 2.600 - 27.000 | 8.000 - 27.000 | 6.000 - 32.000 | 10.000 - 36.000 | 2.600 - 36.000 |  |
| Positive end-expiratory pressure |  |  |  |  |  |  |  | < 0.001 |
| N-Miss | 38 | 22 | 54 | 102 | 78 | 30 | 324 |  |
| Mean (SD) | 9.261 (2.378) | 6.713 (2.337) | 7.668 (2.503) | 6.964 (1.972) | 7.713 (1.927) | 7.351 (2.125) | 7.261 (2.336) |  |
| Range | 5.000 - 14.000 | 2.000 - 15.000 | 4.000 - 15.000 | 5.000 - 12.000 | 5.000 - 12.000 | 5.000 - 12.000 | 2.000 - 15.000 |  |
| Mechanical ventilation after 24h |  |  |  |  |  |  |  | < 0.001 |
| No | 27 (44.3%) | 8 (4.0%) | 27 (18.1%) | 70 (44.3%) | 60 (45.5%) | 13 (14.9%) | 205 (26.0%) |  |
| Yes | 34 (55.7%) | 192 (96.0%) | 122 (81.9%) | 88 (55.7%) | 72 (54.5%) | 74 (85.1%) | 582 (74.0%) |  |
| Mechanical ventilation at admission |  |  |  |  |  |  |  | < 0.001 |
| No | 41 (67.2%) | 20 (10.0%) | 45 (30.2%) | 88 (55.7%) | 84 (63.6%) | 24 (27.6%) | 302 (38.4%) |  |
| Yes | 20 (32.8%) | 180 (90.0%) | 104 (69.8%) | 70 (44.3%) | 48 (36.4%) | 63 (72.4%) | 485 (61.6%) |  |
| Respiratory rate |  |  |  |  |  |  |  | < 0.001 |
| N-Miss | 0 | 1 | 2 | 0 | 0 | 0 | 3 |  |
| Mean (SD) | 21.541 (8.197) | 16.608 (4.735) | 18.129 (5.558) | 17.620 (4.772) | 19.591 (5.766) | 17.563 (5.722) | 18.089 (5.678) |  |
| Range | 9.000 - 58.000 | 8.000 - 42.000 | 5.000 - 35.000 | 7.000 - 33.000 | 10.000 - 45.000 | 7.000 - 36.000 | 5.000 - 58.000 |  |
| FiO2 low |  |  |  |  |  |  |  | 0.002 |
| N-Miss | 1 | 1 | 1 | 1 | 2 | 0 | 6 |  |
| Mean (SD) | 56.200 (27.526) | 46.839 (21.845) | 45.723 (21.455) | 42.299 (20.817) | 49.931 (24.022) | 47.437 (22.930) | 47.015 (22.760) |  |
| Range | 21.000 - 100.000 | 21.000 - 100.000 | 21.000 - 100.000 | 21.000 - 100.000 | 21.000 - 100.000 | 21.000 - 100.000 | 21.000 - 100.000 |  |
| Myocardial infarction (history) |  |  |  |  |  |  |  | < 0.001 |
| No | 59 (96.7%) | 171 (85.5%) | 132 (88.6%) | 151 (95.6%) | 129 (97.7%) | 82 (94.3%) | 724 (92.0%) |  |
| Yes | 2 (3.3%) | 29 (14.5%) | 17 (11.4%) | 7 (4.4%) | 3 (2.3%) | 5 (5.7%) | 63 (8.0%) |  |
| Diabetes (history) |  |  |  |  |  |  |  | 0.005 |
| No | 39 (63.9%) | 171 (85.5%) | 118 (79.2%) | 131 (82.9%) | 97 (73.5%) | 70 (80.5%) | 626 (79.5%) |  |
| Yes | 22 (36.1%) | 29 (14.5%) | 31 (20.8%) | 27 (17.1%) | 35 (26.5%) | 17 (19.5%) | 161 (20.5%) |  |
| Cardiovascular disease (history) |  |  |  |  |  |  |  | 0.025 |
| No | 55 (90.2%) | 194 (97.0%) | 136 (91.3%) | 149 (94.3%) | 128 (97.0%) | 86 (98.9%) | 748 (95.0%) |  |
| Yes | 6 (9.8%) | 6 (3.0%) | 13 (8.7%) | 9 (5.7%) | 4 (3.0%) | 1 (1.1%) | 39 (5.0%) |  |
| Chronic Obstructive pulmonary disease (history) |  |  |  |  |  |  |  | 0.017 |
| No | 55 (90.2%) | 184 (92.0%) | 128 (85.9%) | 127 (80.4%) | 114 (86.4%) | 81 (93.1%) | 689 (87.5%) |  |
| Yes | 6 (9.8%) | 16 (8.0%) | 21 (14.1%) | 31 (19.6%) | 18 (13.6%) | 6 (6.9%) | 98 (12.5%) |  |
| Respiratory insufficiency (history) |  |  |  |  |  |  |  | 0.023 |
| No | 61 (100.0%) | 195 (97.5%) | 135 (90.6%) | 148 (93.7%) | 124 (93.9%) | 84 (96.6%) | 747 (94.9%) |  |
| Yes | 0 (0.0%) | 5 (2.5%) | 14 (9.4%) | 10 (6.3%) | 8 (6.1%) | 3 (3.4%) | 40 (5.1%) |  |
| Chronic kidney disease (history) |  |  |  |  |  |  |  | < 0.001 |
| No | 33 (54.1%) | 199 (99.5%) | 144 (96.6%) | 152 (96.2%) | 126 (95.5%) | 79 (90.8%) | 733 (93.1%) |  |
| Yes | 28 (45.9%) | 1 (0.5%) | 5 (3.4%) | 6 (3.8%) | 6 (4.5%) | 8 (9.2%) | 54 (6.9%) |  |
| Dialysis (history) |  |  |  |  |  |  |  | 0.004 |
| No | 57 (93.4%) | 200 (100.0%) | 147 (98.7%) | 157 (99.4%) | 131 (99.2%) | 85 (97.7%) | 777 (98.7%) |  |
| Yes | 4 (6.6%) | 0 (0.0%) | 2 (1.3%) | 1 (0.6%) | 1 (0.8%) | 2 (2.3%) | 10 (1.3%) |  |
| Cirrhosis (history) |  |  |  |  |  |  |  | < 0.001 |
| No | 59 (96.7%) | 198 (99.0%) | 144 (96.6%) | 157 (99.4%) | 129 (97.7%) | 75 (86.2%) | 762 (96.8%) |  |
| Yes | 2 (3.3%) | 2 (1.0%) | 5 (3.4%) | 1 (0.6%) | 3 (2.3%) | 12 (13.8%) | 25 (3.2%) |  |
| Metastatic disease (history) |  |  |  |  |  |  |  | 0.154 |
| No | 59 (96.7%) | 197 (98.5%) | 144 (96.6%) | 153 (96.8%) | 127 (96.2%) | 80 (92.0%) | 760 (96.6%) |  |
| Yes | 2 (3.3%) | 3 (1.5%) | 5 (3.4%) | 5 (3.2%) | 5 (3.8%) | 7 (8.0%) | 27 (3.4%) |  |
| Haematological malignancy (history) |  |  |  |  |  |  |  | < 0.001 |
| No | 54 (88.5%) | 195 (97.5%) | 144 (96.6%) | 158 (100.0%) | 122 (92.4%) | 83 (95.4%) | 756 (96.1%) |  |
| Yes | 7 (11.5%) | 5 (2.5%) | 5 (3.4%) | 0 (0.0%) | 10 (7.6%) | 4 (4.6%) | 31 (3.9%) |  |
| Immune insufficiency (history) |  |  |  |  |  |  |  | < 0.001 |
| No | 40 (65.6%) | 192 (96.0%) | 127 (85.2%) | 142 (89.9%) | 103 (78.0%) | 72 (82.8%) | 676 (85.9%) |  |
| Yes | 21 (34.4%) | 8 (4.0%) | 22 (14.8%) | 16 (10.1%) | 29 (22.0%) | 15 (17.2%) | 111 (14.1%) |  |
