# Supplementary material for: Deep embedded clustering generalisability and adaptation for integrating mixed datatypes: two critical care cohorts
Source: Sci Rep. 2024 Jan 10;14:1045. doi: 10.1038/s41598-024-51699-z (PMC10781731; doi:10.1038/s41598-024-51699-z)
Supplement: Supplementary file 6 — Supplementary Table S7. [file 41598_2024_51699_MOESM6_ESM.docx]

**Table S7. Descriptive statistics of the input and outcome variables for the X-DEC clusters on the MUMC+ dataset.** The first column specifies the variables (in bold), and whether the variables is described by its mean with standard deviation (SD) and the range of values, or if it is a category, the number of patients in each level, and how much percentage of the patients fall in that level. If the variable is missing for some samples, this is indicated by ‘N-miss’, which specifies the number of samples for which this variable was missing. The other columns specify the different clusters, and complete dataset. The last column specifies the p-value. The Chi-square test was used for categorical variables, and the Kruskal-Wallis Rank Test for numeric variables.

|  | Cluster 1 (N=319) | Cluster 2 (N=1293) | Cluster 3 (N=383) | Cluster 4 (N=659) | Cluster 5 (N=463) | Cluster 6 (N=777) | Total (N=3894) | p value |
| --- | --- | --- | --- | --- | --- | --- | --- | --- |
| In-ICU mortality |  |  |  |  |  |  |  | < 0.001 |
| Survivor | 234 (73.4%) | 973 (75.3%) | 323 (84.3%) | 598 (90.7%) | 402 (86.8%) | 487 (62.7%) | 3017 (77.5%) |  |
| Non-survivor | 85 (26.6%) | 320 (24.7%) | 60 (15.7%) | 61 (9.3%) | 61 (13.2%) | 290 (37.3%) | 877 (22.5%) |  |
| Length of stay |  |  |  |  |  |  |  | < 0.001 |
| Mean (SD) | 8.795 (11.742) | 6.209 (7.417) | 8.553 (12.187) | 4.054 (5.671) | 5.214 (7.397) | 14.127 (19.406) | 7.748 (11.968) |  |
| Range | 1.010 - 68.371 | 1.003 - 113.493 | 1.007 - 73.743 | 1.003 - 69.945 | 1.026 - 115.438 | 1.003 - 188.375 | 1.003 - 188.375 |  |
| Apache IV mortality |  |  |  |  |  |  |  | < 0.001 |
| N-Miss | 76 | 207 | 98 | 118 | 133 | 144 | 776 |  |
| Mean (SD) | 0.436 (0.259) | 0.427 (0.284) | 0.296 (0.236) | 0.210 (0.216) | 0.292 (0.220) | 0.454 (0.282) | 0.369 (0.276) |  |
| Range | 0.017 - 0.984 | 0.004 - 0.982 | 0.007 - 0.973 | 0.002 - 0.996 | 0.002 - 0.959 | 0.003 - 0.994 | 0.002 - 0.996 |  |
| Apache IV score |  |  |  |  |  |  |  | < 0.001 |
| N-Miss | 76 | 207 | 98 | 118 | 133 | 144 | 776 |  |
| Mean (SD) | 92.852 (27.213) | 82.774 (29.923) | 74.639 (26.305) | 57.392 (24.297) | 71.785 (22.258) | 94.746 (33.259) | 79.679 (31.131) |  |
| Range | 31.000 - 188.000 | 12.000 - 190.000 | 14.000 - 166.000 | 9.000 - 195.000 | 26.000 - 181.000 | 11.000 - 217.000 | 9.000 - 217.000 |  |
| SAPS II score |  |  |  |  |  |  |  | < 0.001 |
| Mean (SD) | 50.724 (15.446) | 48.840 (15.352) | 39.640 (13.655) | 33.801 (12.766) | 39.819 (12.321) | 51.551 (17.524) | 45.013 (16.315) |  |
| Range | 19.000 - 99.000 | 14.000 - 101.000 | 6.000 - 84.000 | 6.000 - 92.000 | 11.000 - 87.000 | 11.000 - 113.000 | 6.000 - 113.000 |  |
| Post-operative |  |  |  |  |  |  |  | < 0.001 |
| No | 280 (87.8%) | 936 (72.4%) | 238 (62.1%) | 576 (87.4%) | 361 (78.0%) | 544 (70.0%) | 2935 (75.4%) |  |
| Yes | 39 (12.2%) | 357 (27.6%) | 145 (37.9%) | 83 (12.6%) | 102 (22.0%) | 233 (30.0%) | 959 (24.6%) |  |
| Admission diagnosis (Based on APACHE IV) |  |  |  |  |  |  |  | < 0.001 |
|  | 1 (0.3%) | 21 (1.6%) | 5 (1.3%) | 10 (1.5%) | 10 (2.2%) | 13 (1.7%) | 60 (1.5%) |  |
| Cardiovascular | 101 (31.7%) | 463 (35.8%) | 146 (38.1%) | 162 (24.6%) | 133 (28.7%) | 262 (33.7%) | 1267 (32.5%) |  |
| Gastrointestinal | 44 (13.8%) | 92 (7.1%) | 89 (23.2%) | 36 (5.5%) | 81 (17.5%) | 160 (20.6%) | 502 (12.9%) |  |
| Genito-urinary | 17 (5.3%) | 3 (0.2%) | 8 (2.1%) | 3 (0.5%) | 9 (1.9%) | 7 (0.9%) | 47 (1.2%) |  |
| Haematological | 25 (7.8%) | 15 (1.2%) | 15 (3.9%) | 23 (3.5%) | 19 (4.1%) | 61 (7.9%) | 158 (4.1%) |  |
| Metabolic | 22 (6.9%) | 15 (1.2%) | 6 (1.6%) | 20 (3.0%) | 10 (2.2%) | 35 (4.5%) | 108 (2.8%) |  |
| Musculoskeletal/skin | 3 (0.9%) | 5 (0.4%) | 6 (1.6%) | 1 (0.2%) | 3 (0.6%) | 5 (0.6%) | 23 (0.6%) |  |
| Neurological | 8 (2.5%) | 359 (27.8%) | 15 (3.9%) | 130 (19.7%) | 19 (4.1%) | 51 (6.6%) | 582 (14.9%) |  |
| Respiratory | 92 (28.8%) | 212 (16.4%) | 68 (17.8%) | 243 (36.9%) | 170 (36.7%) | 125 (16.1%) | 910 (23.4%) |  |
| Transplant | 2 (0.6%) | 0 (0.0%) | 2 (0.5%) | 0 (0.0%) | 0 (0.0%) | 7 (0.9%) | 11 (0.3%) |  |
| Trauma | 4 (1.3%) | 108 (8.4%) | 23 (6.0%) | 31 (4.7%) | 9 (1.9%) | 51 (6.6%) | 226 (5.8%) |  |
| Age |  |  |  |  |  |  |  | < 0.001 |
| N-Miss | 0 | 0 | 0 | 1 | 0 | 0 | 1 |  |
| Mean (SD) | 66.339 (12.410) | 63.179 (15.860) | 62.347 (14.988) | 61.283 (15.522) | 66.134 (13.571) | 60.145 (15.487) | 62.781 (15.255) |  |
| Range | 25.000 - 92.000 | 18.000 - 100.000 | 18.000 - 100.000 | 18.000 - 100.000 | 19.000 - 90.000 | 18.000 - 102.000 | 18.000 - 102.000 |  |
| Gender |  |  |  |  |  |  |  | 0.039 |
| Female | 91 (28.5%) | 474 (36.7%) | 134 (35.0%) | 249 (37.8%) | 182 (39.3%) | 287 (36.9%) | 1417 (36.4%) |  |
| Male | 228 (71.5%) | 819 (63.3%) | 249 (65.0%) | 410 (62.2%) | 281 (60.7%) | 490 (63.1%) | 2477 (63.6%) |  |
| vasoactive |  |  |  |  |  |  |  | < 0.001 |
| No | 77 (24.1%) | 176 (13.6%) | 48 (12.5%) | 403 (61.2%) | 147 (31.7%) | 68 (8.8%) | 919 (23.6%) |  |
| Yes | 242 (75.9%) | 1117 (86.4%) | 335 (87.5%) | 256 (38.8%) | 316 (68.3%) | 709 (91.2%) | 2975 (76.4%) |  |
| Renal replacement therapy |  |  |  |  |  |  |  | < 0.001 |
| No | 214 (67.1%) | 1252 (96.8%) | 363 (94.8%) | 645 (97.9%) | 437 (94.4%) | 599 (77.1%) | 3510 (90.1%) |  |
| Yes | 105 (32.9%) | 41 (3.2%) | 20 (5.2%) | 14 (2.1%) | 26 (5.6%) | 178 (22.9%) | 384 (9.9%) |  |
| NOR |  |  |  |  |  |  |  | < 0.001 |
| No | 234 (73.4%) | 973 (75.3%) | 323 (84.3%) | 598 (90.7%) | 402 (86.8%) | 487 (62.7%) | 3017 (77.5%) |  |
| Yes | 85 (26.6%) | 320 (24.7%) | 60 (15.7%) | 61 (9.3%) | 61 (13.2%) | 290 (37.3%) | 877 (22.5%) |  |
| ICU readmission |  |  |  |  |  |  |  | < 0.001 |
| 1st readmission | 44 (13.8%) | 69 (5.3%) | 32 (8.4%) | 67 (10.2%) | 69 (14.9%) | 54 (6.9%) | 335 (8.6%) |  |
| 2nd readmission | 6 (1.9%) | 8 (0.6%) | 5 (1.3%) | 16 (2.4%) | 12 (2.6%) | 8 (1.0%) | 55 (1.4%) |  |
| no readmission | 265 (83.1%) | 1210 (93.6%) | 342 (89.3%) | 569 (86.3%) | 372 (80.3%) | 713 (91.8%) | 3471 (89.1%) |  |
| EMV score |  |  |  |  |  |  |  | < 0.001 |
| N-Miss | 78 | 199 | 99 | 145 | 128 | 192 | 841 |  |
| Mean (SD) | 11.378 (4.855) | 3.772 (1.934) | 12.757 (4.002) | 12.905 (3.516) | 12.761 (3.920) | 8.140 (5.465) | 8.569 (5.458) |  |
| Range | 3.000 - 15.000 | 1.000 - 15.000 | 3.000 - 15.000 | 3.000 - 15.000 | 3.000 - 15.000 | 1.000 - 15.000 | 1.000 - 15.000 |  |
| Temperature centre |  |  |  |  |  |  |  | < 0.001 |
| N-Miss | 2 | 2 | 0 | 2 | 0 | 2 | 8 |  |
| Mean (SD) | 36.670 (1.447) | 35.761 (1.755) | 36.499 (1.851) | 36.609 (1.176) | 37.123 (1.292) | 35.939 (1.970) | 36.249 (1.723) |  |
| Range | 28.700 - 40.400 | 20.700 - 41.900 | 22.700 - 40.500 | 30.600 - 40.400 | 31.800 - 41.000 | 22.000 - 40.500 | 20.700 - 41.900 |  |
| Admission type |  |  |  |  |  |  |  | < 0.001 |
| Acute surgery | 22 (6.9%) | 368 (28.5%) | 116 (30.3%) | 52 (7.9%) | 79 (17.1%) | 216 (27.8%) | 853 (21.9%) |  |
| Medical | 297 (93.1%) | 925 (71.5%) | 267 (69.7%) | 607 (92.1%) | 384 (82.9%) | 561 (72.2%) | 3041 (78.1%) |  |
| ALAT mean |  |  |  |  |  |  |  | < 0.001 |
| N-Miss | 4 | 1 | 1 | 11 | 3 | 1 | 21 |  |
| Mean (SD) | 82.671 (170.260) | 70.572 (98.612) | 76.078 (147.774) | 55.971 (133.920) | 56.274 (109.020) | 357.492 (791.168) | 125.446 (388.582) |  |
| Range | 5.000 - 1728.333 | 6.500 - 1415.500 | 6.667 - 1836.875 | 7.000 - 2378.333 | 5.000 - 1232.000 | 5.500 - 8185.400 | 5.000 - 8185.400 |  |
| ALAT variance |  |  |  |  |  |  |  | < 0.001 |
| N-Miss | 4 | 1 | 1 | 11 | 3 | 1 | 21 |  |
| Mean (SD) | 26.321 (61.580) | 23.249 (40.642) | 33.544 (89.786) | 12.708 (44.520) | 15.671 (53.366) | 226.880 (532.623) | 62.650 (256.702) |  |
| Range | 0.000 - 484.002 | 0.000 - 530.855 | 0.000 - 981.677 | 0.000 - 658.500 | 0.000 - 965.906 | 0.000 - 4718.108 | 0.000 - 4718.108 |  |
| ASAT mean |  |  |  |  |  |  |  | < 0.001 |
| N-Miss | 3 | 1 | 1 | 11 | 3 | 2 | 21 |  |
| Mean (SD) | 99.429 (177.043) | 94.281 (144.391) | 117.186 (283.758) | 69.172 (124.719) | 66.613 (117.394) | 597.858 (1356.626) | 190.240 (656.773) |  |
| Range | 9.000 - 1507.250 | 10.500 - 2180.833 | 10.000 - 3285.600 | 7.500 - 1525.333 | 6.667 - 1881.000 | 8.000 - 12116.750 | 6.667 - 12116.750 |  |
| ASAT variance |  |  |  |  |  |  |  | < 0.001 |
| N-Miss | 3 | 1 | 1 | 11 | 3 | 2 | 21 |  |
| Mean (SD) | 40.311 (107.418) | 38.182 (87.137) | 61.077 (201.143) | 20.141 (56.672) | 22.165 (53.481) | 498.515 (1153.695) | 127.807 (555.795) |  |
| Range | 0.000 - 1047.713 | 0.000 - 1562.146 | 0.000 - 2251.907 | 0.000 - 714.671 | 0.000 - 598.467 | 0.000 - 8784.455 | 0.000 - 8784.455 |  |
| Albumin mean |  |  |  |  |  |  |  | < 0.001 |
| N-Miss | 44 | 244 | 46 | 140 | 66 | 67 | 607 |  |
| Mean (SD) | 20.932 (6.377) | 25.785 (6.061) | 19.281 (5.559) | 28.993 (6.315) | 20.902 (5.937) | 19.345 (5.982) | 23.238 (7.057) |  |
| Range | 7.600 - 40.900 | 8.600 - 43.167 | 7.150 - 36.450 | 12.733 - 48.300 | 7.442 - 38.800 | 4.200 - 41.633 | 4.200 - 48.300 |  |
| Albumin variance |  |  |  |  |  |  |  | < 0.001 |
| N-Miss | 44 | 244 | 46 | 140 | 66 | 67 | 607 |  |
| Mean (SD) | 1.323 (1.434) | 1.615 (1.802) | 1.619 (1.707) | 0.942 (1.435) | 0.976 (1.302) | 2.629 (2.035) | 1.627 (1.810) |  |
| Range | 0.000 - 6.663 | 0.000 - 14.114 | 0.000 - 9.300 | 0.000 - 9.772 | 0.000 - 7.797 | 0.000 - 11.202 | 0.000 - 14.114 |  |
| ALP mean |  |  |  |  |  |  |  | < 0.001 |
| N-Miss | 10 | 54 | 24 | 53 | 10 | 10 | 161 |  |
| Mean (SD) | 171.715 (194.164) | 100.346 (66.580) | 122.982 (104.366) | 107.706 (96.371) | 175.199 (212.827) | 155.415 (135.191) | 130.024 (131.422) |  |
| Range | 34.000 - 2114.286 | 16.500 - 952.273 | 22.500 - 1266.400 | 26.000 - 1626.538 | 21.000 - 2799.667 | 29.000 - 1691.000 | 16.500 - 2799.667 |  |
| ALP variance |  |  |  |  |  |  |  | < 0.001 |
| N-Miss | 10 | 54 | 24 | 53 | 10 | 10 | 161 |  |
| Mean (SD) | 38.918 (83.371) | 18.936 (34.764) | 29.013 (45.834) | 12.016 (49.136) | 26.984 (60.742) | 51.620 (72.271) | 28.128 (57.270) |  |
| Range | 0.000 - 835.732 | 0.000 - 555.904 | 0.000 - 429.861 | 0.000 - 884.012 | 0.000 - 909.332 | 0.000 - 786.392 | 0.000 - 909.332 |  |
| Bilirubin (total) mean |  |  |  |  |  |  |  | < 0.001 |
| N-Miss | 21 | 49 | 14 | 78 | 19 | 12 | 193 |  |
| Mean (SD) | 23.074 (53.460) | 11.080 (8.838) | 14.823 (20.162) | 11.067 (9.426) | 15.992 (30.096) | 33.151 (57.683) | 17.568 (34.351) |  |
| Range | 2.200 - 635.629 | 2.100 - 83.167 | 2.100 - 248.500 | 2.350 - 92.588 | 2.300 - 457.900 | 2.300 - 497.900 | 2.100 - 635.629 |  |
| Bilirubin (total) variance |  |  |  |  |  |  |  | < 0.001 |
| N-Miss | 21 | 49 | 14 | 78 | 19 | 12 | 193 |  |
| Mean (SD) | 4.138 (6.871) | 2.379 (3.102) | 3.606 (5.340) | 1.476 (2.680) | 2.479 (4.079) | 10.277 (18.441) | 4.146 (9.669) |  |
| Range | 0.000 - 57.050 | 0.000 - 27.258 | 0.000 - 40.355 | 0.000 - 31.150 | 0.000 - 38.219 | 0.000 - 163.082 | 0.000 - 163.082 |  |
| CK mean |  |  |  |  |  |  |  | < 0.001 |
| N-Miss | 29 | 71 | 20 | 107 | 53 | 33 | 313 |  |
| Mean (SD) | 693.783 (5051.332) | 691.732 (1448.488) | 648.348 (2469.534) | 427.011 (924.806) | 247.042 (446.865) | 1492.291 (3846.650) | 762.107 (2603.716) |  |
| Range | 10.000 - 85000.667 | 7.000 - 17582.200 | 7.500 - 39380.400 | 7.000 - 8004.444 | 7.500 - 4553.250 | 12.000 - 52764.714 | 7.000 - 85000.667 |  |
| CK variance |  |  |  |  |  |  |  | < 0.001 |
| N-Miss | 29 | 71 | 20 | 107 | 53 | 33 | 313 |  |
| Mean (SD) | 167.877 (578.282) | 320.620 (738.990) | 267.474 (656.711) | 172.418 (521.698) | 92.103 (221.838) | 1049.656 (2622.586) | 405.321 (1358.918) |  |
| Range | 0.000 - 8036.848 | 0.000 - 11958.044 | 0.000 - 7865.981 | 0.000 - 5634.855 | 0.000 - 2215.557 | 0.000 - 31656.416 | 0.000 - 31656.416 |  |
| CRP mean |  |  |  |  |  |  |  | < 0.001 |
| N-Miss | 0 | 9 | 2 | 4 | 0 | 2 | 17 |  |
| Mean (SD) | 150.234 (103.489) | 97.363 (69.643) | 131.586 (77.779) | 72.061 (73.304) | 175.303 (93.926) | 122.231 (78.337) | 115.081 (85.144) |  |
| Range | 7.200 - 586.250 | 1.000 - 431.667 | 1.000 - 480.214 | 1.000 - 440.250 | 2.000 - 541.667 | 1.667 - 598.333 | 1.000 - 598.333 |  |
| CRP variance |  |  |  |  |  |  |  | < 0.001 |
| N-Miss | 0 | 9 | 2 | 4 | 0 | 2 | 17 |  |
| Mean (SD) | 53.769 (41.653) | 50.769 (35.523) | 57.464 (37.807) | 26.881 (29.174) | 63.011 (41.927) | 57.077 (37.901) | 50.361 (38.307) |  |
| Range | 0.000 - 225.975 | 0.000 - 185.146 | 0.000 - 177.000 | 0.000 - 156.366 | 0.000 - 213.671 | 0.000 - 201.945 | 0.000 - 225.975 |  |
| Calcium mean |  |  |  |  |  |  |  | < 0.001 |
| N-Miss | 23 | 147 | 43 | 97 | 46 | 31 | 387 |  |
| Mean (SD) | 2.020 (0.212) | 2.049 (0.143) | 1.912 (0.157) | 2.140 (0.153) | 2.031 (0.157) | 1.919 (0.176) | 2.018 (0.179) |  |
| Range | 1.265 - 2.732 | 1.330 - 2.844 | 1.360 - 2.460 | 1.530 - 2.735 | 1.247 - 2.740 | 1.373 - 2.551 | 1.247 - 2.844 |  |
| Calcium variance |  |  |  |  |  |  |  | < 0.001 |
| N-Miss | 23 | 147 | 43 | 97 | 46 | 31 | 387 |  |
| Mean (SD) | 0.094 (0.076) | 0.059 (0.051) | 0.066 (0.061) | 0.046 (0.051) | 0.052 (0.053) | 0.119 (0.081) | 0.072 (0.068) |  |
| Range | 0.000 - 0.423 | 0.000 - 0.337 | 0.000 - 0.340 | 0.000 - 0.372 | 0.000 - 0.525 | 0.000 - 0.579 | 0.000 - 0.579 |  |
| Chloride mean |  |  |  |  |  |  |  | < 0.001 |
| N-Miss | 24 | 166 | 56 | 110 | 60 | 38 | 454 |  |
| Mean (SD) | 106.251 (7.946) | 108.027 (5.476) | 108.415 (5.527) | 105.287 (6.016) | 106.444 (6.378) | 110.035 (6.341) | 107.720 (6.310) |  |
| Range | 81.333 - 138.000 | 86.000 - 131.333 | 83.000 - 125.286 | 77.000 - 138.000 | 73.000 - 127.000 | 78.400 - 136.000 | 73.000 - 138.000 |  |
| Chloride variance |  |  |  |  |  |  |  | < 0.001 |
| N-Miss | 24 | 166 | 56 | 110 | 60 | 38 | 454 |  |
| Mean (SD) | 2.823 (2.454) | 2.303 (2.162) | 2.546 (2.473) | 1.544 (1.734) | 1.883 (1.972) | 3.605 (2.642) | 2.480 (2.352) |  |
| Range | 0.000 - 11.992 | 0.000 - 18.707 | 0.000 - 17.250 | 0.000 - 12.853 | 0.000 - 11.409 | 0.000 - 17.812 | 0.000 - 18.707 |  |
| Protein (total) mean |  |  |  |  |  |  |  | < 0.001 |
| N-Miss | 127 | 517 | 123 | 332 | 185 | 234 | 1518 |  |
| Mean (SD) | 53.495 (9.238) | 53.519 (7.823) | 46.565 (8.103) | 59.475 (7.980) | 52.146 (6.973) | 45.869 (9.604) | 51.667 (9.477) |  |
| Range | 31.800 - 85.300 | 29.000 - 100.912 | 20.550 - 70.000 | 33.100 - 89.600 | 25.400 - 83.200 | 12.500 - 77.500 | 12.500 - 100.912 |  |
| Protein (total) variance |  |  |  |  |  |  |  | < 0.001 |
| N-Miss | 127 | 517 | 123 | 332 | 185 | 234 | 1518 |  |
| Mean (SD) | 1.415 (2.182) | 1.292 (1.993) | 2.322 (3.034) | 0.729 (1.446) | 0.931 (1.563) | 3.348 (3.404) | 1.765 (2.611) |  |
| Range | 0.000 - 12.500 | 0.000 - 18.460 | 0.000 - 18.528 | 0.000 - 8.953 | 0.000 - 7.269 | 0.000 - 16.500 | 0.000 - 18.528 |  |
| Fibrinogen mean |  |  |  |  |  |  |  | < 0.001 |
| N-Miss | 103 | 387 | 80 | 317 | 195 | 112 | 1194 |  |
| Mean (SD) | 5.398 (1.878) | 3.648 (1.651) | 4.036 (1.763) | 4.392 (1.615) | 5.570 (1.890) | 3.425 (1.695) | 4.062 (1.864) |  |
| Range | 1.433 - 11.500 | 0.400 - 9.343 | 0.400 - 8.900 | 0.700 - 9.000 | 1.300 - 9.550 | 0.450 - 9.000 | 0.400 - 11.500 |  |
| Fibrinogen variance |  |  |  |  |  |  |  | < 0.001 |
| N-Miss | 103 | 387 | 80 | 317 | 195 | 112 | 1194 |  |
| Mean (SD) | 0.267 (0.435) | 0.298 (0.531) | 0.537 (0.646) | 0.092 (0.234) | 0.124 (0.289) | 0.699 (0.728) | 0.378 (0.591) |  |
| Range | 0.000 - 2.500 | 0.000 - 3.500 | 0.000 - 3.350 | 0.000 - 1.800 | 0.000 - 2.026 | 0.000 - 3.755 | 0.000 - 3.755 |  |
| Phosphate mean |  |  |  |  |  |  |  | < 0.001 |
| N-Miss | 7 | 67 | 23 | 38 | 12 | 11 | 158 |  |
| Mean (SD) | 1.646 (0.557) | 1.036 (0.274) | 1.055 (0.277) | 1.099 (0.299) | 1.035 (0.277) | 1.282 (0.437) | 1.150 (0.390) |  |
| Range | 0.618 - 3.870 | 0.270 - 2.900 | 0.450 - 2.510 | 0.400 - 2.680 | 0.270 - 2.313 | 0.270 - 3.349 | 0.270 - 3.870 |  |
| Phosphate variance |  |  |  |  |  |  |  | < 0.001 |
| N-Miss | 7 | 67 | 23 | 38 | 12 | 11 | 158 |  |
| Mean (SD) | 0.368 (0.275) | 0.202 (0.155) | 0.204 (0.151) | 0.154 (0.145) | 0.183 (0.159) | 0.341 (0.225) | 0.234 (0.197) |  |
| Range | 0.000 - 1.570 | 0.000 - 1.275 | 0.000 - 0.921 | 0.000 - 1.225 | 0.000 - 0.845 | 0.000 - 1.808 | 0.000 - 1.808 |  |
| Gamma-GT mean |  |  |  |  |  |  |  | < 0.001 |
| N-Miss | 8 | 34 | 14 | 32 | 10 | 6 | 104 |  |
| Mean (SD) | 149.114 (181.101) | 93.774 (119.325) | 105.966 (139.676) | 81.491 (111.306) | 177.112 (344.956) | 138.420 (140.861) | 116.514 (174.898) |  |
| Range | 6.750 - 1269.500 | 6.000 - 1477.250 | 7.000 - 1203.333 | 7.000 - 1550.143 | 9.400 - 4794.500 | 5.500 - 1163.955 | 5.500 - 4794.500 |  |
| Gamma-GT variance |  |  |  |  |  |  |  | < 0.001 |
| N-Miss | 8 | 34 | 14 | 32 | 10 | 6 | 104 |  |
| Mean (SD) | 42.185 (93.602) | 31.009 (64.872) | 33.630 (61.186) | 14.685 (60.661) | 31.028 (64.204) | 60.165 (82.309) | 35.414 (71.852) |  |
| Range | 0.000 - 1186.909 | 0.000 - 953.198 | 0.000 - 456.967 | 0.000 - 1072.133 | 0.000 - 734.265 | 0.000 - 619.918 | 0.000 - 1186.909 |  |
| Haemoglobin mean |  |  |  |  |  |  |  | < 0.001 |
| Mean (SD) | 5.896 (0.922) | 6.899 (1.177) | 5.893 (0.785) | 7.431 (1.309) | 6.216 (1.048) | 5.887 (0.902) | 6.525 (1.234) |  |
| Range | 4.463 - 9.825 | 4.471 - 11.320 | 4.050 - 9.460 | 4.360 - 11.850 | 4.594 - 10.473 | 4.116 - 10.270 | 4.050 - 11.850 |  |
| Haemoglobin variance |  |  |  |  |  |  |  | < 0.001 |
| Mean (SD) | 0.470 (0.252) | 0.616 (0.303) | 0.748 (0.356) | 0.447 (0.271) | 0.477 (0.250) | 0.769 (0.350) | 0.602 (0.328) |  |
| Range | 0.000 - 1.707 | 0.000 - 1.800 | 0.094 - 2.158 | 0.000 - 1.583 | 0.000 - 1.519 | 0.100 - 2.444 | 0.000 - 2.444 |  |
| Haematocrit mean |  |  |  |  |  |  |  | < 0.001 |
| N-Miss | 6 | 21 | 1 | 27 | 8 | 2 | 65 |  |
| Mean (SD) | 0.296 (0.046) | 0.340 (0.058) | 0.289 (0.039) | 0.367 (0.062) | 0.310 (0.052) | 0.293 (0.047) | 0.323 (0.060) |  |
| Range | 0.195 - 0.470 | 0.218 - 0.567 | 0.190 - 0.454 | 0.210 - 0.550 | 0.218 - 0.562 | 0.195 - 0.520 | 0.190 - 0.567 |  |
| Haematocrit variance |  |  |  |  |  |  |  | < 0.001 |
| N-Miss | 6 | 21 | 1 | 27 | 8 | 2 | 65 |  |
| Mean (SD) | 0.020 (0.013) | 0.027 (0.016) | 0.034 (0.019) | 0.018 (0.014) | 0.018 (0.013) | 0.035 (0.019) | 0.026 (0.017) |  |
| Range | 0.000 - 0.061 | 0.000 - 0.095 | 0.000 - 0.096 | 0.000 - 0.097 | 0.000 - 0.074 | 0.000 - 0.120 | 0.000 - 0.120 |  |
| Potassium mean |  |  |  |  |  |  |  | < 0.001 |
| N-Miss | 43 | 192 | 61 | 76 | 68 | 92 | 532 |  |
| Mean (SD) | 4.554 (0.779) | 4.136 (0.520) | 4.241 (0.578) | 4.195 (0.513) | 4.289 (0.649) | 4.481 (0.622) | 4.279 (0.605) |  |
| Range | 3.060 - 8.860 | 1.880 - 9.360 | 2.870 - 8.130 | 2.750 - 6.960 | 3.010 - 8.490 | 2.775 - 7.535 | 1.880 - 9.360 |  |
| Potassium variance |  |  |  |  |  |  |  | < 0.001 |
| N-Miss | 43 | 192 | 61 | 76 | 68 | 92 | 532 |  |
| Mean (SD) | 0.422 (0.308) | 0.272 (0.230) | 0.247 (0.213) | 0.249 (0.217) | 0.187 (0.180) | 0.607 (0.392) | 0.336 (0.307) |  |
| Range | 0.000 - 1.650 | 0.000 - 1.690 | 0.000 - 0.941 | 0.000 - 1.310 | 0.000 - 0.855 | 0.000 - 2.950 | 0.000 - 2.950 |  |
| Creatinine mean |  |  |  |  |  |  |  | < 0.001 |
| Mean (SD) | 338.105 (193.915) | 87.060 (45.036) | 101.244 (63.153) | 87.528 (51.050) | 103.221 (63.251) | 149.180 (95.442) | 123.417 (107.379) |  |
| Range | 56.800 - 1557.833 | 7.875 - 559.143 | 7.700 - 551.500 | 5.000 - 686.500 | 11.667 - 507.000 | 19.667 - 957.300 | 5.000 - 1557.833 |  |
| Creatinine variance |  |  |  |  |  |  |  | < 0.001 |
| Mean (SD) | 79.302 (68.822) | 12.902 (13.600) | 19.132 (22.052) | 9.605 (10.896) | 16.443 (21.353) | 40.609 (37.254) | 24.346 (35.178) |  |
| Range | 0.000 - 488.499 | 0.000 - 110.585 | 0.000 - 116.025 | 0.000 - 101.785 | 0.000 - 256.319 | 0.000 - 272.051 | 0.000 - 488.499 |  |
| LDH mean |  |  |  |  |  |  |  | < 0.001 |
| N-Miss | 5 | 27 | 2 | 47 | 19 | 8 | 108 |  |
| Mean (SD) | 349.370 (190.975) | 327.389 (241.562) | 357.693 (310.758) | 302.388 (203.714) | 299.694 (205.871) | 903.948 (1303.622) | 442.081 (665.884) |  |
| Range | 121.000 - 1312.667 | 100.500 - 3685.500 | 108.250 - 2958.600 | 81.333 - 1848.000 | 68.500 - 2861.000 | 101.750 - 13633.400 | 68.500 - 13633.400 |  |
| LDH variance |  |  |  |  |  |  |  | < 0.001 |
| N-Miss | 5 | 27 | 2 | 47 | 19 | 8 | 108 |  |
| Mean (SD) | 53.979 (86.902) | 58.725 (92.164) | 75.418 (142.577) | 40.989 (76.585) | 39.836 (68.567) | 515.188 (1058.790) | 147.644 (518.665) |  |
| Range | 0.000 - 790.474 | 0.000 - 1368.184 | 0.000 - 1395.242 | 0.000 - 706.159 | 0.000 - 739.737 | 0.000 - 7313.211 | 0.000 - 7313.211 |  |
| Leukocytes mean |  |  |  |  |  |  |  | < 0.001 |
| N-Miss | 4 | 1 | 7 | 4 | 0 | 7 | 23 |  |
| Mean (SD) | 12.292 (7.596) | 12.251 (4.429) | 12.282 (6.299) | 11.738 (5.589) | 14.288 (7.925) | 15.146 (17.953) | 12.990 (9.672) |  |
| Range | 0.133 - 98.537 | 0.338 - 47.083 | 0.200 - 36.475 | 0.200 - 77.075 | 0.100 - 63.140 | 0.100 - 417.750 | 0.100 - 417.750 |  |
| Leukocytes variance |  |  |  |  |  |  |  | < 0.001 |
| N-Miss | 4 | 1 | 7 | 4 | 0 | 7 | 23 |  |
| Mean (SD) | 2.958 (2.588) | 2.744 (1.824) | 3.627 (3.313) | 2.053 (1.882) | 3.251 (2.909) | 4.917 (6.280) | 3.223 (3.614) |  |
| Range | 0.000 - 18.644 | 0.000 - 21.224 | 0.000 - 29.957 | 0.000 - 24.228 | 0.000 - 20.612 | 0.000 - 102.892 | 0.000 - 102.892 |  |
| Magnesium mean |  |  |  |  |  |  |  | < 0.001 |
| N-Miss | 28 | 175 | 47 | 109 | 53 | 44 | 456 |  |
| Mean (SD) | 0.898 (0.174) | 0.824 (0.136) | 0.802 (0.134) | 0.813 (0.122) | 0.806 (0.139) | 0.851 (0.160) | 0.830 (0.145) |  |
| Range | 0.500 - 2.170 | 0.400 - 2.110 | 0.343 - 1.537 | 0.437 - 1.405 | 0.400 - 1.625 | 0.430 - 2.686 | 0.343 - 2.686 |  |
| Magnesium variance |  |  |  |  |  |  |  | < 0.001 |
| N-Miss | 28 | 175 | 47 | 109 | 53 | 44 | 456 |  |
| Mean (SD) | 0.079 (0.077) | 0.060 (0.058) | 0.079 (0.070) | 0.039 (0.047) | 0.068 (0.066) | 0.089 (0.070) | 0.067 (0.065) |  |
| Range | 0.000 - 0.789 | 0.000 - 0.495 | 0.000 - 0.416 | 0.000 - 0.290 | 0.000 - 0.486 | 0.000 - 0.871 | 0.000 - 0.871 |  |
| Sodium mean |  |  |  |  |  |  |  | < 0.001 |
| N-Miss | 43 | 188 | 65 | 76 | 77 | 95 | 544 |  |
| Mean (SD) | 140.601 (6.981) | 140.737 (4.486) | 140.238 (4.806) | 139.801 (4.918) | 138.560 (5.492) | 141.819 (5.307) | 140.485 (5.208) |  |
| Range | 121.000 - 169.750 | 116.714 - 160.000 | 125.400 - 157.643 | 112.000 - 164.000 | 108.500 - 152.160 | 122.833 - 159.467 | 108.500 - 169.750 |  |
| Sodium variance |  |  |  |  |  |  |  | < 0.001 |
| N-Miss | 43 | 188 | 65 | 76 | 77 | 95 | 544 |  |
| Mean (SD) | 2.681 (2.195) | 1.928 (1.930) | 2.165 (2.455) | 1.455 (1.569) | 1.393 (1.529) | 3.108 (2.104) | 2.109 (2.041) |  |
| Range | 0.000 - 9.417 | 0.000 - 14.134 | 0.000 - 17.913 | 0.000 - 13.411 | 0.000 - 8.592 | 0.000 - 14.900 | 0.000 - 17.913 |  |
| Thrombocytes mean |  |  |  |  |  |  |  | < 0.001 |
| N-Miss | 1 | 0 | 0 | 0 | 0 | 1 | 2 |  |
| Mean (SD) | 217.582 (129.311) | 228.904 (101.248) | 214.352 (144.119) | 242.087 (104.629) | 282.102 (150.649) | 187.031 (119.067) | 226.759 (122.138) |  |
| Range | 14.125 - 981.250 | 13.882 - 800.500 | 16.800 - 1580.000 | 15.200 - 777.750 | 12.871 - 1025.000 | 12.545 - 851.000 | 12.545 - 1580.000 |  |
| Thrombocytes variance |  |  |  |  |  |  |  | < 0.001 |
| N-Miss | 1 | 0 | 0 | 0 | 0 | 1 | 2 |  |
| Mean (SD) | 39.454 (34.976) | 46.068 (43.687) | 58.769 (60.188) | 28.534 (24.875) | 41.249 (34.555) | 74.480 (67.480) | 48.900 (49.696) |  |
| Range | 0.000 - 241.263 | 0.000 - 341.997 | 0.500 - 571.822 | 0.000 - 158.858 | 0.000 - 184.653 | 0.000 - 399.430 | 0.000 - 571.822 |  |
| Urea mean |  |  |  |  |  |  |  | < 0.001 |
| N-Miss | 0 | 1 | 0 | 1 | 0 | 0 | 2 |  |
| Mean (SD) | 24.525 (10.062) | 7.552 (4.333) | 9.015 (5.136) | 7.479 (4.034) | 9.188 (4.712) | 13.086 (7.384) | 10.374 (7.460) |  |
| Range | 7.033 - 72.300 | 1.367 - 36.837 | 1.133 - 43.175 | 1.733 - 30.300 | 1.000 - 29.871 | 1.575 - 46.340 | 1.000 - 72.300 |  |
| Urea variance |  |  |  |  |  |  |  | < 0.001 |
| N-Miss | 0 | 1 | 0 | 1 | 0 | 0 | 2 |  |
| Mean (SD) | 5.669 (4.156) | 1.463 (1.355) | 1.938 (2.042) | 1.086 (1.096) | 1.567 (1.468) | 3.795 (3.182) | 2.268 (2.616) |  |
| Range | 0.000 - 24.404 | 0.000 - 11.608 | 0.000 - 19.942 | 0.000 - 8.154 | 0.000 - 8.266 | 0.000 - 19.307 | 0.000 - 24.404 |  |
| BMI |  |  |  |  |  |  |  | < 0.001 |
| N-Miss | 37 | 126 | 34 | 83 | 49 | 55 | 384 |  |
| Mean (SD) | 27.972 (6.777) | 26.065 (5.311) | 25.598 (4.807) | 26.730 (5.878) | 26.108 (5.438) | 26.032 (5.075) | 26.279 (5.488) |  |
| Range | 14.500 - 66.400 | 13.100 - 72.000 | 12.600 - 51.000 | 13.800 - 82.700 | 11.400 - 62.400 | 13.100 - 52.000 | 11.400 - 82.700 |  |
| Previous ICU admission |  |  |  |  |  |  |  | < 0.001 |
| No | 265 (83.1%) | 1210 (93.6%) | 342 (89.3%) | 569 (86.3%) | 372 (80.3%) | 713 (91.8%) | 3471 (89.1%) |  |
| Yes | 54 (16.9%) | 83 (6.4%) | 41 (10.7%) | 90 (13.7%) | 91 (19.7%) | 64 (8.2%) | 423 (10.9%) |  |
| Systolic blood pressure |  |  |  |  |  |  |  | < 0.001 |
| N-Miss | 0 | 1 | 0 | 0 | 0 | 0 | 1 |  |
| Mean (SD) | 121.006 (34.543) | 127.094 (31.006) | 111.016 (28.187) | 150.311 (33.951) | 113.687 (29.311) | 117.098 (29.772) | 125.354 (33.556) |  |
| Range | 39.000 - 249.000 | 0.000 - 272.000 | 39.000 - 230.000 | 73.000 - 313.000 | 0.000 - 224.000 | 45.000 - 228.000 | 0.000 - 313.000 |  |
| Diastolic blood pressure |  |  |  |  |  |  |  | < 0.001 |
| N-Miss | 0 | 1 | 0 | 0 | 0 | 0 | 1 |  |
| Mean (SD) | 59.047 (18.258) | 65.815 (16.309) | 57.676 (16.108) | 86.135 (21.335) | 55.940 (15.482) | 63.071 (18.424) | 66.177 (20.179) |  |
| Range | 19.000 - 125.000 | 17.000 - 131.000 | 15.000 - 115.000 | 43.000 - 196.000 | 0.000 - 109.000 | 11.000 - 141.000 | 0.000 - 196.000 |  |
| Mean arterial pressure |  |  |  |  |  |  |  | < 0.001 |
| N-Miss | 0 | 1 | 0 | 0 | 0 | 0 | 1 |  |
| Mean (SD) | 59.047 (18.258) | 65.815 (16.309) | 57.676 (16.108) | 86.135 (21.335) | 55.940 (15.482) | 63.071 (18.424) | 66.177 (20.179) |  |
| Range | 19.000 - 125.000 | 17.000 - 131.000 | 15.000 - 115.000 | 43.000 - 196.000 | 0.000 - 109.000 | 11.000 - 141.000 | 0.000 - 196.000 |  |
| Atrial fibrillation |  |  |  |  |  |  |  | 0.022 |
| No | 281 (88.1%) | 1151 (89.0%) | 354 (92.4%) | 612 (92.9%) | 408 (88.1%) | 696 (89.6%) | 3502 (89.9%) |  |
| Yes | 38 (11.9%) | 142 (11.0%) | 29 (7.6%) | 47 (7.1%) | 55 (11.9%) | 81 (10.4%) | 392 (10.1%) |  |
| Heart rate at admission |  |  |  |  |  |  |  | < 0.001 |
| Mean (SD) | 99.044 (24.632) | 85.672 (23.579) | 98.713 (25.245) | 99.052 (26.059) | 104.171 (23.875) | 100.028 (26.004) | 95.379 (25.751) |  |
| Range | 35.000 - 164.000 | 1.000 - 215.000 | 47.000 - 203.000 | 37.000 - 213.000 | 50.000 - 198.000 | 35.000 - 250.000 | 1.000 - 250.000 |  |
| Urine output in previous 6 hours |  |  |  |  |  |  |  | < 0.001 |
| N-Miss | 26 | 16 | 13 | 47 | 10 | 31 | 143 |  |
| Mean (SD) | 0.392 (0.813) | 0.604 (0.524) | 0.498 (0.453) | 0.699 (0.641) | 0.562 (0.657) | 0.420 (0.471) | 0.551 (0.582) |  |
| Range | 0.000 - 12.000 | 0.000 - 5.800 | 0.000 - 3.200 | 0.000 - 6.700 | 0.000 - 7.700 | 0.000 - 5.220 | 0.000 - 12.000 |  |
| Central venous pressure |  |  |  |  |  |  |  | < 0.001 |
| No | 290 (90.9%) | 1111 (85.9%) | 296 (77.3%) | 644 (97.7%) | 432 (93.3%) | 588 (75.7%) | 3361 (86.3%) |  |
| Yes | 29 (9.1%) | 182 (14.1%) | 87 (22.7%) | 15 (2.3%) | 31 (6.7%) | 189 (24.3%) | 533 (13.7%) |  |
| Worsened respiratory condition |  |  |  |  |  |  |  | < 0.001 |
| No | 231 (72.4%) | 881 (68.1%) | 281 (73.4%) | 520 (78.9%) | 371 (80.1%) | 450 (57.9%) | 2734 (70.2%) |  |
| Yes | 88 (27.6%) | 412 (31.9%) | 102 (26.6%) | 139 (21.1%) | 92 (19.9%) | 327 (42.1%) | 1160 (29.8%) |  |
| Tidal volume |  |  |  |  |  |  |  | < 0.001 |
| N-Miss | 148 | 48 | 134 | 344 | 228 | 163 | 1065 |  |
| Mean (SD) | 429.743 (166.004) | 503.485 (145.899) | 483.016 (152.144) | 444.578 (220.592) | 438.362 (162.975) | 489.956 (147.229) | 482.321 (161.387) |  |
| Range | 0.000 - 971.000 | 0.000 - 1659.000 | 0.000 - 1000.000 | 0.000 - 1919.000 | 0.000 - 834.000 | 0.000 - 1280.000 | 0.000 - 1919.000 |  |
| Respiratory rate |  |  |  |  |  |  |  | < 0.001 |
| N-Miss | 195 | 109 | 157 | 462 | 284 | 205 | 1412 |  |
| Mean (SD) | 19.097 (6.753) | 16.514 (3.074) | 17.004 (3.456) | 16.751 (3.419) | 18.207 (4.414) | 17.612 (3.879) | 17.082 (3.763) |  |
| Range | 12.000 - 70.000 | 4.000 - 34.000 | 10.000 - 30.000 | 9.000 - 32.000 | 12.000 - 40.000 | 5.000 - 40.000 | 4.000 - 70.000 |  |
| Positive end-expiratory pressure |  |  |  |  |  |  |  | < 0.001 |
| N-Miss | 0 | 3 | 5 | 3 | 0 | 3 | 14 |  |
| Mean (SD) | 7.270 (3.146) | 7.426 (2.249) | 7.310 (2.542) | 6.209 (2.039) | 6.851 (2.561) | 7.730 (2.761) | 7.188 (2.525) |  |
| Range | 5.000 - 23.000 | 0.000 - 22.000 | 4.000 - 18.000 | 3.000 - 20.000 | 4.000 - 20.000 | 0.000 - 24.000 | 0.000 - 24.000 |  |
| Mechanical ventilation after 24h |  |  |  |  |  |  |  | < 0.001 |
| No | 193 (60.5%) | 367 (28.4%) | 220 (57.4%) | 476 (72.2%) | 313 (67.6%) | 212 (27.3%) | 1781 (45.7%) |  |
| Yes | 126 (39.5%) | 926 (71.6%) | 163 (42.6%) | 183 (27.8%) | 150 (32.4%) | 565 (72.7%) | 2113 (54.3%) |  |
| Mechanical ventilation at admission |  |  |  |  |  |  |  | < 0.001 |
| No | 264 (82.8%) | 571 (44.2%) | 273 (71.3%) | 572 (86.8%) | 374 (80.8%) | 514 (66.2%) | 2568 (65.9%) |  |
| Yes | 55 (17.2%) | 722 (55.8%) | 110 (28.7%) | 87 (13.2%) | 89 (19.2%) | 263 (33.8%) | 1326 (34.1%) |  |
| Respiratory rate |  |  |  |  |  |  |  | < 0.001 |
| Mean (SD) | 24.574 (10.680) | 17.626 (5.941) | 21.587 (7.441) | 22.135 (7.867) | 25.266 (11.439) | 20.748 (7.176) | 20.879 (8.364) |  |
| Range | 9.000 - 98.000 | 0.000 - 100.000 | 0.000 - 50.000 | 0.000 - 94.000 | 5.000 - 120.000 | 0.000 - 54.000 | 0.000 - 120.000 |  |
| FiO2 low |  |  |  |  |  |  |  | < 0.001 |
| N-Miss | 110 | 28 | 87 | 287 | 180 | 95 | 787 |  |
| Mean (SD) | 35.364 (12.874) | 30.051 (6.665) | 31.419 (8.467) | 34.481 (12.630) | 35.488 (12.669) | 30.710 (9.600) | 31.709 (9.710) |  |
| Range | 21.000 - 99.000 | 21.000 - 80.000 | 21.000 - 80.000 | 21.000 - 100.000 | 21.000 - 100.000 | 21.000 - 100.000 | 21.000 - 100.000 |  |
| Myocardial infarction (history) |  |  |  |  |  |  |  | < 0.001 |
| No | 297 (93.1%) | 1122 (86.8%) | 336 (87.7%) | 610 (92.6%) | 429 (92.7%) | 702 (90.3%) | 3496 (89.8%) |  |
| Yes | 22 (6.9%) | 171 (13.2%) | 47 (12.3%) | 49 (7.4%) | 34 (7.3%) | 75 (9.7%) | 398 (10.2%) |  |
| Diabetes (history) |  |  |  |  |  |  |  | < 0.001 |
| No | 238 (74.6%) | 1182 (91.4%) | 339 (88.5%) | 584 (88.6%) | 404 (87.3%) | 686 (88.3%) | 3433 (88.2%) |  |
| Yes | 81 (25.4%) | 111 (8.6%) | 44 (11.5%) | 75 (11.4%) | 59 (12.7%) | 91 (11.7%) | 461 (11.8%) |  |
| Cardiovascular disease (history) |  |  |  |  |  |  |  | < 0.001 |
| No | 277 (86.8%) | 1226 (94.8%) | 354 (92.4%) | 614 (93.2%) | 438 (94.6%) | 752 (96.8%) | 3661 (94.0%) |  |
| Yes | 42 (13.2%) | 67 (5.2%) | 29 (7.6%) | 45 (6.8%) | 25 (5.4%) | 25 (3.2%) | 233 (6.0%) |  |
| Chronic Obstructive pulmonary disease (history) |  |  |  |  |  |  |  | < 0.001 |
| No | 286 (89.7%) | 1192 (92.2%) | 347 (90.6%) | 541 (82.1%) | 395 (85.3%) | 710 (91.4%) | 3471 (89.1%) |  |
| Yes | 33 (10.3%) | 101 (7.8%) | 36 (9.4%) | 118 (17.9%) | 68 (14.7%) | 67 (8.6%) | 423 (10.9%) |  |
| Respiratory insufficiency (history) |  |  |  |  |  |  |  | < 0.001 |
| No | 307 (96.2%) | 1254 (97.0%) | 369 (96.3%) | 601 (91.2%) | 437 (94.4%) | 765 (98.5%) | 3733 (95.9%) |  |
| Yes | 12 (3.8%) | 39 (3.0%) | 14 (3.7%) | 58 (8.8%) | 26 (5.6%) | 12 (1.5%) | 161 (4.1%) |  |
| Chronic kidney disease (history) |  |  |  |  |  |  |  | NaN |
| No | 319 (100.0%) | 1293 (100.0%) | 383 (100.0%) | 659 (100.0%) | 463 (100.0%) | 777 (100.0%) | 3894 (100.0%) |  |
| Yes | 0 (0.0%) | 0 (0.0%) | 0 (0.0%) | 0 (0.0%) | 0 (0.0%) | 0 (0.0%) | 0 (0.0%) |  |
| Dialysis (history) |  |  |  |  |  |  |  | < 0.001 |
| No | 277 (86.8%) | 1288 (99.6%) | 379 (99.0%) | 658 (99.8%) | 455 (98.3%) | 760 (97.8%) | 3817 (98.0%) |  |
| Yes | 42 (13.2%) | 5 (0.4%) | 4 (1.0%) | 1 (0.2%) | 8 (1.7%) | 17 (2.2%) | 77 (2.0%) |  |
| Cirrhosis (history) |  |  |  |  |  |  |  | < 0.001 |
| No | 309 (96.9%) | 1266 (97.9%) | 378 (98.7%) | 651 (98.8%) | 458 (98.9%) | 741 (95.4%) | 3803 (97.7%) |  |
| Yes | 10 (3.1%) | 27 (2.1%) | 5 (1.3%) | 8 (1.2%) | 5 (1.1%) | 36 (4.6%) | 91 (2.3%) |  |
| Metastatic disease (history) |  |  |  |  |  |  |  | 0.002 |
| No | 302 (94.7%) | 1245 (96.3%) | 357 (93.2%) | 632 (95.9%) | 426 (92.0%) | 728 (93.7%) | 3690 (94.8%) |  |
| Yes | 17 (5.3%) | 48 (3.7%) | 26 (6.8%) | 27 (4.1%) | 37 (8.0%) | 49 (6.3%) | 204 (5.2%) |  |
| Haematological malignancy (history) |  |  |  |  |  |  |  | < 0.001 |
| No | 283 (88.7%) | 1266 (97.9%) | 360 (94.0%) | 630 (95.6%) | 433 (93.5%) | 696 (89.6%) | 3668 (94.2%) |  |
| Yes | 36 (11.3%) | 27 (2.1%) | 23 (6.0%) | 29 (4.4%) | 30 (6.5%) | 81 (10.4%) | 226 (5.8%) |  |
| Immune insufficiency (history) |  |  |  |  |  |  |  | < 0.001 |
| No | 273 (85.6%) | 1240 (95.9%) | 347 (90.6%) | 613 (93.0%) | 417 (90.1%) | 660 (84.9%) | 3550 (91.2%) |  |
| Yes | 46 (14.4%) | 53 (4.1%) | 36 (9.4%) | 46 (7.0%) | 46 (9.9%) | 117 (15.1%) | 344 (8.8%) |  |
